# Total Synthesis of Pulmonarin B and Design of Brominated Phenylacetic Acid/Tacrine Hybrids: Marine Pharmacophore Inspired Discovery of New ChE and Aβ Aggregation Inhibitors

**DOI:** 10.3390/md16090293

**Published:** 2018-08-21

**Authors:** Zhi-Qiang Cheng, Jia-Li Song, Kongkai Zhu, Juan Zhang, Cheng-Shi Jiang, Hua Zhang

**Affiliations:** 1 School of Biological Science and Technology, University of Jinan, Jinan 250022, China; czq13515312897@163.com (Z.-Q.C.); 13285419800@163.com (J.-L.S.); hkhhh.k@163.com (K.Z.); 2 School of Biological Sciences, University of Brasília, Brasília 72220-275, Brazil; zjandzq@163.com

**Keywords:** pulmonarin B, brominated-phenylacetic acid/tacrine hybrids, acetylcholinesterase, butylcholinesterase, anti-amyloid aggregation, Alzheimer’s disease

## Abstract

A marine natural product, pulmonarin B (**1**), and a series of related tacrine hybrid analogues were synthesized and evaluated as cholinesterase (ChE) inhibitors. The in vitro ChE assay results revealed that **1** showed moderate dual acetylcholinesterase (AChE)/ butyrylcholinesterase (BChE) inhibitory activity, while the hybrid **12j** proved to be the most potent dual inhibitor among the designed derivatives, being almost as active as tacrine. Molecular modeling studies together with kinetic analysis suggested that **12j** interacted with both the catalytic active site and peripheral anionic site of AChE. Compounds **1** and **12j** could also inhibit self-induced and AChE-induced Aβ aggregation. In addition, the cell-based assay against the human hepatoma cell line (HepG2) revealed that **1** and **12j** did not show significant hepatotoxicity compared with tacrine and donepezil. Taken together, the present study confirmed that compound **1** was a potential anti-Alzheimer’s disease (AD) hit, and **12j** could be highlighted as a multifunctional lead compound for anti-AD drug development.

## 1. Introduction

Alzheimer’s disease (AD) is a chronic neurodegenerative disorder that has become the third leading death cause behind cancer and cardiovascular diseases. AD worsens over the time with the loss of memory, decline in language skills, deficits in cognitive functions, and severe behavioral problems [1]. In 2015, about 1.9 million people died of AD [2], and in 2017, an estimated 46.8 million AD patients were diagnosed worldwide [3]. More seriously, the number is expected to triple by 2050 with the aging of the global population [4].

**Figure 1:**
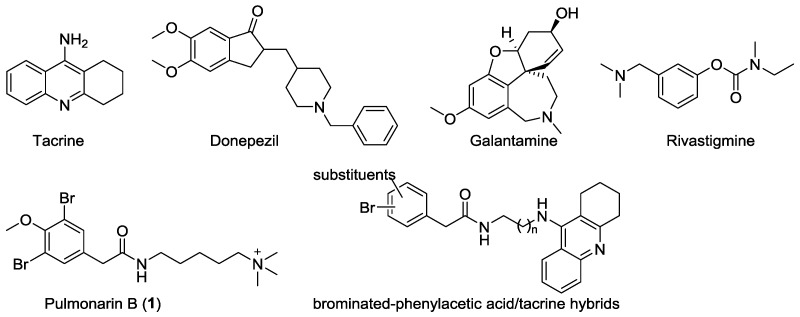
Structures of approved acetylcholinesterase (AChE) inhibitors, pulmonarin B (**1**), and brominated phenylacetic acid/tacrine hybrids.

Although the cause of AD is still poorly understood nowadays, many pathogenic hypotheses have been proposed over the last three decades [5,6]. Among them, cholinergic hypothesis is undoubtedly the earliest approved, which believes that increasing the level of acetylcholine (ACh) to enhance cholinergic neurotransmission in the brain is an efficacious approach for AD treatment [7]. In the brain, ACh can be hydrolyzed by two types of cholinesterase (ChE): acetylcholinesterase (AChE) and butyrylcholinesterase (BChE) [8]. AChE is the key enzyme for the termination of neurotransmission in cholinergic pathways via its rapid hydrolysis of ACh, almost 10^3^-fold more active than BChE [9]. Therefore, AChE inhibition is an effective approach for the symptomatic treatment for AD. As a result, four of five anti-AD drugs approved by the Food and Drug Administration (FDA) are AChE inhibitors including tacrine, donepezil, galantamine, and rivastigmine (Figure 1). These drugs are used to improve the memorial and cognitive functions of AD patients; however, none of them can stop or slow down the course of AD. Recent studies showed that the level and activity of BChE progressively increased in AD patients, while the activity of AChE declined [10]. BChE was found to serve as a backup to compensate for the function of AChE. Besides cholinergic hypothesis, amyloid hypothesis has been brought out to develop several therapeutic strategies for AD treatment. Among the factors involved in amyloid hypothesis, the formation and accumulation of beta-amyloid (Aβ) in the brain is considered as a primary target for developing novel anti-AD drugs, because of its ability to trigger critical intracellular signaling pathways associated with neurotoxicity, oxidative damage, and inflammation [11,12]. Due to the multifactorial nature of AD, multi-target directed bioactive molecules are believed to simultaneously modulate different targets involved in the progression of AD [13]. Thus, the dual inhibition of ChE and Aβ aggregation could be a promising strategy for the treatment of AD.

Marine natural products (MNPs) have been proven to be an extremely important source for developing novel drugs [14,15]. As a special group of MNPs, marine halogenated metabolites possess a wide range of biological properties, such as antibacterial, antiviral, antitumor, anti-inflammatory, and neurological activities [16,17]. During our continued project for developing new ChE inhibitors [18,19,20], pulmonarin B (**1**, Figure 1), a brominated phenylacetic acid derivative isolated from the ascidian *Synoicum pulmonaria* by Seveson et al. [21], came to our line of sight. This dibrominated compound was reported to be an AChE inhibitor (IC_50_ = 36 µM), and was considered as a marine hit for further studies [21]. It is important to note that the discovery of marine-derived AChE inhibitors was very limited according to the latest statistics of MNPs from 1984 to 2018 [22]. Thus, we recently prepared **1** using a synthetic approach that was different from that previously reported, and retested its anti-ChE effect. The assay results revealed that **1** was a moderate dual AChE/BChE inhibitor. In addition, it showed inhibitory activity against self-induced and AChE-induced Aβ_1–42_ aggregation and non-hepatotoxicity against HepG2 cells. However, the terminal quaternary ammonium group in **1** probably makes the compound’s polarity too high to pass the blood–brain barrier. To further increase the bioactive profiles of **1**, molecule hybridization was applied as a powerful strategy to assemble bioactive compounds. Tacrine as a versatile pharmacophore for the design of potent ChE inhibitors showed excellent dual inhibition on AChE/BChE with IC_50_ values at the nanomolar level [23,24,25]. Thus, a series of brominated phenylacetic acid/tacrine hybrids were designed by the fusion of tacrine to the terminal quaternary amine of **1**. In the present study, we describe the total synthesis of **1** and its brominated phenylacetic acid/tacrine hybrids, as well as evaluate their anti-AD potential, including AChE/BChE inhibition, molecular docking, anti-Aβ aggregation, and cytotoxicity.

## 2. Results and Discussion

### 2.1. Chemistry

**Scheme 1:**
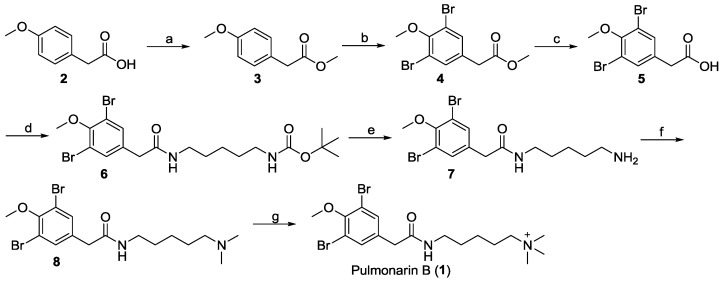
Synthesis of pulmonarin B (**1**). (a) Thionyl chloride, CH_3_OH, reflux; (b) *N*-bromosuccinimide (NBS), FeCl_3_, CH_3_CN, room temperature (r.t.); (c) CH_3_OH, H_2_O, NaOH, reflux; (d) 1-ethyl-3-(3-dimethylaminopropyl)carbodiimide (EDCI), hydroxybenzotriazole (HOBT), Et_3_N, CH_2_Cl_2_, *tert*-butyl (5-aminopentyl)carbamate, r.t., 8 h; (e) trifluoroacetic acid, CH_2_Cl_2_; (f) 37% formaldehyde solution, NaCNBH_3_, CH_3_OH, HCl (aq); (g) CH_3_OH, CH_3_I, K_2_CO_3_.

Given the particularly low yield of the key intermediate 3,5-(dibromo-4-methoxyphenyl)acetic acid in the protocol reported by Seveson et al. and the high price of the starting material 5-(dimethylamino) amylamine [21], the total synthetic route of pulmonarin B (**1**) was redesigned and successfully achieved with the agents in hand. As illustrated in Scheme 1, methyl 2-(4-methoxyphenyl) acetate (**3**) was prepared from the esterification of starting material 2-(4-methoxyphenyl) acetic acid (**2**). Ester **3** was dibrominated with NBS/FeCl_3_ in acetonitrile to give compound **4**, which was then hydrolyzed to yield acid **5**. The yield from **2** to **5** is 43%, which was twofold higher than that (21%) reported by Severson et al. Next, amide **6** was prepared by a coupling reaction of **5** with *tert*-butyl (5-aminopentyl)carbamate. The deprotection of the *N*-Boc group of **6** in trifluoroacetic acid directly gave amine **7**. Then, compound **8** was generated by the reductive amination of **7**. Finally, *N*-methylation of **8** with CH_3_I enabled the full synthesis of pulmonarin B (**1**). The total yield of **1** was increased to 3.5%, which was over twofold what was reported in the literature (1.6%) [21]. Also, the present synthetic route afforded more pulmonarin B analogues by modifying the terminal amino group of intermediate **7**. The ^1^H, ^13^C NMR and MS spectrascopic data of the synthetic **1** were in agreement with those of the natural **1** [21].

**Scheme 2:**
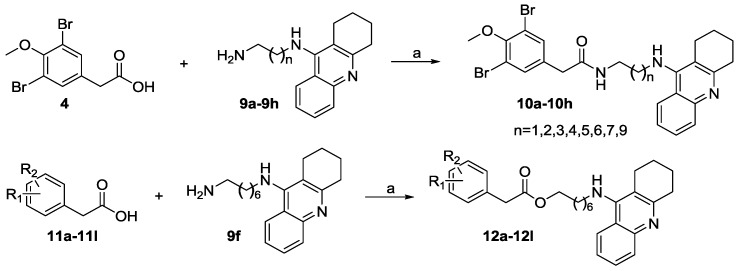
Synthesis of **10a**–**10h** and **12a**–**12l**. (a) EDCI, HOBT, Et_3_N, CH_2_Cl_2_, r.t., 8 h.

The synthetic routes for two series of compounds **10a**–**10h** and **12a**–**12l** were shown in Scheme 2. Briefly, intermediate **4** was reacted with appropriate amines to give the corresponding hybrids **10a**–**10h** (Table 1). Similarly, the coupling reaction of **9f** with different phenylacetic acids **11a**–**11l** afforded the corresponding **12a**–**12l** (Table 2).

### 2.2. In Vitro Inhibition of AChE and BChE, and Structure–Activity Relationship (SAR) Analysis

At first, the in vitro AChE and BChE inhibitory activities of **1** were tested. From the results in Table 1, compound **1** showed comparable AChE inhibitory activity (IC_50_ = 37.02 ± 2.11 µM) to the reported value of 36 µM [21]. In addition, it was also found to be a BChE inhibitor with an IC_50_ value of 30.70 ± 1.44 µM. However, the ChE inhibitory activity of **1** was much weaker than that of the positive control tacrine (AChE, IC_50_ = 0.159 ± 0.007 µM; BChE, IC_50_ = 0.046 ± 0.002 µM). Aiming to improve the activity of **1**, brominated phenylacetic acid/tacrine hybrids **10a**–**10h** were prepared based on molecule hybridization. Compared with **1**, all of these hybrids showed more potent ChE inhibitory activity, and clearly this improvement could be attributed to the introduction of tacrine moiety. In both enzyme bioassays, compound **10f** with 7-carbon aliphatic spacer in the linker showed the best activity (AChE, IC_50_ = 0.314 ± 0.010 µM; BChE, IC_50_ = 0.053 ± 0.007 µM). The preliminary SAR study indicated that the ChE inhibitory activities of these compounds increased with the elongation of the diamine linker (from **10a** to **10f**) and then decreased (from **10f** to **10h**), and the 7-carbon diamino linkage seemed to be the optimal distance between the brominated phenylacetyl and 1,2,3,4-tetrahydroacridine moieties.

Next, the substituent effect of benzene was investigated, and compound **10f** was selected as a model compound for further structural modification. Consequently, 12 analogues (**12a**–**12l**) were prepared, and their bioassay results were shown in Table 1. Among these analogues, compound **12j** showed the best ChE inhibitory activity (AChE, IC_50_ = 0.182 ± 0.006 µM; BChE, IC_50_ = 0.064 ± 0.006 µM), which was almost equal to that of tacrine. From the bioassay results, it was clear that different substituents had obvious impact on the AChE and BChE inhibitory activities. For example, compound **12j** with 5-Br and 2-F showed increased inhibitory activity toward AChE by fourfold compared with **12a**, and toward BChE by fourfold over **12f**. Compounds **12b** and **12l** without Br substitution showed low anti-ChE activity in comparison with **12j**.

### 2.3. Kinetic Study of AChE and BChE Inhibition

Herein, compound **12j** was selected as a representative prototype for kinetic assay to obtain information on the mode of action and binding site of this series of analogues. The mechanisms of AChE and BChE inhibitions were analyzed by recording substrate concentration−enzyme velocity curves in the presence of different concentrations of **12j**.

**Figure 2:**
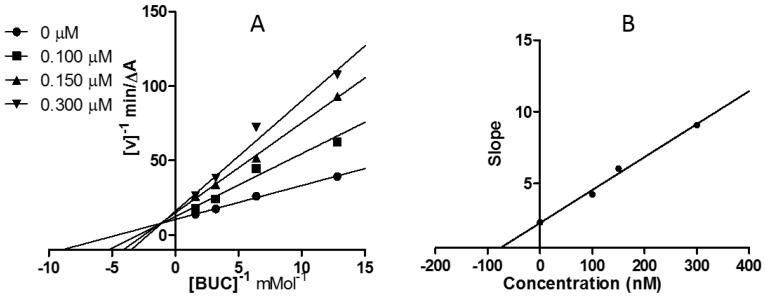
Kinetic assay on AChE inhibition displayed by **12j**. (**A**) Lineweaver–Burk reciprocal plots of initial velocity and increasing substrate concentrations (0.078–0.625 mM); (**B**) Secondary plot of slopes versus **12j** concentrations.

**Figure 3:**
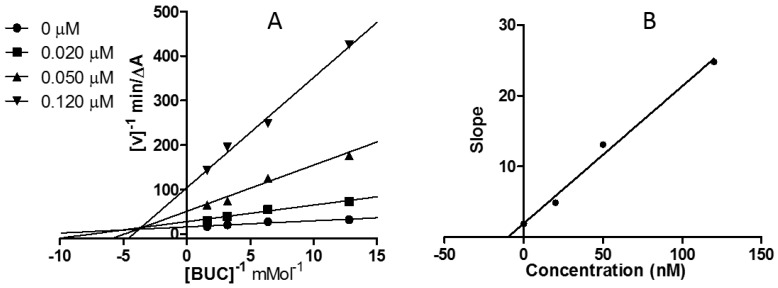
Kinetic assay on BChE inhibition displayed by **12j**. (**A**) Lineweaver–Burk reciprocal plots of initial velocity and increasing substrate concentrations (0.078–0.625 mM); (**B**) Secondary plot of slopes versus **12j** concentrations.

Graphical analyses revealed both increasing slopes (decreasing *V*_max_) and increasing intercepts (*K*_max_) at rising concentrations of **12j** (Figure 2A and Figure 3A). The equilibrium constant for binding with the free enzyme (*K*_i_) was obtained from the slope against inhibitor concentration [26]. The estimated *K*_i_ value of **12j** was 97.50 nM for AChE (Figure 2B), and that for BChE was 9.91 nM (Figure 3B). These patterns revealed compound **12j** as a linear mixed-type ChE inhibitor, indicating that **12j** may not only bind to the catalytic active site (CAS), but also interact with the peripheral anionic site (PAS) of both enzymes.

### 2.4. Docking Study

**Figure 4:**
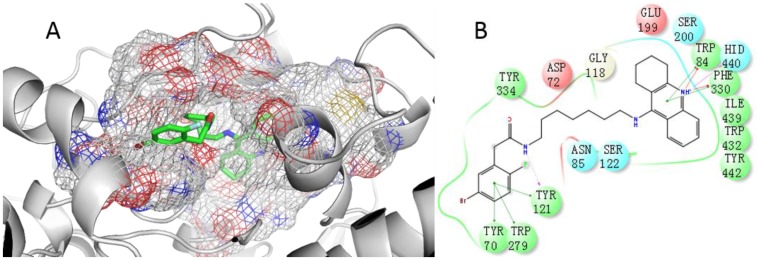
(**A**) The proposed orientation of **12j** (carbon in green) in the active pocket of AChE; (**B**) The interactions of **12j** with key amino acid residues in the active site of AChE.

As compound **12j** showed the best inhibition toward AChE, it was selected for docking simulation. First, the ligand alkylene-linked bis-tacrine that was derived from the original X-ray structure of AChE (PDB ID: 5EI5) was redocked to the protein to validate the molecular modeling methodology, as shown in Figure S1 (Supporting Information). The docking result could well reproduce the crystal structure, which indicated that the method was suitable for a docking study of AChE. Since the redocking of the ligand tacrine that originated from BChE (PDB ID: 4BDS) to its protein could not well reproduce the crystal structure, the binding mode of **12j** with BChE was not discussed in this study. As shown in Figure 4, **12j** was obviously able to bind to the active pocket of AChE. Here, AChE (PDB: 5EI5) [27] was used in the docking analysis because of its high resolution. The docking score for **12j**/AChE was −17.50. The tacrine moiety of **12j** could be spatially located in CAS (Figure 4A) and showed stacking interactions with Trp84 and Phe330 (Figure 4B). Furthermore, the protonated nitrogen atom in the tacrine ring underwent cation interaction with residues Phe330 and Trp84, and also formed an H-bond with the key residue His440. The brominated benzene moiety was oriented in the PAS by forming stacking interactions with Try70, Try121, and Trp279. The H atom of the amide group serves as an H-bond donor for interacting with Tyr121. In addition, hydrophobic interactions between **12j** and key residues also contributed to the affinity of **12j** to AChE.

### 2.5. Inhibition of Self-Induced and AChE-Induced Aβ Aggregation

Studies showed that ChE inhibitors not only increased the levels of ACh in the brain, they also reduced and prevented the formation of Aβ aggregation [28]. Compounds **1** and **12j** were assessed for their ability to inhibit self-induced and AChE-induced Aβ_1–42_ aggregation using the thioflavin T (ThT) fluorescence method. Tacrine and donepezil were used as positive reference compounds, and galantamine was included as a negative control. The results are shown in Table 2. The inhibitions of compound **1** and **12j** at 10 µM against self-induced Aβ_1–42_ aggregation were 29.78 ± 1.45% and 32.37 ± 0.62%, respectively, which were higher than that of donepezil (17.95 ± 0.77%). In addition, compounds **1** and **12j** also exhibited a significant inhibitory effect on AChE-induced Aβ aggregation, with inhibition ratios of 27.60 ± 1.96% and 47.73 ± 4.35%, respectively. As for galantamine, no significant inhibition was observed in self-induced and AChE-induced Aβ_1–42_ aggregation assay.

### 2.6. In Vitro Cytotoxicity toward HepG2 Cells

The main reason for the withdrawal of tacrine from the market is its hepatotoxicity. Thus, to verify the hepatotoxicity of **1** and **12j**, a 3-(4,5-dimethylthiazol-2-yl)-2,5-diphenyltetrazolium bromide (MTT) assay [29,30] on the human hepatoma cell line (HepG2) was carried out. Tacrine and donepezil were used as control drugs, and the IC_50_ values of all of the tested compounds toward HepG2 cells were summarized in Table 3. The results indicated that compounds **1** and **12j** did not show significant cytotoxicity against HepG2 cell (cell viability > 50% up to 80 µM) compared with tacrine and donepezil.

## 3. Materials and Methods 

### 3.1. General Information

Melting points were measured by a Melting Point YRY-3 apparatus (Tianjin Precision Apparatus Factory, Tianjin, China). Commercially available reagents were used without further purification. Organic solvents were evaporated with reduced pressure using Büchi R-100 evaporators. Reactions were monitored by thin-layer chromatography (TLC) using Yantai JingYou (Yantai, China) GF254 silica gel plates. Silica gel column chromatography was performed on an Isolera One system (Biotage, Uppsala, Sweden) with and silica gel (200–300 mesh) from Qingdao Hailang Inc. (Qingdao, China). NMR spectra were measured on a BrukerAvance III 600 spectrometer (Bruker, Fällanden, Switzerland). Chemical shifts were expressed in *δ* (ppm) and coupling constants (*J*) in Hz, with residual solvent signals as internal standards (CDCl_3_, *δ*_H_ 7.26 ppm and *δ*_C_ 77.0 ppm; DMSO-*d_6_*, *δ*_H_ 2.50 ppm and *δ*_C_ 39.5 ppm). Electron spray ionization (ESI)-MS analyses were performed on an Agilent 1260-6460 Triple Quard LC-MS instrument and HR-ESI-MS data were acquired on an Agilent Q-TOF 6520 spectrometer (Agilent, Waldbronn, Germany).

### 3.2. Chemistry

*Methyl 2-(4-methoxyphenyl) acetate* (**3**). Thionyl chloride (2.5 mg, 0.021 mmol, 0.003 equiv.) was added to a solution of 2-(4-methoxyphenyl) acetic acid (1156 mg, 7 mmol, 1 equiv.) in methanol (5 mL) at 0 °C. Then, the reaction solution was refluxed for 4 h. The solution was concentrated under reduced pressure to give **3**, which was used in the next step without further purification. Colorless oil. Yield: 1215 mg, 96.4%. ^1^H NMR (600 MHz, CDCl_3_) *δ* 7.20 (d, *J* = 8.7 Hz, 2H), 6.86 (d, *J* = 8.7 Hz, 2H), 3.79 (s, 3H), 3.68 (s, 3H), 3.57 (s, 2H). ^13^C NMR (150 MHz, CDCl_3_) *δ* 172.5, 158.8, 130.4, 126.2, 114.1, 55.4, 52.1, 40.4. ESI-MS *m*/*z* 203.0 [M + Na]^+^.

*2-(3,5-Dibromo-4-methoxyphenyl)acetate* (**4**). Ferric chloride (1097 mg, 6.75 mmol, 1 equiv.) and NBS (2403 mg, 13.5 mmol, 2 equiv.) were added to a solution of **3** (1215 mg, 6.75 mmol, 1 equiv.) in CH_3_CN at 0 °C. The mixture was stirred for 5 h at room temperature. After purification by flash chromatography, product **4** was obtained in moderate yield. Colorless oil. Yield: 1540 mg, 67.5%. ^1^H NMR (600 MHz, CDCl_3_) *δ* 7.43 (s, 2H), 3.87 (s, 3H), 3.71 (s, 3H), 3.53 (s, 2H). ^13^C NMR (150 MHz, CDCl_3_) *δ* 171.1, 153.5, 133.6, 132.5, 118.2, 60.8, 52.5, 39.7. ESI-MS *m*/*z* 335.9 [M + H]^+^.

*2-(3,5-Dibromo-4-methoxyphenyl)acetic acid* (**5**). A solution of **4** (1540 mg, 4.5 mmol, 1 equiv.) and NaOH (540 mg, 13.5 mmol, 3 equiv.) in CH_3_OH-H_2_O (*v*:*v* = 9:1, 10 mL) was refluxed for 24 h. Then, the reaction mixture was acidified with 1 M HCl and filtered. The obtained filter was washed three times with water to give compound **5**, which was used for the next step without further purification. White solid. Yield: 956 mg, 66.2%. Mp. 125.2–126.6 °C. ^1^H NMR (600 MHz, DMSO-*d*_6_) *δ* 12.51 (s, 1H, COOH), 7.57 (s, 2H), 3.78 (s, 3H), 3.60 (s, 2H). ^13^C NMR (150 MHz, DMSO-*d*_6_) *δ* 172.1, 152.0, 134.6, 133.9, 116.9, 60.4, 38.6. ESI-MS *m*/*z* 322.8 [M + H]^+^.

*Tert-butyl 5-(2-(3,5-dibromo-4-methoxyphenyl)acetamido)pentylcarbamate* (**6**). A 10-mL flask was charged with **5** (162 mg, 0.5 mmol, 1 equiv.), Et_3_N (151.5 mg, 4.5 mmol, 3 equiv.), HOBT (33.7 mg, 0.25 mmol, 0.5 equiv.), 1-ethyl-3-(3-dimethylaminopropyl)carbodiimide (EDCI) (143.2 mg, 0.75 mmol, 1.5 equiv.) and *tert*-butyl (5-aminopentyl)carbamate (101 mg, 0.5 mmol, 1.0 equiv.) under nitrogen atmosphere. After dissolution in 5 mL of anhydrous dichloromethane (DCM), the mixture was stirred at room temperature for 8 h. The organic phase was washed three times with water and dried over anhydrous MgSO_4_. The residue was purified by flash chromatography (petroleum ether:EtOAc = 1:1) to give product **6**. Colorless oil. Yield: 133 mg, 52.3%. ^1^H NMR (600 MHz, CDCl_3_) *δ* 7.43 (s, 2H), 5.80 (brs, 1H, NH), 3.86 (s, 3H), 3.43 (s, 2H), 3.24–3.21 (m, 2H), 3.12–3.08 (m, 2H), 1.52–1.47 (m, 4H), 1.43 (s, 9H), 1.33–1.29 (m, 2H). ^13^C NMR (150 MHz, CDCl_3_) *δ* 169.7, 156.3, 153.4, 133.9, 133.5, 118.4, 79.3, 60.7, 42.2, 40.3, 39.8, 29.9, 29.1, 28.6, 24.0. ESI-MS *m*/*z* 540.9 [M + Cl]^–^.

*Tert-butyl 5-(2-(3,5-dibromo-4-methoxyphenyl)acetamido)pentylcarbamate* (**8**). Compound **6** (127 mg, 0.25 mmol, 1 equiv.) was treated with trifluoroacetic acid (285 mg, 2.5 mmol, 10 equiv.) in 5 mL DCM at 0 °C to produce **7**, which was used in the next reaction without further purification. A solution of **7**, 37% formaldehyde solution (16.5 mg, 0.55 mmol, 2.5 equiv.), NaCNBH_3_ (41.4 mg, 0.66 mmol, 3 equiv.) and 12 M HCl(aq) (0.055 mL, 0.66 mmol, 3 equiv.) was stirred in CH_3_OH (5 mL) for 12 h. Then, the solution was concentrated, and the residue was purified by flash chromatography (CH_2_Cl_2_:CH_3_OH = 9:1) to give product **8**. Yellow oil. Yield: 90.3 mg, 94.2%. ^1^H NMR (600 MHz, CDCl_3_) *δ* 7.42 (s, 2H), 3.86 (s, 3H), 3.42 (s, 2H), 3.25–3.21 (m, 2H), 2.28–2.25 (m, 2H), 2.22 (s, 6H), 1.51–1.45 (m, 4H), 1.33–1.28 (m, 2H). ^13^C NMR (150 MHz, CDCl_3_) *δ* 169.7, 153.4, 133.9, 133.5, 118.4, 60.8, 59.4, 45.4, 42.3, 39.9, 29.2, 27.1, 24.6. ESI-MS *m*/*z* 435.0 [M + H]^+^.

*Pulmonarin B* (**1**). An amount of **8** (90 mg, 0.2 mmol, 1 equiv.) was dissolved in 5 mL of CH_3_OH, and then K_2_CO_3_ (57 mg, 0.4 mmol, 2 equiv.) was added. The resulting mixture was stirred at room temperature for 15 min before methyl iodide (31 mg, 0.22 mmol, 1.1 equiv.) was added. The reaction was stirred overnight, and then all of the volatiles were evaporated. A final purification of 10 mg of crude solid material gave purified **1** by using semipreparative reversed-phase HPLC (CH_3_OH/H_2_O/CH_3_COOH = 1:1:0.4 × 10^−4^). White solid. Yield: 1.5 mg, 16.6%. ^1^H NMR (600 MHz, DMSO-*d*_6_) *δ* 8.49 (t, *J* = 5.9 Hz, 1H, NH), 7.56 (s, 2H), 3.77 (s, 3H), 3.43 (s, 2H), 3.28–3.24 (m, 2H), 3.05 (s, 9H), 3.05–3.03 (m, 2H), 1.69–1.66 (m, 2H), 1.48–1.42 (m, 2H), 1.28–1.24 (m, 2H). ^13^C NMR (150 MHz, DMSO-*d*_6_) *δ* 169.3, 151.9, 136.2, 133.3, 116.9, 65.2, 65.2, 60.4, 52.1, 40.5, 38.3, 28.4, 23.2, 21.7. ESI-MS *m*/*z* 449.0 [M]^+^. HR-ESIMS: [M]^+^ calculated for C_17_H_27_Br_2_N_2_O_2_^+^ 449.0434, found 449.0439.

#### General Procedures for the Synthesis of **10a**–**10h** and **12a**–**12l**

For the synthesis of **10a**–**10h**, the mixtures of 2-(3,5-dibromo-4-methoxyphenyl)acetic acid (**4**, 80 mg, 0.25 mmol, 1 equiv.), hydroxybenzotriazole (HOBT) (19 mg, 0.125 mmol, 0.5 equiv.), EDCI (58 mg, 0.375 mmol, 1.5 equiv.), triethylamine (75 mg, 0.75 mmol, 3 equiv.), and corresponding amines **9a**–**9h** (0.25 mmol, 1 equiv.) in 5 mL of CH_2_Cl_2_ were stirred at room temperature overnight. For the synthesis of **12a**–**12l**, the mixtures of corresponding phenylacetic acids **11a**–**11l** (0.26 mmol, 1 equiv.), HOBT (20 mg, 0.13 mmol, 0.5 equiv.), EDCI (60 mg, 0.39 mmol, 1.5 equiv.), triethylamine (79 mg, 0.78 mmol, 3 equiv.), and *N*^1^-(1,2,3,4-tetrahydroacridin-9-yl)heptane-1,7-diamine (80 mg, 0.26 mmol, 1 equiv.) in 5 mL of CH_2_Cl_2_ were stirred at room temperature overnight. The above solutions were evaporated, and the residues were purified by flash chromatography using CH_2_Cl_2_:MeOH = 10:1 as an eluent to give the target compounds **10a**–**10h** and **12a**–**12l**.

*2-(3,5-Dibromo-4-methoxyphenyl)-N-(2-((1,2,3,4-tetrahydroacridin-9-yl)amino)ethyl)acetamide* (**10a**). White solid. Yield: 57.8 mg, 42.3%. Mp. 97.4–100.0 °C. ^1^H NMR (600 MHz, CDCl_3_) *δ* 9.11 (brs, 1H, NH), 8.19 (d, *J* = 8.4 Hz, 1H), 8.12 (d, *J* = 8.7 Hz, 1H), 7.52 (s, 2H), 7.45 (dd, *J* = 8.7, 7.5 Hz, 1H), 7.40 (brs, 1H, NH), 7.26 (dd, *J* = 8.4, 7.5 Hz,1H), 4.11–4.06 (m, 2H), 3.78 (s, 3H), 3.76–3.71 (m, 2H), 3.66 (s, 2H), 3.11 (t, *J* = 6.2 Hz, 2H), 2.48 (t, *J* = 6.1 Hz, 2H), 1.84–1.79 (m, 2H), 1.78–1.73 (m, 2H). ^13^C NMR (150 MHz, CDCl_3_) *δ* 173.4, 156.0, 153.1, 150.7, 138.6, 134.2, 133.5, 132.0, 124.9, 124.7, 120.2, 118.1, 115.7, 111.4, 60.7, 50.9, 41.3, 39.8, 28.5, 24.3, 22.1, 20.8. ESI-MS *m*/*z* 546.2 [M + H]^+^. HR-ESI-MS: [M + H]^+^ calculated for C_24_H_26_Br_2_N_3_O_2_^+^ 546.0386, found 546.0393.

*2-(3,5-Dibromo-4-methoxyphenyl)-N-(3-((1,2,3,4-tetrahydroacridin-9-yl)amino)propyl)acetamide* (**10b**). White solid. Yield: 51 mg, 36.4%. Mp. 72.3–73.1 °C. ^1^H NMR (600 MHz, CDCl_3_) *δ* 7.92 (d, *J* = 8.3 Hz, 1H), 7.88 (d, *J* = 8.4 Hz, 1H), 7.54 (dd, *J* = 8.4, 8.0 Hz, 1H), 7.39 (s, 2H), 7.34 (dd, *J* = 8.3, 8.0 Hz, 1H), 6.23 (brs, 1H, NH), 3.78 (s, 3H), 3.45 (s, 2H), 3.45–3.39 (m, 4H), 3.04 (brs, 2H), 2.71 (brs, 2H), 1.92–1.88 (m, 4H), 1.82–1.76 (m, 2H). ^13^C NMR (150 MHz, CDCl_3_) *δ* 170.5, 158.5, 153.5, 150.7, 147.1, 133.5, 133.5, 128.6, 128.5, 124.2, 122.6, 120.4, 118.5, 116.9, 60.7, 46.0, 42.2, 37.5, 33.9, 31.3, 25.1, 23.1, 22.8. ESI-MS *m*/*z* 560.0 [M + H]^+^. HR-ESI-MS: [M + H]^+^ calculated for C_25_H_28_Br_2_N_3_O_2_^+^ 560.0543, found 560.0594.

*2-(3,5-Dibromo-4-methoxyphenyl)-N-(4-((1,2,3,4-tetrahydroacridin-9-yl)amino)butyl)acetamide* (**10c**). White solid. Yield: 58.2 mg, 40.5%. Mp. 70.3–72.1 °C. ^1^H NMR (600 MHz, CDCl_3_) *δ* 8.25 (d, *J* = 8.1 Hz, 1H), 8.24 (d, *J* = 7.9 Hz, 1H), 8.09 (t, *J* = 5.5 Hz, 1H, NH), 7.59 (dd, *J* = 7.9, 7.5 Hz 1H), 7.47 (s, 2H), 7.39 (dd, *J* = 8.1, 7.5 Hz, 1H), 6.73 (brs, 1H, NH), 3.97–3.92 (m, 2H), 3.76 (s, 3H), 3.58 (s, 2H), 3.36–3.31 (m, 2H), 3.12 (t, *J* = 6.3 Hz, 2H), 2.66 (t, *J* = 6.2 Hz, 2H), 1.99–1.93 (m, 2H), 1.87–1.82 (m, 2H), 1.81–1.77 (m, 2H), 1.73–1.67 (m, 2H). ^13^C NMR (150 MHz, CDCl_3_) *δ* 170.8, 156.0, 152.8, 150.8, 138.7, 134.9, 133.5, 132.4, 125.3, 124.8, 120.2, 117.9, 115.9, 111.2, 60.7, 47.9, 41.8, 38.7, 28.6, 27.9, 26.3, 24.2, 22.1, 20.8. ESI-MS *m*/*z* 574.0 [M + H]^+^. HR-ESI-MS: [M + H]^+^ calculated for C_26_H_30_Br_2_N_3_O_2_^+^ 574.0699, found 574.0714.

*2-(3,5-Dibromo-4-methoxyphenyl)-N-(5-((1,2,3,4-tetrahydroacridin-9-yl)amino)pentyl)acetamide* (**10d**). White solid. Yield: 84.1 mg, 57.1%. Mp. 74.6–76.0 °C. ^1^H NMR (600 MHz, CDCl_3_) *δ* 7.93 (d, *J* = 8.4 Hz, 1H), 7.89 (d, *J* = 8.3 Hz, 1H), 7.54 (dd, *J* = 8.3, 7.1 Hz, 1H), 7.39 (s, 2H), 7.34 (dd, *J* = 8.4, 7.1 Hz, 1H), 5.69 (brs, 1H, NH), 3.85 (s, 3H), 3.45 (t, *J* = 7.2 Hz, 2H), 3.40 (s, 2H), 3.26–3.21 (m, 2H), 3.04 (brs, 2H), 2.69 (brs, 2H), 1.94–1.89 (m, 4H), 1.70–1.62 (m, 2H), 1.54–1.47 (m, 2H), 1.41–1.34 (m, 2H). ^13^C NMR (150 MHz, CDCl_3_) *δ* 169.7, 158.5, 153.4, 150.8, 147.4, 133.7, 133.5, 128.7, 128.5, 123.8, 122.9, 120.3, 118.5, 116.1, 60.8, 49.4, 42.2, 39.7, 34.1, 31.4, 29.5, 25.0, 24.3, 23.2, 22.9. ESI-MS *m*/*z* 588.1 [M + H]^+^. HR-ESI-MS: [M + H]^+^ calculated for C_27_H_32_Br_2_N_3_O_2_^+^ 588.0856, found 588.0853.

*2-(3,5-Dibromo-4-methoxyphenyl)-N-(6-((1,2,3,4-tetrahydroacridin-9-yl)amino)hexyl)acetamide* (**10e**). White solid. Yield: 98.6 mg, 65.4%. Mp. 72.1–74.0 °C. ^1^H NMR (600 MHz, CDCl_3_) *δ* 7.93 (d, *J* = 8.5 Hz, 1H), 7.88 (d, *J* = 8.2 Hz, 1H), 7.54 (dd, *J* = 8.2, 7.0 Hz, 1H), 7.39 (s, 2H), 7.33 (dd, *J* =8.5, 7.0 Hz, 1H), 5.73 (brs, 1H, NH), 3.85 (s, 3H), 3.45 (t, *J* = 7.2 Hz, 2H), 3.39 (s, 2H), 3.23–3.18 (m, 2H), 3.04 (brs, 2H), 2.69 (brs, 2H), 1.93–1.89 (m, 4H), 1.65–1.59 (m, 2H), 1.49–1.43 (m, 2H), 1.41–1.34 (m, 2H), 1.32–1.26 (m, 2H). ^13^C NMR (150 MHz, CDCl_3_) *δ* 169.7, 158.6, 153.4, 150.8, 147.6, 133.8, 133.5, 128.8, 128.4, 123.7, 123.0, 120.3, 118.4, 116.1, 60.7, 49.4, 42.2, 39.7, 34.2, 31.8, 29.5, 26.7, 26.6, 24.9, 23.2, 22.9. ESI-MS *m*/*z* 602.1 [M + H]^+^. HR-ESI-MS: [M + H]^+^ calculated for C_28_H_34_Br_2_N_3_O_2_^+^ 602.1012, found 602.1018.

*2-(3,5-Dibromo-4-methoxyphenyl)-N-(7-((1,2,3,4-tetrahydroacridin-9-yl)amino)heptyl)acetamide* (**10f**). White solid. Yield: 119 mg, 77.4%. Mp. 54.6–56.8 °C. ^1^H NMR (600 MHz, CDCl_3_) *δ* 8.34 (d, *J* = 8.6 Hz, 1H), 8.22 (d, *J* = 8.7 Hz, 1H), 7.62 (dd, *J* = 8.7, 7.4 Hz, 1H), 7.44 (s, 2H), 7.41 (dd, *J* = 8.6, 7.4 Hz, 1H), 7.21 (brs, 1H, NH), 3.87 (t, *J* = 7.2 Hz, 2H), 3.78 (s, 3H), 3.55 (s, 2H), 3.23–3.20 (m, 2H), 3.19 (t, *J* = 6.2 Hz, 2H), 2.65 (t, *J* = 6.1 Hz, 2H), 1.91–1.86 (m, 2H), 1.84–1.77 (m, 4H), 1.50–1.45 (m, 2H), 1.41–1.35 (m, 2H), 1.32–1.28 (m, 4H). ^13^C NMR (150 MHz, CDCl_3_) *δ* 170.3, 155.6, 152.9, 151.4, 139.4, 134.8, 133.5, 132.2, 125.1, 124.6, 121.0, 118.0, 116.2, 111.3, 60.7, 48.4, 41.9, 39.6, 30.7, 29.2, 28.9, 28.4, 26.4, 26.4, 24.1, 22.1, 20.9. ESI-MS *m*/*z* 616.1 [M + H]^+^. HR-ESI-MS: [M + H]^+^ calculated for C_29_H_36_Br_2_N_3_O_2_^+^ 616.1169, found 616.1180.

*2-(3,5-Dibromo-4-methoxyphenyl)-N-(8-((1,2,3,4-tetrahydroacridin-9-yl)amino)octyl)acetamide* (**10g**). White solid. Yield: 80.7 mg, 51.2%. Mp. 60.2–61.4 °C. ^1^H NMR (600 MHz, CDCl_3_) *δ* 7.96 (d, *J* = 8.4 Hz, 1H), 7.92 (d, *J* = 8.4 Hz, 1H), 7.55 (dd, *J* = 8.4, 7.4 Hz, 1H), 7.42 (s, 2H), 7.35 (dd, *J* = 8.4, 7.4 Hz, 1H), 5.59 (brs, 1H, NH), 3.86 (s, 3H), 3.50 (t, *J* = 7.2 Hz, 2H), 3.43 (s, 2H), 3.23–3.18 (m, 2H), 3.07 (brs, 2H), 2.70 (brs, 2H), 1.94–1.90 (m, 4H), 1.68–1.62 (m, 2H), 1.46–1.40 (m, 2H), 1.38–1.33 (m, 2H), 1.31– 1.23 (m, 6H). ^13^C NMR (150 MHz, CDCl_3_) *δ* 169.5, 158.1, 153.3, 151.1, 147.1, 133.7, 133.4, 128.5, 128.4, 123.7, 122.9, 120.0, 118.3, 115.6, 60.6, 49.3, 42.2, 39.8, 33.8, 31.7, 29.4, 29.1, 29.0, 26.7, 26.6, 24.7, 23.0, 22.7. ESI-MS *m*/*z* 630.1 [M + H]^+^. HR-ESI-MS: [M + H]^+^ calculated for C_30_H_38_Br_2_N_3_O_2_^+^ 630.1325, found 630.1330.

*2-(3,5-Dibromo-4-methoxyphenyl)-N-(10-((1,2,3,4-tetrahydroacridin-9-yl)amino)decyl)acetamide* (**10h**). White solid. Yield: 72.6 mg, 44.1%. Mp. 74.6–76.1 °C. ^1^H NMR (600 MHz, CDCl_3_) *δ* 7.96 (d, *J* = 8.4 Hz, 1H), 7.89 (d, *J* = 8.4 Hz, 1H), 7.54 (dd, *J* = 8.4, 7.4 Hz, 1H), 7.40 (s, 2H), 7.33 (dd, *J* = 8.4, 7.4 Hz, 1H), 6.01 (brs, 1H, NH), 3.84 (s, 3H), 3.49 (t, *J* = 7.1 Hz, 2H), 3.42 (s, 2H), 3.32–3.18 (m, 2H), 3.04 (brs, 2H), 2.69 (brs, 2H), 1.92–1.88 (m, 4H), 1.66–1.60 (m, 2H), 1.47–1.41 (m, 2H), 1.38–1.31 (m, 2H), 1.29–1.20 (m, 10H). ^13^C NMR (150 MHz, CDCl_3_) *δ* 169.6, 158.3, 153.3, 151.1, 147.3, 134.0, 133.5, 128.5, 128.5, 123.7, 123.1, 120.1, 118.3, 115.7, 60.7, 49.5, 42.2, 39.9, 33.9, 31.8, 29.5, 29.4, 29.4, 29.3, 29.2, 26.9, 26.9, 24.8, 23.1, 22.8. ESI-MS *m*/*z* 658.2 [M + H]^+^. HR-ESI-MS: [M + H]^+^ calculated for C_32_H_42_Br_2_N_3_O_2_^+^ 658.1638, found 658.1637.

*2-(2-Bromo-4-methoxyphenyl)-N-(7-((1,2,3,4-tetrahydroacridin-9-yl)amino)heptyl)acetamide* (**12a**). Yellow oil. Yield: 81.4 mg, 58.2% yield. ^1^H NMR (600 MHz, CDCl_3_) *δ* 8.41 (d, *J* = 8.5 Hz, 1H), 8.21 (d, *J* = 8.7 Hz, 1H), 7.63 (dd, *J* = 8.7, 7.6 Hz, 1H), 7.41 (dd, *J* = 8.5, 7.6 Hz, 1H), 7.20 (d, *J* = 8.5 Hz, 1H), 7.05 (d, *J* = 2.6 Hz, 1H), 6.80 (dd, *J* = 8.5, 2.6 Hz, 1H), 5.90 (brs, 1H, NH), 3.87 (t, *J* = 7.1 Hz, 2H), 3.75 (s, 3H), 3.63 (s, 2H), 3.23 (t, *J* = 6.2 Hz, 2H), 3.21–3.16 (m, 2H), 2.63 (t, *J* = 6.1 Hz, 2H), 1.91–1.86 (m, 2H), 1.85–1.77 (m, 2H), 1.47–1.42 (m, 2H), 1.41–1.36 (m, 2H), 1.35–1.31 (m, 2H). 1.31–1.25 (m, 4H). ^13^C NMR (150 MHz, CDCl_3_) *δ* 170.2, 159.4, 155.0, 151.6, 139.4, 132.2, 129.8, 127.1, 125.3, 125.1, 124.5, 121.3, 118.4, 116.1, 114.0, 111.1, 55.7, 48.4, 43.1, 39.5, 30.9, 29.8, 29.4, 28.6, 26.5, 26.5, 24.0, 22.1, 20.9. ESI-MS *m*/*z* 538.2 [M + H]^+^. HR-ESI-MS: [M + H]^+^ calculated for C_29_H_37_BrN_3_O_2_^+^ 538.2064, found 538.2060.

*2-(4-Methoxyphenyl)-N-(7-((1,2,3,4-tetrahydroacridin-9-yl)amino)heptyl)acetamide* (**12b**). Yellow oil. Yield: 43.4 mg, 36.3%. ^1^H NMR (600 MHz, CDCl_3_) *δ* 7.94 (d, *J* = 7.9 Hz, 1H), 7.89 (d, *J* = 8.4 Hz, 1H), 7.54 (dd, *J* = 8.4, 7.0 Hz, 1H), 7.33 (dd, *J* = 7.9, 7.0 Hz, 1H), 7.14 (d, *J* = 8.6 Hz, 2H), 6.86 (d, *J* = 8.6 Hz, 2H), 5.43 (brs, 1H, NH), 3.78 (s, 3H), 3.49 (s, 2H), 3.45 (t, *J* = 7.2 Hz, 2H), 3.18–3.14 (m, 2H), 3.05 (brs, 2H), 2.69 (brs, 2H), 1.93–1.88 (m, 4H), 1.64–1.58 (m, 2H), 1.41–1.36 (m, 2H), 1.35–1.31 (m, 2H), 1.30–1.25 (m, 2H), 1.24–1.18 (m, 2H). ^13^C NMR (150 MHz, CDCl_3_) *δ* 171.4, 158.9, 158.5, 150.9, 147.5, 130.6, 128.8, 128.4, 127.1, 123.7, 123.0, 120.3, 116.0, 114.5, 55.4, 49.5, 43.6, 39.6, 34.1, 31.8, 29.5, 29.0, 26.9, 26.7, 24.9, 23.1, 22.8. ESI-MS *m*/*z* 460.2 [M + H]^+^. HR-ESI-MS: [M + H]^+^ calculated for C_29_H_38_N_3_O_2_^+^ 460.2959, found 460.2950.

*2-(3-Bromo-4-methoxyphenyl)-N-(7-((1,2,3,4-tetrahydroacridin-9-yl)amino)heptyl)acetamide* (**12c**). Yellow oil. Yield: 50.8 mg, 36.3%. ^1^H NMR (600 MHz, CDCl_3_) *δ* 8.49 (d, *J* = 7.4 Hz, 1H), 8.16 (d, *J* = 8.7 Hz, 1H), 7.68 (dd, *J* = 8.7, 7.3 Hz, 1H), 7.43 (dd, *J* = 7.4, 7.3 Hz, 1H), 7.44 (d, *J* = 2.1 Hz, 1H), 7.18 (dd, *J* = 8.4, 2.1 Hz, 1H), 6.84 (d, *J* = 8.4 Hz, 1H), 5.86 (brs, 1H, NH), 3.87 (brs, 2H), 3.86 (s, 3H), 3.49 (s, 2H), 3.29 (t, *J* = 6.2 Hz, 2H), 3.23–3.18 (m, 2H), 2.60 (t, *J* = 6.0 Hz, 2H), 1.96–1.90 (m, 2H), 1.90–1.84 (m, 3H), 1.82–1.76 (m, 2H), 1.48–1.39 (m, 4H), 1.37–1.32 (m, 2H), 1.31–1.26 (m, 2H). ^13^C NMR (150 MHz, CDCl_3_) *δ* 171.1, 155.4, 154.9, 151.6, 139.6, 134.0, 132.0, 129.6, 129.3, 125.1, 124.5, 121.3, 116.3, 112.1, 111.7, 111.4, 56.4, 48.4, 42.3, 39.5, 30.8, 29.3, 29.0, 28.5, 26.5, 26.4, 24.1, 22.1, 20.9. ESI-MS *m*/*z* 538.1 [M + H]^+^. HR-ESI-MS: [M + H]^+^ calculated for C_29_H_37_BrN_3_O_2_^+^ 538.2064, found 538.2070.

*2-(2-Bromophenyl)-N-(7-((1,2,3,4-tetrahydroacridin-9-yl)amino)heptyl)acetamide* (**12d**). Yellow oil. Yield: 90.4 mg, 68.4%. ^1^H NMR (600 MHz, CDCl_3_) *δ* 8.21 (d, *J* = 8.1 Hz, 1H), 8.07 (d, *J* = 8.5 Hz, 1H), 7.60 (dd, *J* = 8.5, 7.3 Hz, 1H), 7.54 (dd, *J* = 8.0, 1.0 Hz, 1H), 7.38 (dd, *J* = 8.1, 7.3 Hz, 1H), 7.33 (dd, *J* = 7.6, 1.7Hz, 1H), 7.28 (ddd, *J* = 7.8, 7.6, 1.0 Hz, 1H), 7.13 (ddd, *J* = 8.0, 7.8, 1.7 Hz, 1H), 5.67 (brs, 1H, NH), 3.69 (s, 2H), 3.69–3.66 (m, 4H), 3.23–3.18 (m, 2H), 3.17 (t, *J* = 6.1 Hz, 2H), 2.64 (t, *J* = 6.0 Hz, 2H), 1.93–1.84 (m, 4H), 1.75–1.68 (m, 2H), 1.47–1.41 (m, 2H), 1.40–1.34 (m, 2H), 1.34–1.29 (m, 2H), 1.29–1.25 (m, 2H). ^13^C NMR (150 MHz, CDCl_3_) *δ* 169.7, 154.5, 153.5, 142.8, 135.2, 133.1, 131.8, 130.5, 129.1, 128.0, 127.7, 125.0, 124.5, 123.8, 117.9, 113.2, 48.9, 44.1, 39.6, 31.3, 31.0, 29.4, 28.7, 26.7, 26.6, 24.3, 22.5, 21.7. ESI-MS *m*/*z* 508.1 [M + H]^+^. HR-ESI-MS: [M + H]^+^ calculated for C_28_H_35_BrN_3_O^+^ 508.1958, found 508.1963.

*2-(3-Bromophenyl)-N-(7-((1,2,3,4-tetrahydroacridin-9-yl)amino)heptyl)acetamide* (**12e**). Yellow oil. Yield: 56.7 m, 42.9%. ^1^H NMR (600 MHz, CDCl_3_) *δ* 7.94 (d, *J* = 7.8 Hz, 1H), 7.89 (d, *J* = 8.4 Hz, 1H), 7.54 (dd, *J* = 8.4, 7.1 Hz, 1H), 7.41–7.36 (m, 2H), 7.33 (dd, *J* = 7.8, 7.1 Hz, 1H), 7.19–7.16 (m, 2H), 5.64 (brs, 1H, NH), 3.49 (s, 2H), 3.46(t, *J* = 7.2 Hz, 2H), 3.20–3.15 (m, 2H), 3.04 (brs, 2H), 2.69 (brs, 2H), 1.93–1.88 (m, 4H), 1.65–1.58 (m, 2H), 1.43–1.37 (m, 2H), 1.37–1.30 (m, 2H), 1.30–1.26 (m, 2H), 1.24–1.20 (m, 2H). ^13^C NMR (150 MHz, CDCl_3_) *δ* 170.1, 158.4, 151.0, 147.4, 137.5, 132.4, 130.5, 130.5, 128.6, 128.5, 128.1, 123.7, 123.0, 123.0, 120.2, 115.9, 49.5, 43.4, 39.7, 34.0, 31.7, 29.4, 29.0, 26.9, 26.7, 24.9, 23.1, 22.8. ESI-MS *m*/*z* 508.1 [M + H]^+^. HR-ESI-MS: [M + H]^+^ calculated for C_28_H_35_BrN_3_O^+^ 508.1958, found 508.1949.

*2-(4-Bromophenyl)-N-(7-((1,2,3,4-tetrahydroacridin-9-yl)amino)heptyl)acetamide* (**12f**). Yellow oil. Yield: 51.8 mg, 39.2%. ^1^H NMR (600 MHz, CDCl_3_) *δ* 7.93 (d, *J* = 8.5 Hz, 1H), 7.88 (d, *J* = 8.4 Hz, 1H), 7.53 (dd, *J* = 8.4, 7.0 Hz, 1H), 7.43 (d, *J* = 8.3 Hz, 2H), 7.33 (dd, *J* = 8.5, 7.0 Hz, 1H), 7.11 (d, *J* = 8.3 Hz, 2H), 5.57 (brs, 1H, NH), 3.47 (s, 2H), 3.45 (t, *J* = 7.3 Hz, 2H), 3.19–3.14 (m, 2H), 3.04 (brs, 2H), 2.69 (brs, 2H), 1.93–1.87 (m, 4H), 1.64–1.57 (m, 2H), 1.42–1.35 (m, 2H), 1.34–1.30 (m, 2H), 1.29–1.25 (m, 2H), 1.23–1.19 (m, 2H). ^13^C NMR (150 MHz, CDCl_3_) *δ* 170.3, 158.6, 150.8, 147.6, 134.2, 132.1, 131.1, 128.9, 128.4, 123.7, 122.9, 121.3, 120.4, 116.0, 49.5, 43.2, 39.7, 34.2, 31.8, 29.5, 29.0, 26.9, 26.7, 24.9, 23.2, 22.9. ESI-MS *m*/*z* 508.1 [M + H]^+^. HR-ESI-MS: [M + H]^+^ calculated for C_28_H_35_BrN_3_O^+^ 508.1958, found 508.1948.

*2-(2,5-Dibromophenyl)-N-(7-((1,2,3,4-tetrahydroacridin-9-yl)amino)heptyl)acetamide* (**12g**). Yellow oil, Yield: 54.5 mg, 35.7%. ^1^H NMR (600 MHz, CDCl_3_) *δ* 7.94 (d, *J* = 8.4 Hz, 1H), 7.89 (d, *J* = 8.3 Hz, 1H), 7.54 (dd, *J* = 8.3, 7.0 Hz, 1H), 7.49 (d, *J* = 2.4 Hz, 1H), 7.40 (d, *J* = 8.5 Hz, 1H), 7.34 (dd, *J* = 8.4, 7.0 Hz, 1H), 7.26 (dd, *J* = 8.5, 2.4 Hz,1H), 5.53 (brs, 1H, NH), 3.62 (s, 2H), 3.46 (t, *J* = 7.2 Hz, 2H), 3.24–3.18 (m, 2H), 3.05 (brs, 2H), 2.70 (brs, 2H), 1.94–1.89 (m, 4H), 1.65–1.59 (m, 2H), 1.46–1.41 (m, 2H), 1.38–1.33 (m, 2H), 1.32–1.27 (m, 2H), 1.27–1.23 (m, 2H). ^13^C NMR (150 MHz, CDCl_3_) *δ* 168.7, 158.6, 150.9, 147.6, 137.2, 134.5, 134.4, 132.2, 128.9, 128.4, 123.7, 123.6, 123.0, 121.8, 120.3, 116.0, 49.6, 43.9, 39.8, 34.2, 31.8, 29.5, 29.0, 26.9, 26.8, 24.9, 23.2, 22.9. ESI-MS *m*/*z* 586.0 [M + H]^+^. HR-ESI-MS: [M + H]^+^ calculated for C_28_H_34_Br_2_N_3_O^+^ 586.1063, found 586.1067.

*2-(3,5-Dibromophenyl)-N-(7-((1,2,3,4-tetrahydroacridin-9-yl)amino)heptyl)acetamide* (**12h**). Yellow oil. Yield: 56.6 mg, 37.1%. ^1^H NMR (600 MHz, CDCl_3_) *δ* 7.95 (d, *J* = 8.5 Hz, 1H), 7.90 (d, *J* = 8.4 Hz, 1H), 7.57 (t, *J* = 1.7 Hz, 1H), 7.55 (dd, *J* = 8.4, 7.1 Hz, 1H), 7.35 (d, *J* = 1.7 Hz, 2H), 7.34 (dd, *J* = 8.5, 7.1 Hz, 1H), 5.64 (brs, 1H, NH), 3.47 (d, *J* = 7.1 Hz, 2H), 3.45 (s, 2H), 3.22–3.17 (m, 2H), 3.05 (brs, 2H), 2.70 (brs, 2H), 1.93–1.90 (m, 4H), 1.65–1.60 (m, 2H), 1.44–1.39 (m, 2H), 1.38–1.33 (m, 2H), 1.32–1.28 (m, 2H), 1.27–1.24 (m, 2H). ^13^C NMR (150 MHz, CDCl_3_) *δ* 169.4, 158.5, 151.0, 147.5 139.0, 133.0, 131.2, 128.7, 128.5, 123.8, 123.3, 123.0, 120.3, 116.0, 49.5, 42.9, 39.9, 34.1, 31.8, 29.4, 29.0, 26.9, 26.8, 24.9, 23.2, 22.9. ESI-MS *m*/*z* 586.0 [M + H]^+^. HR-ESI-MS: [M + H]^+^ calculated for C_28_H_34_Br_2_N_3_O^+^ 586.1063, found 586.1065.

*2-(3-Bromo-4-fluorophenyl)-N-(7-((1,2,3,4-tetrahydroacridin-9-yl)amino)heptyl)acetamide* (**12i**). Yellow oil. Yield: 55.7 mg, 40.7%. ^1^H NMR (600 MHz, DMSO-*d_6_*) *δ* 8.39 (d, *J* = 8.7 Hz, 1H), 8.16 (t, *J* = 5.5 Hz, 1H, NH), 7.95 (d, *J* = 8.1 Hz, 1H), 7.84 (dd, *J* = 8.1, 7.5 Hz, 1H), 7.78 (brs, 1H, NH), 7.59–7.56 (m, 1H), 7.55 (dd, *J* = 8.7, 7.5 Hz, 1H), 7.28 (brs, 1H), 7.26 (d, *J* = 1.0 Hz, 1H), 3.85–3.79 (m, 2H), 3.40 (s, 2H), 3.04–2.97 (m, 4H), 2.65 (brs, 2H), 1.85–1.79 (m, 4H), 1.73–1.66 (m, 2H), 1.39–1.33 (m, 2H), 1.31–1.18 (m, 6H). ^13^C NMR (150 MHz, DMSO-*d_6_*) *δ* 169.3, 157.8, 156.2, 155.6, 150.6, 138.0, 134.8 (d, *J* = 3.57 Hz), 133.6, 132.5, 130.2 (d, *J* = 7.24 Hz), 125.0, 119.2, 116.3 (d, *J* = 21.93 Hz), 115.5, 111.1, 107.4 (d, *J* = 20.86 Hz), 47.2, 40.9, 38.5, 29.8, 28.9, 28.2, 28.0, 26.2, 26.0, 24.0, 21.5, 20.3. ESI-MS *m*/*z* 526.1 [M + H]^+^. HR-ESI-MS: [M + H]^+^ calculated for C_28_H_34_BrFN_3_O^+^ 526.1864, found 526.1868.

*2-(5-Bromo-2-fluorophenyl)-N-(7-((1,2,3,4-tetrahydroacridin-9-yl)amino)heptyl)acetamide* (**12j**). Yellow oil. Yield: 47.9 mg, 35.0%. ^1^H NMR (600 MHz, CDCl_3_) *δ* 8.03–7.96 (m, 2H), 7.56 (dd, *J* = 8.1, 7.1 Hz, 1H), 7.44 (dd, *J* = 6.6, 2.5 Hz, 1H), 7.37–7.32 (m, 2H), 6.93 (t, *J* = 9.0 Hz, 1H), 5.75 (brs, 1H, NH), 3.55 (t, *J* = 7.1 Hz, 2H), 3.51 (s, 2H), 3.23–3.19 (m, 2H), 3.08 (brs, 2H), 2.67 (brs, 2H), 1.92–1.88 (m, 4H), 1.69–1.62 (m, 2H), 1.47–1.41 (m, 2H), 1.39–1.34 (m, 2H), 1.33–1.29 (m, 2H), 1.29–1.26 (m, 2H). ^13^C NMR (150 MHz, CDCl_3_) *δ* 169.1, 160.9, 159.3, 151.9, 145.7, 134.4 (d, *J* = 4.03 Hz), 132.1 (d, *J* = 8.21 Hz), 129.3, 127.2, 124.8 (d, *J* = 17.42 Hz), 124.0, 123.3, 119.4, 117.3 (d, *J* = 23.63 Hz), 117.0 (d, *J* = 3.36 Hz), 114.9, 49.3, 39.8, 36.7, 32.9, 31.6, 29.4, 28.9, 26.8, 26.6, 24.7, 22.9, 22.5. ESI-MS *m*/*z* 526.1 [M + H]^+^. HR-ESI-MS: [M + H]^+^ calculated for C_28_H_34_BrFN_3_O^+^ 526.1864, found 526.1867.

*2-(2-Bromo-5-chlorophenyl)-N-(7-((1,2,3,4-tetrahydroacridin-9-yl)amino)heptyl)acetamide* (**12k**). Yellow oil. Yield: 70.4 mg, 49.9%. ^1^H NMR (600 MHz, CDCl_3_) *δ* 8.37 (d, *J* = 8.5 Hz, 1H), 8.23 (d, *J* = 8.7 Hz, 1H), 7.63 (dd, *J* = 8.7, 7.6 Hz, 1H), 7.47 (d, *J* = 2.0 Hz, 1H), 7.41 (dd, *J* = 8.5, 7.6 Hz, 1H), 7.25 (d, *J* = 8.2 Hz, 1H), 7.18 (dd, *J* = 8.2, 2.0 Hz, 1H), 6.50 (brs, 1H, NH), 3.93–3.86 (m, 2H), 3.69 (s, 2H), 3.25–3.17 (m, 4H), 2.63 (brs, 2H), 1.90–1.85 (m, 2H), 1.84–1.77 (m, 4H), 1.50–1.43 (m, 2H), 1.42–1.36 (m, 2H), 1.35–1.26 (m, 4H). ^13^C NMR (150 MHz, CDCl_3_) *δ* 169.5, 155.8, 151.0, 138.9, 134.2, 133.7, 132.4, 132.4, 132.4, 128.0, 125.3, 125.2, 124.7, 120.7, 115.9, 111.0, 53.6, 48.4, 43.2, 39.6, 30.8, 29.3, 28.5, 28.5, 26.5, 24.0, 22.1, 20.8. ESI-MS *m*/*z* 542.1 [M + H]^+^. HR-ESI-MS: [M + H]^+^ calculated for C_28_H_34_BrClN_3_O^+^ 542.1568, found 542.1561.

*2-Phenyl-N-(7-((1,2,3,4-tetrahydroacridin-9-yl)amino)heptyl)acetamide* (**12l**). Yellow oil. Yield: 52.7 mg, 47.2%. ^1^H NMR (600 MHz, CDCl_3_) *δ* 7.93 (d, *J* = 8.5 Hz, 1H), 7.89 (d, *J* = 8.3 Hz, 1H), 7.53 (dd, *J* = 8.4, 7.0 Hz, 1H), 7.34–7.30 (m, 3H), 7.26 (dd, *J* = 8.5, 7.0 Hz, 1H), 7.24–7.21 (m, 2H), 5.48 (brs, 1H, NH), 3.54 (s, 2H), 3.44 (t, *J* = 7.2 Hz, 2H), 3.19–3.14 (m, 2H), 3.05 (brs, 2H), 2.69 (brs, 2H), 1.94–1.87 (m, 4H), 1.64–1.57 (m, 2H), 1.40–1.35 (m, 2H), 1.34–1.29 (m, 2H), 1.29–1.25 (m, 2H), 1.23–1.17 (m, 2H). ^13^C NMR (150 MHz, CDCl_3_) *δ* 171.0, 158.6, 150.8, 147.6, 135.1, 129.5, 129.1, 128.8, 128.3, 127.4, 123.7, 122.9, 120.3, 116.0, 49.5, 44.0, 39.6, 34.2, 31.8, 29.5, 29.0, 26.9, 26.7, 24.9, 23.1, 22.9. ESI-MS *m*/*z* 430.2 [M + H]^+^. HR-ESI-MS: [M + H]^+^ calculated for C_28_H_36_N_3_O^+^ 430.2853, found 430.2847.

### 3.3. AChE/BChE Inhibitory Assay

The AChE and BChE inhibitory activities of compounds were determined by using a slightly modified Ellman’s method [30,31]. Electric eel AChE, equine serum BChE, 5,5′-dithiobis(2-nitrobenzoic acid) (DTNB), phosphate buffer solution (PBS, pH 8.0), acetylthiocholine (ATC) iodide, and butyrylthiocholine (BUC) iodide were purchased from Sigma-Aldrich (Steinheim, Germany). Tacrine was used as positive control. Enzyme solutions were prepared at 2.0 U/mL in 2-mL aliquots. The assay medium consisted of 10 μL of enzyme, 40 μL of PBS, 20 μL of 0.01 M DTNB, and 10 μL of tested compound. Assayed solutions of tested compounds were pre-incubated with corresponding ChE for 5 min. The reaction was initiated by the addition of 20 μL of 0.01 M substrate (ATC or BUC). The activity was determined by measuring the increase in absorbance at 410 nm at 37 °C in 2-min intervals using Tecan Spark multimode microplate reader (Mannedorf, Switzerland). The percentage of inhibition (*I*) was calculated from the measured data as follows: *I* = (A_c_ − A_i_)/A_c_ × 100%, where A_i_ and A_c_ represent the change in the absorbance in the presence of an inhibitor and without an inhibitor, respectively.

### 3.4. Kinetic Assay

Kinetic studies of inhibition on AChE and BChE were performed by using Ellman’s method as described above. The concentrations of used substrates were 0.07813 µM, 0.1563 µM, 0.3125 µM, and 0.625 µM. Linear regression was used for calculation of Lineweaver−Burk plots, and all of the calculations were performed using GraphPad Prism 5.0 software (GraphPad Software, La Jolla, CA, USA).

### 3.5. Molecular Docking

Molecular docking studies were performed using the Autodock 4.2 program (The Scripps Research Institute, La Jolla, CA, USA) [32,33]. The crystal structure of AChE (PDB ID: 5EI5) was obtained from the Protein Data Bank after eliminating the inhibitor and water molecules. The 3D structure of the ligand was built, and performed MMFF94 minimization by using ChemBio3D Ultra 12.0 (CambridgeSoft Corporation, Cambridge, MA, USA). Using Autodock Tools 1.5.6, the preparation of the receptor was made by the addition of hydrogen atoms and Gasteiger charges, and finally an assignment of atomic types as AD4 type, and then autotorsion was used to define the rotatable bonds in the ligand preparation. The resulting enzyme structure was used as an input for the Autogrid program. Autogrid performed pre-calculated atomic affinity grid maps for each atom type in the ligand, plus an electrostatics map and a separate de-solvation map presented in the substrate molecule. The dimensions of the box were set to 60 × 60 × 60 with grid spacing of 0.375 Å. Rigid ligand docking was performed for the compounds. Docking calculations were carried out using the Lamarckian genetic algorithm (LGA). The proposed docking complex image was created by Pymol 1.5 (DeLano Scientific LLC, San Carlos, California, USA).

### 3.6. Determination of the Inhibitory Potency on Self-Aβ_1–42_ Aggregation

In order to investigate the inhibition of compounds on Aβ_1–42_ self-aggregation, a ThT-based fluorometric assay was performed [34]. 1,1,1,3,3,3-hexafluoro-2-propanol (HFIP) pre-treated Aβ_1–42_ (GL Biochem Ltd., Shanghai, China) was dissolved in DMSO to make a 200 µM stock solution. The stock solution was centrifuged at the speed of 13,500 rpm for 10 min. The above supernatant was used for experiments. The tested compounds were dissolved in DMSO at concentrations of 0.8 mM. A screening assay for the tested compounds to inhibit A aggregation was performed by measuring ThT fluorescence emission. Compounds (2 µL) and 2 µL of 200 µM Aβ_1–42_ were added into 76 µL of phosphate-buffered saline (PBS at pH 7.4) in a 96-well plate. After incubation for 24 h at room temperature, 80 µL of 5 µM of ThT solution (in 50 mM of glycine-NaOH at pH 8.5) was added to the reaction solution. Fluorescence emission was measured at 490 nm with an excitation wavelength of 450 nm on a Tecan Spark multimode microplate reader. Identical spectra were recorded by performing the independent experiments thrice. The fluorescence intensities were compared, and the % inhibition was calculated by the following equation: 100 − [(F_i_ − F_b_)/(F_o_ − F_b_) × 100], where F_i_, F_o_ and F_b_ are the fluorescence intensities obtained for Aβ aggregation in the presence of inhibitors Aβ_1–42_ and ThT; in the presence of Aβ_1–42_ and THT but no inhibitors; and the blanks containing ThT only.

### 3.7. Determination of the Inhibitory Potency on Aβ_1–42_ Aggregation Induced by AChE

The co-incubation experiment of Aβ_1–42_ with AChE was performed by ThT bioassay according to a reported protocol [35]. HFIP pre-treated Aβ_1–42_ (GL Biochem Ltd., shanghai, China) was dissolved in DMSO to make a 200 µM stock solution. The stock solution was centrifuged at the speed of 13,500 rpm for 10 min. The above supernatant was used for experiments. The tested compounds were dissolved in DMSO at concentrations of 10 mM, and diluted to 640 µM by phosphate-buffered saline (PBS at pH 7.4). A screening assay for tested compounds to inhibit A aggregation was performed by measuring ThT fluorescence emission. Compounds (2 µL) and 2 µL of 200 µM Aβ_1–42_ and 20 µL AchE (2 μ/mL, in PBS at pH 8.0) were added into 76 µL of PBS (pH 8.0) in a 96-well plate. After incubation for 24 h at room temperature, 100 µL of 5 µM ThT solution (in 50 mM of glycine-NaOH at pH 8.5) was added to the reaction solution. Fluorescence emission was measured at 490 nm with an excitation wavelength of 450 nm on a Tecan Spark multimode microplate reader. Identical spectra were recorded by performing the independent experiments thrice. The fluorescence intensities were compared, and the % inhibition was calculated by the following equation: 100 − [(F_i_ − F_b_)/(F_o_ − F_b_) × 100], where F_i_, F_o_ and F_b_ are the fluorescence intensities obtained for Aβ aggregation in the presence of inhibitors Aβ_1–42_, AChE, and ThT; in the presence of Aβ_1–42_, AChE, and ThT but no inhibitors; and the blanks containing ThT only.

### 3.8. Cytotoxicity Bioassay

The cytotoxicity of selected compounds was evaluated on HepG2 cells. Cells were inoculated into 96-well plates. After incubation for 24 h, the cells were treated with different concentrations of tested compounds for 24 h, and then were incubated with 10 μL of 3-(4,5-dimethylthiazol-2-yl)-2,5-diphenyltetrazolium bromide (MTT) at 37 °C for 2 h. The formazan dye product was measured by the absorbance at 490 nm on a Tecan Spark multimode microplate reader.

## 4. Conclusions

In summary, pulmonarin B (**1**) and a series of brominated phenylacetic acid/tacrine hybrids **10a**–**10h** and **12a**–**12l** were synthesized and evaluated for their anti-AD potential. Compound **1** was found to be a moderate dual AChE/BChE inhibitor and also showed inhibition on self-induced and AChE-induced Aβ aggregation, which established **1** as an interesting marine hit for further anti-AD study. Among these hybrids, the best result was obtained for compound **12j** with IC_50_ of 0.182 ± 0.006 μM for AChE and 0.064 ± 0.006 μM for BChE. The kinetic and molecular docking studies well confirmed **12j** as a mixed-type AChE inhibitor. Moreover, **12j** displayed the highest inhibitory activity against self-induced and AChE-induced Aβ aggregation. In addition, compound **12j** did not show obvious hepatotoxicity in comparison with tacrine and donepezil. In the present study, the biological evaluation together with computational analyses demonstrated these newly designed brominated phenylacetic acid/tacrine hybrids as attractive lead compounds toward the discovery of multifunctional anti-AD drugs.

## Figures and Tables

**Table 1:** In vitro inhibition of AChE and butyrylcholinesterase (BChE) presented as IC_50_ (μM) ^1^.

Compound	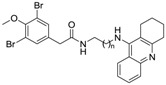	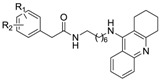	*Ee*AChE ^2^	eqBChE ^3^
**1**			37.02 ± 2.11	30.70 ± 1.44
**10a**	1		0.880 ± 0.074	0.230 ± 0.010
**10b**	2		0.771 ± 0.027	0.233 ± 0.013
**10c**	3		1.045 ± 0.161	0.195 ± 0.011
**10d**	4		0.764 ± 0.016	0.100 ± 0.014
**10e**	5		0.686 ± 0.045	0.054 ± 0.006
**10f**	6		0.314 ± 0.010	0.053 ± 0.007
**10g**	7		0.427 ± 0.016	0.325 ± 0.004
**10h**	9		0.638 ± 0.026	0.516 ± 0.037
**12a**		R_1_ = 2-Br, R_2_ = 4-OMe	0.324 ± 0.033	0.140 ± 0.004
**12b**		R_1_ = H, R_2_ = 4-OMe	0.750 ± 0.054	0.694 ± 0.003
**12c**		R_1_ = 3-Br, R_2_ = 4-OMe	0.607 ± 0.045	0.152 ± 0.006
**12d**		R_1_ = 2-Br, R_2_ = H	0.304 ± 0.004	0.091 ± 0.017
**12e**		R_1_ = 3-Br, R_2_ = H	0.724 ± 0.003	0.149 ± 0.021
**12f**		R_1_ = 4-Br, R_2_ = H	0.597 ± 0.028	0.214 ± 0.004
**12g**		R_1_ = 2-Br, R_2_ = 5-Br	0.696 ± 0.052	0.062 ± 0.006
**12h**		R_1_ = 3-Br, R_2_ = 5-Br	0.729 ± 0.021	0.204 ± 0.011
**12i**		R_1_ = 3-Br, R_2_ = 4-F	0.748 ± 0.017	0.049 ± 0.004
**12j**		R_1_ = 5-Br, R_2_ = 2-F	0.182 ± 0.006	0.064 ± 0.006
**12k**		R_1_ = 2-Br, R_2_ = 5-Cl	0.383 ± 0.054	0.104 ± 0.001
**12l**		R_1_ = R_2_ = H	0.760 ± 0.092	0.680 ± 0.054
Tacrine			0.159 ± 0.007	0.046 ± 0.002

^1^ Results were expressed as mean ± SD from three separate experiments. ^2^ *Ee*AChE: AChE from electric eel. ^3^ eqBChE: BChE from equine serum.

**Table 2:** Inhibition of self-induced and AChE-induced Aβ aggregation by compounds **1** and **12j** in comparison with tacrine, donepezil, and galantamine.

Compounds	Inhibition of Aβ Aggregation (%)	
	Self-Induced ^1^	AChE-Induced ^2^
**1**	29.78 ± 1.45	27.60 ± 1.96
**12j**	32.37 ± 0.62	47.73 ± 4.35
**Tacrine**	NT ^3^	21.84 ± 1.60
**Donepezil**	17.95 ± 0.77	22.42 ± 2.56
**Galantamine**	1.25 ± 0.46	3.58 ± 1.42

^1^ Inhibition of self-induced Aβ_1–42_ aggregation (2.5 µM) produced by the tested compounds at 10 µM concentration. Values are expressed as the mean ± SD of three separate experiments. ^2^ Co-aggregation inhibition of Aβ_1–42_ aggregation (2.5 µM) and AChE (0.2 U/mL) by the tested compounds at 8 µM concentration. Values are expressed as the mean ± SD of three separate experiments. ^3^ Not tested.

**Table 3:** Results of in vitro cytotoxicity toward human hepatoma cell line (HepG2) cells.

Compound	IC_50_^1^
**1**	>80 ^2^
**12j**	>80
Tacrine	38.87 ± 0.53
Donepezil	37.92 ± 1.46

^1^ Values are expressed as the mean ± SD of three separate experiments. ^2^ Cell viability >50% of compounds at 80 µM.

## Supplementary Materials

The following are available online at http://www.mdpi.com/1660-3397/16/9/293/s1, Supplementary materials including ^1^H, ^13^C NMR and HR-ESIMS spectra for compounds **1**, **10a**–**10h** and **12a**–**12l**.
