# Supplementary material for: Total Synthesis of Pulmonarin B and Design of Brominated Phenylacetic Acid/Tacrine Hybrids: Marine Pharmacophore Inspired Discovery of New ChE and Aβ Aggregation Inhibitors
Source: Mar Drugs. 2018 Aug 21;16(9):293. doi: 10.3390/md16090293 (PMC6164518; doi:10.3390/md16090293)

Supporting Information

Total synthesis of pulmonarin B and design of bromated-phenylacetic acid/tacrine hybrids: marine pharmacophore inspired discovery of new ChE and Aβ aggregation inhibitors

Zhi-Qiang Cheng [1], Jia-Li Song [1], Kongkai Zhu [1], Juan Zhang [2], Cheng-Shi Jiang [1,*], and Hua Zhang [1,*]

[1] School of Biological Science and Technology, University of Jinan, Jinan 250022, China;

[2] School of Biological Sciences, University of Brasília, Brasília 72220275, Brazil.

* Correspondence: jiangchengshi-20@163.com; bio_zhangh@ujn.edu.cn (H.Z.)

Contents:

1. Figure S1. Comparison of the re-docked alkylene-linked bis-tacrine to 5EI5 with its original crystal structures.
2. ^{1}H, ^{13}C NMR and HR-MS (ESI) spectra of pulmonarin B (**1**), **10a–10h** and **12a–12l**.

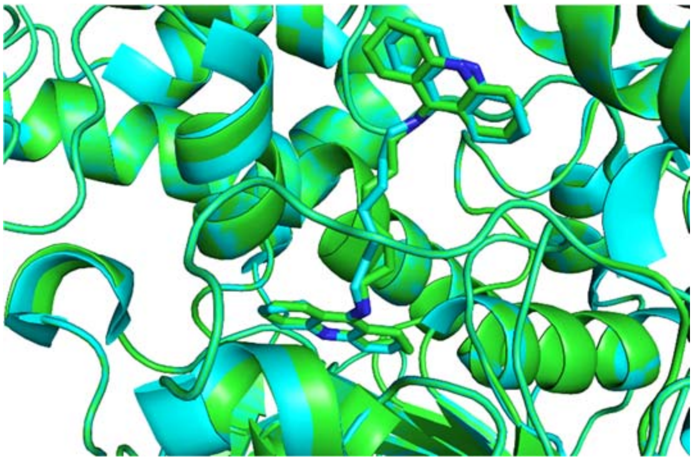

Figure S1. Comparison of the redocked alkylene-linked bis-tacrine to 5EI5 with its original crystal structures. The re-docked ligand was shown in sticks with green color, while the original crystal structure was shown in sticks with cyan color.

^{1}H NMR spectrum of pulmonarin B (**1**)

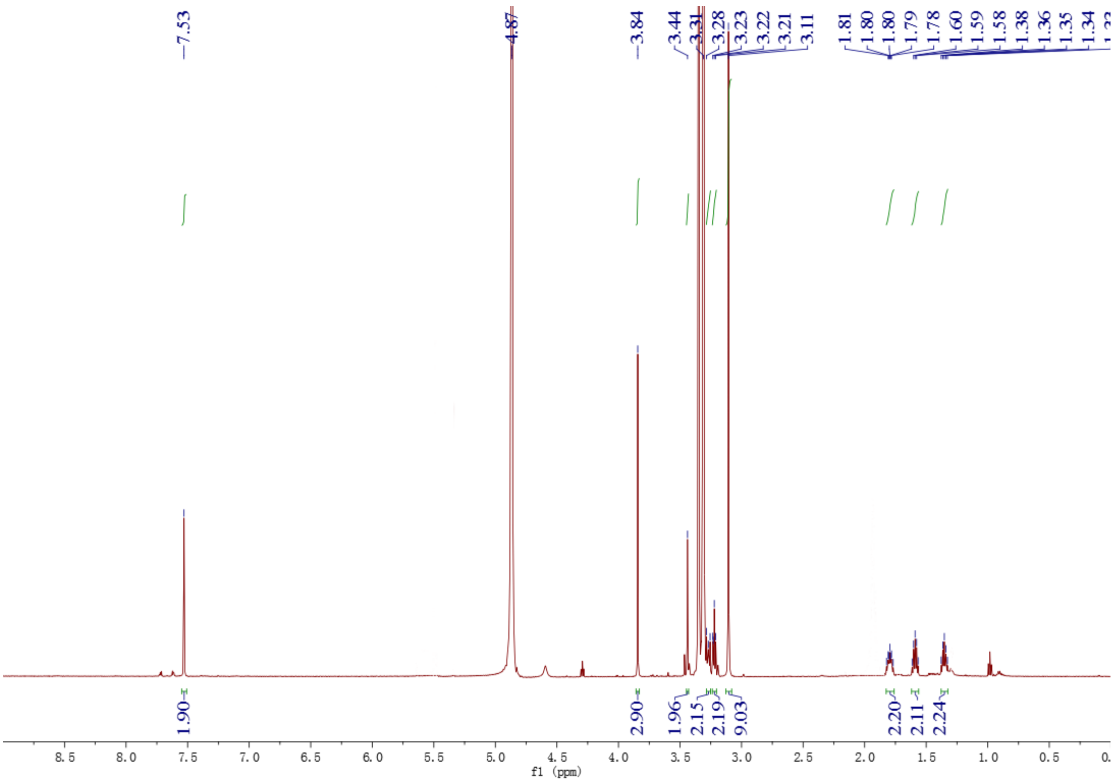

^{13}C NMR spectrum of pulmonarin B (**1**)

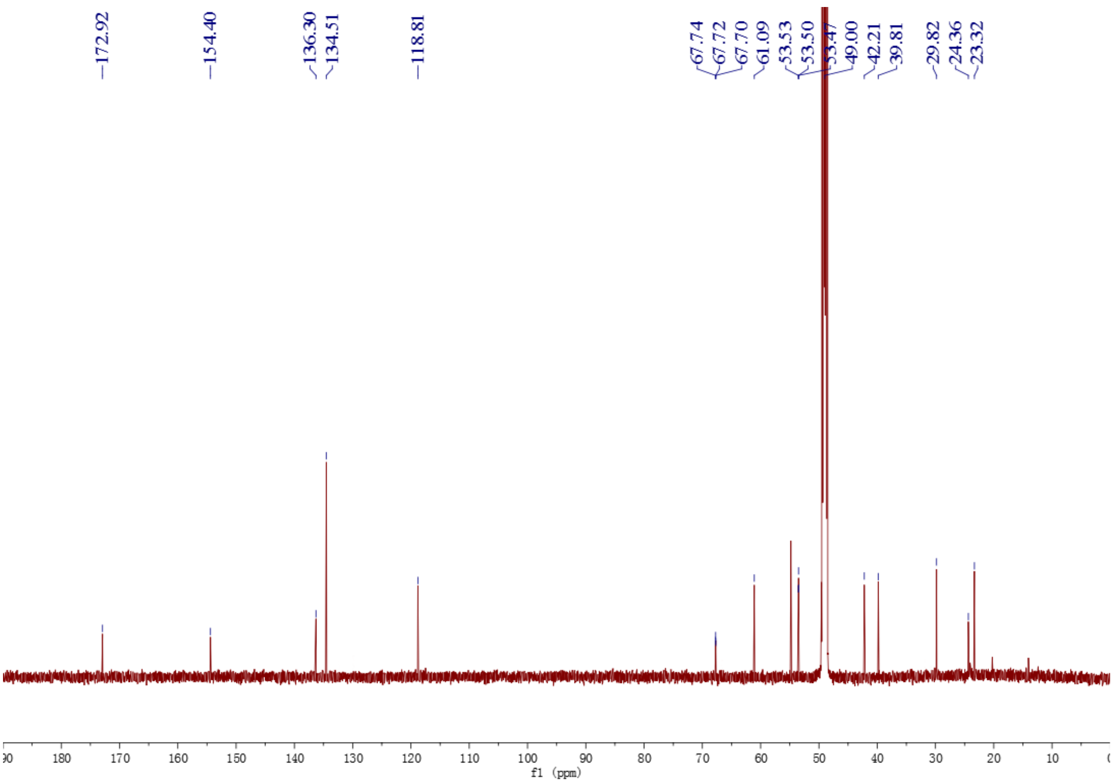

HR-MS (ESI) spectrum of pulmonarin B (**1**)

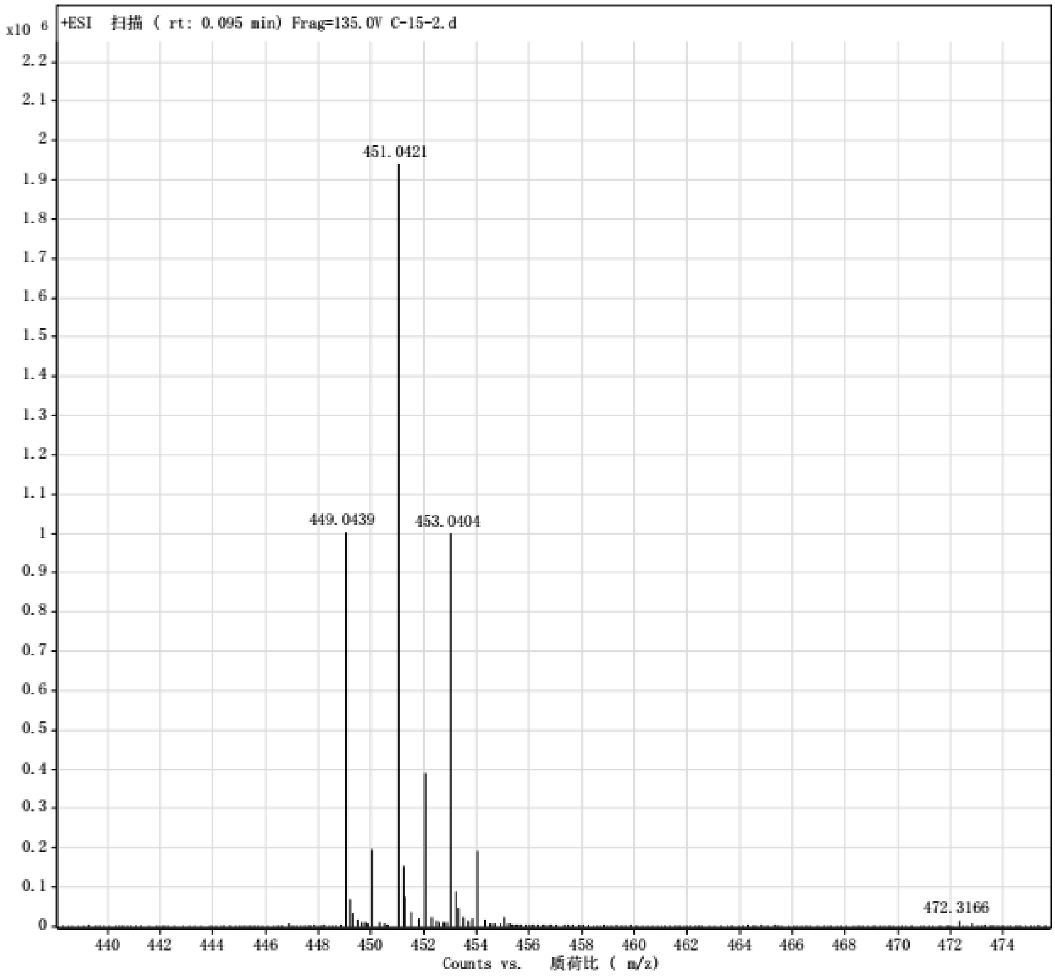

^{1}H NMR spectrum of **5a**

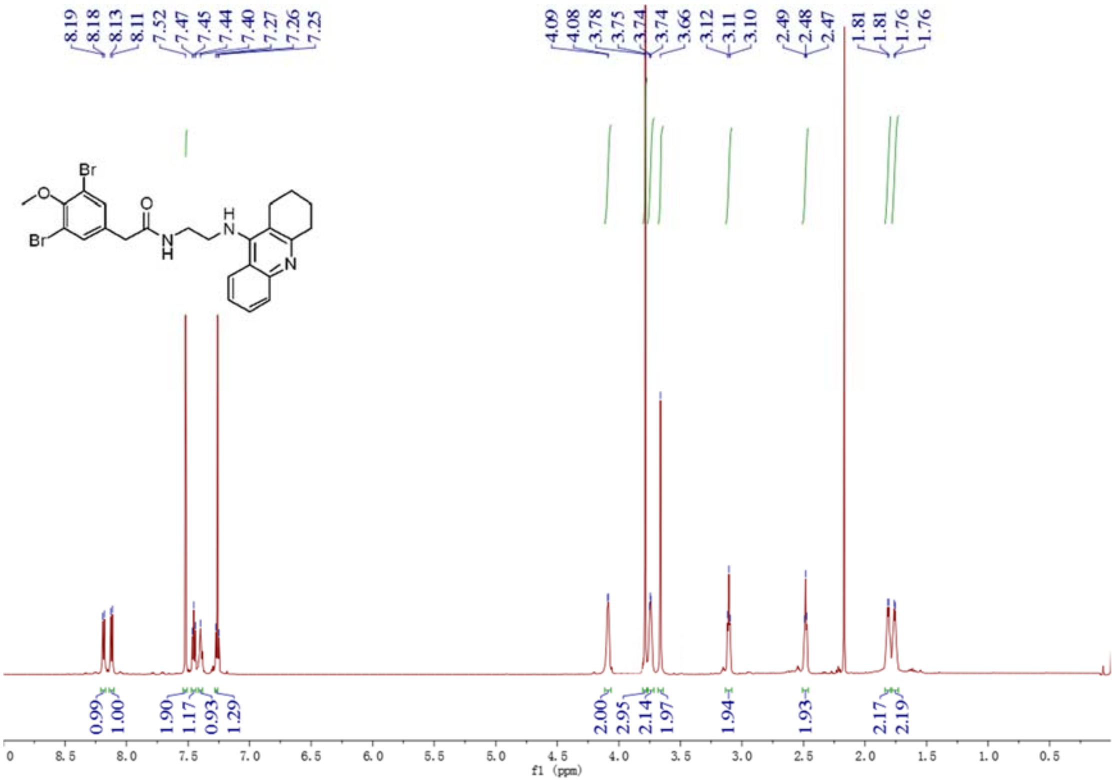

^{13}C NMR spectrum of **5a**

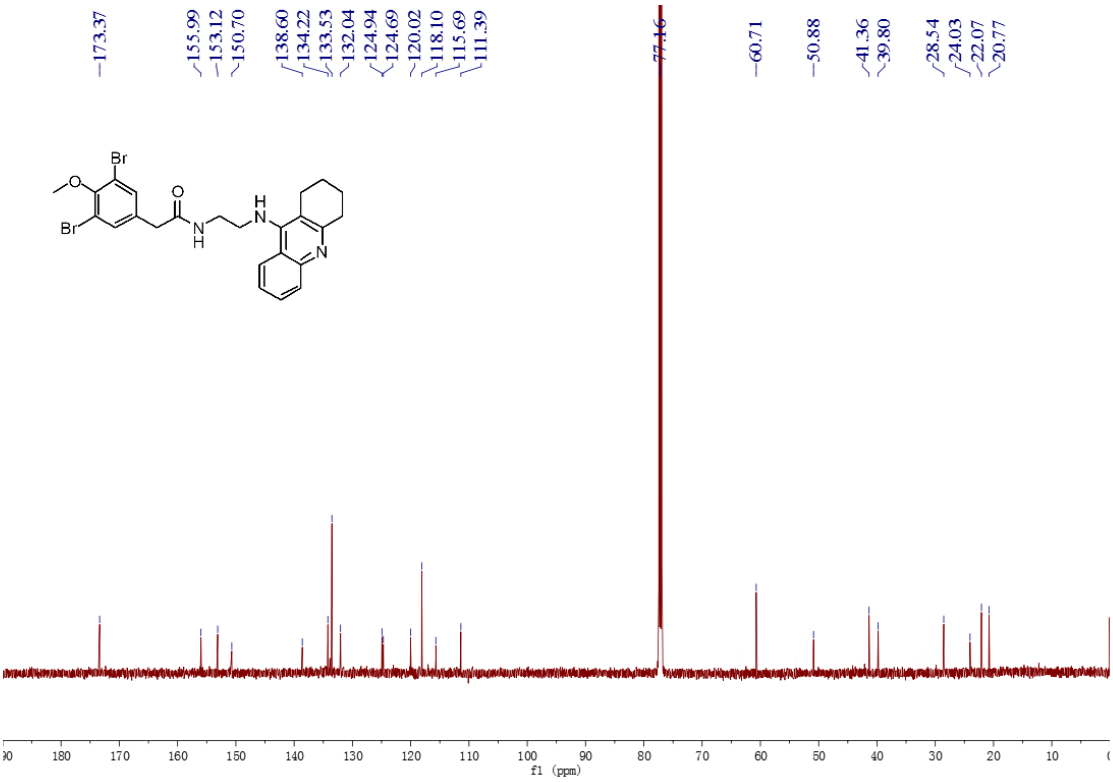

HR-MS (ESI) spectrum of **5a**

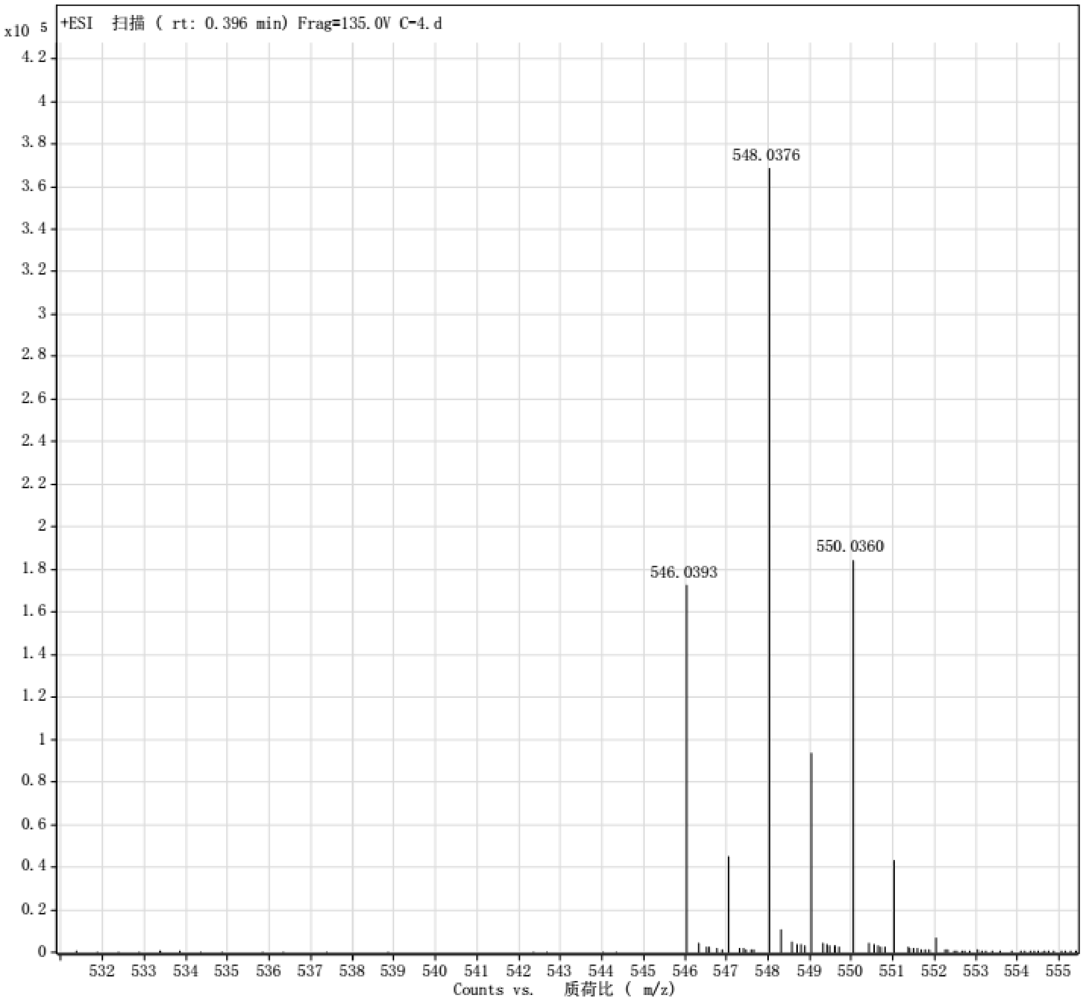

^{1}H NMR spectrum of **5b**

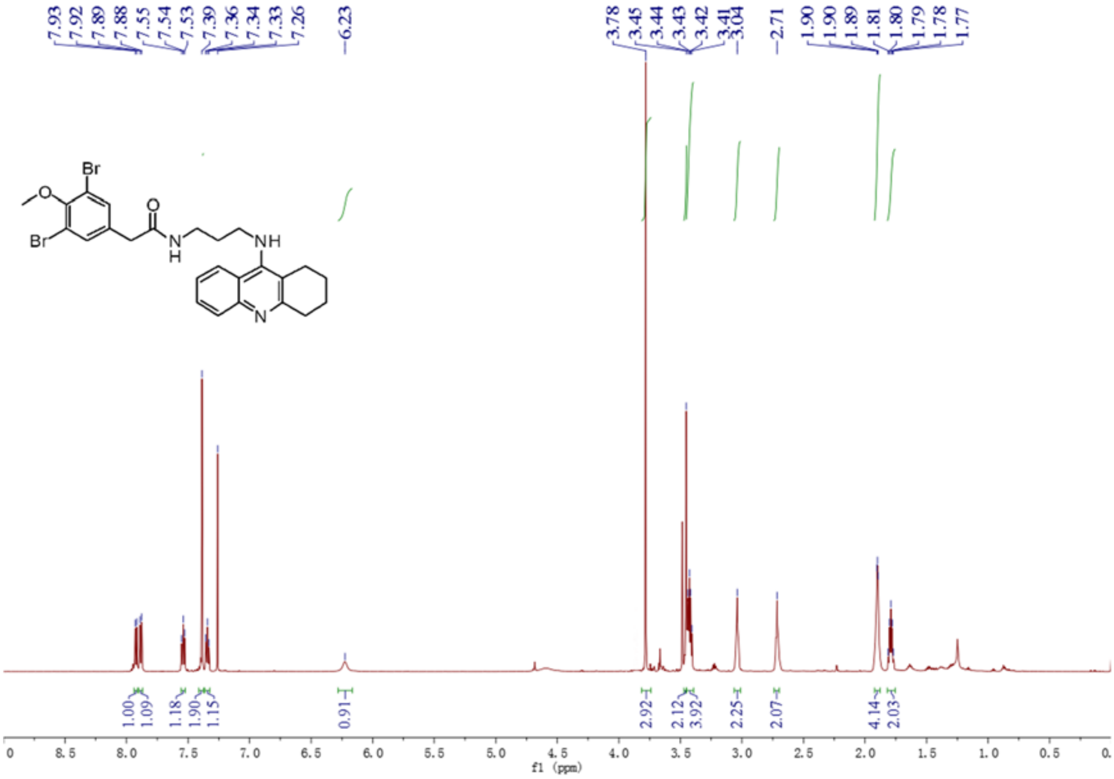

^{13}C NMR spectrum of **5b**

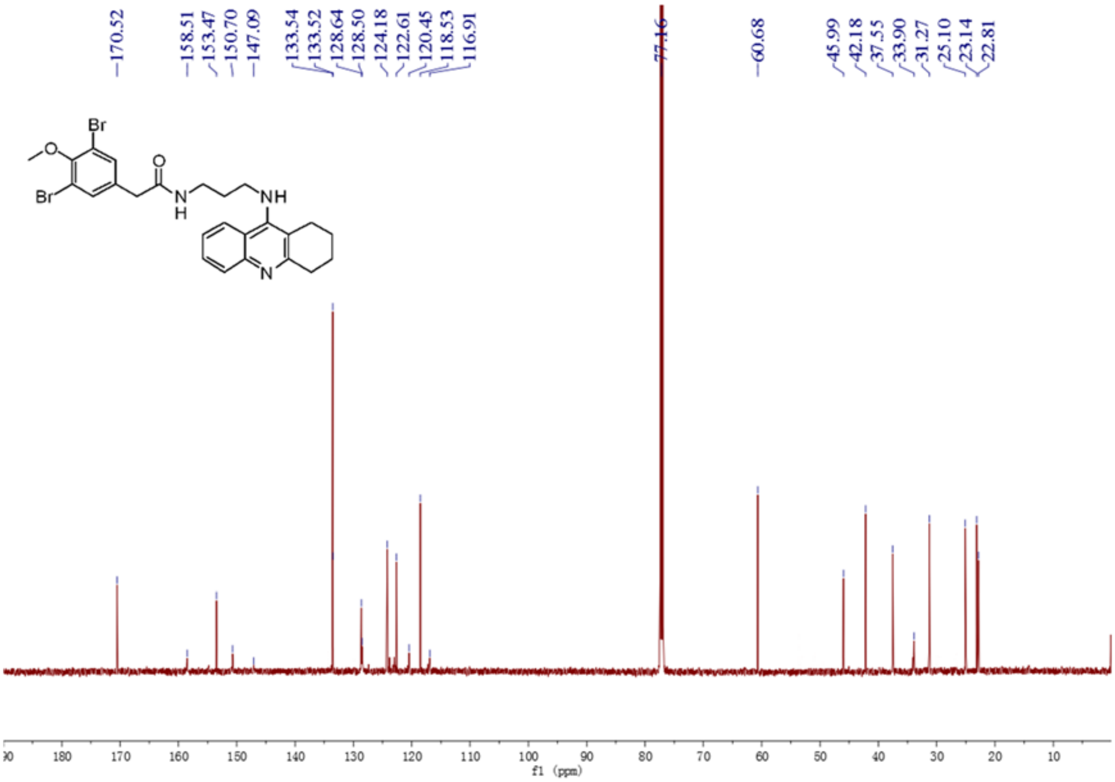

HR-MS (ESI) spectrum of **5b**

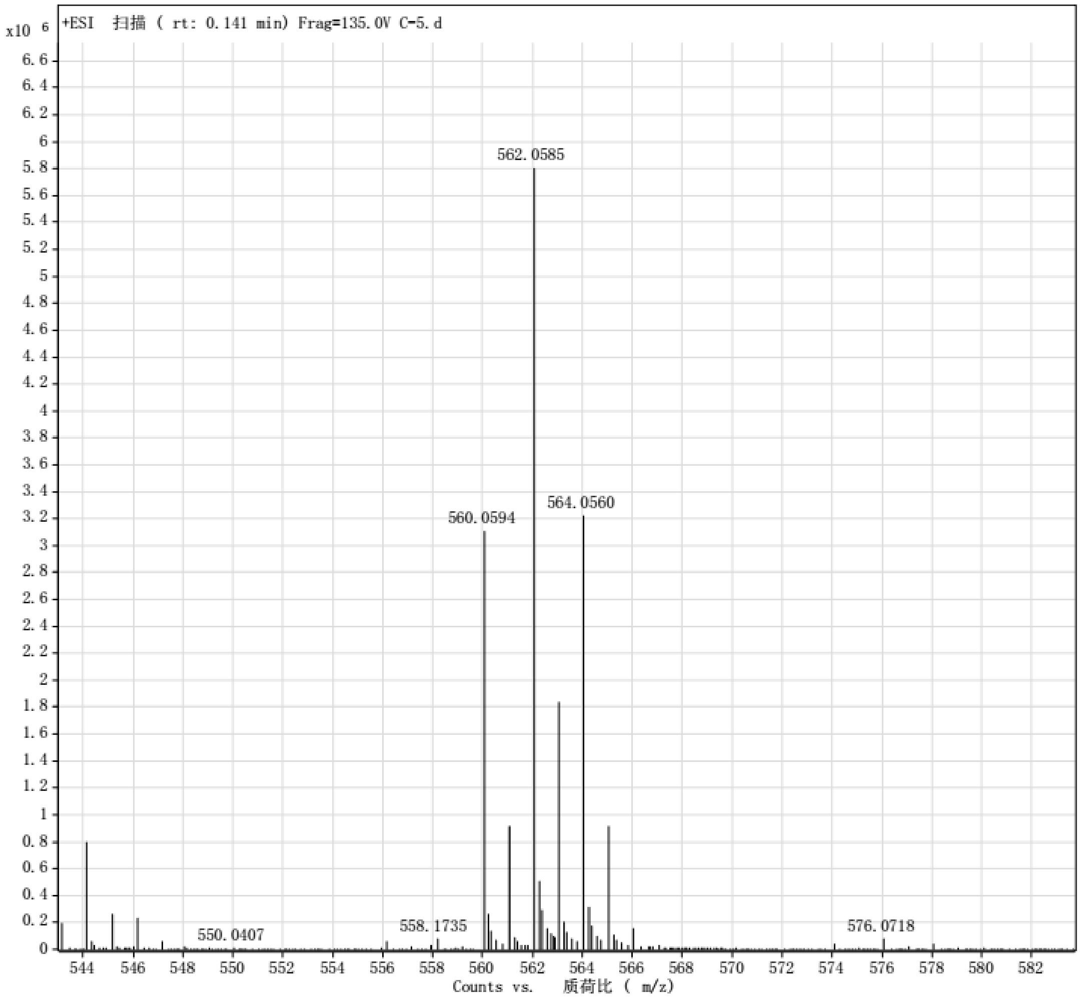

^{1}H NMR spectrum of **5c**

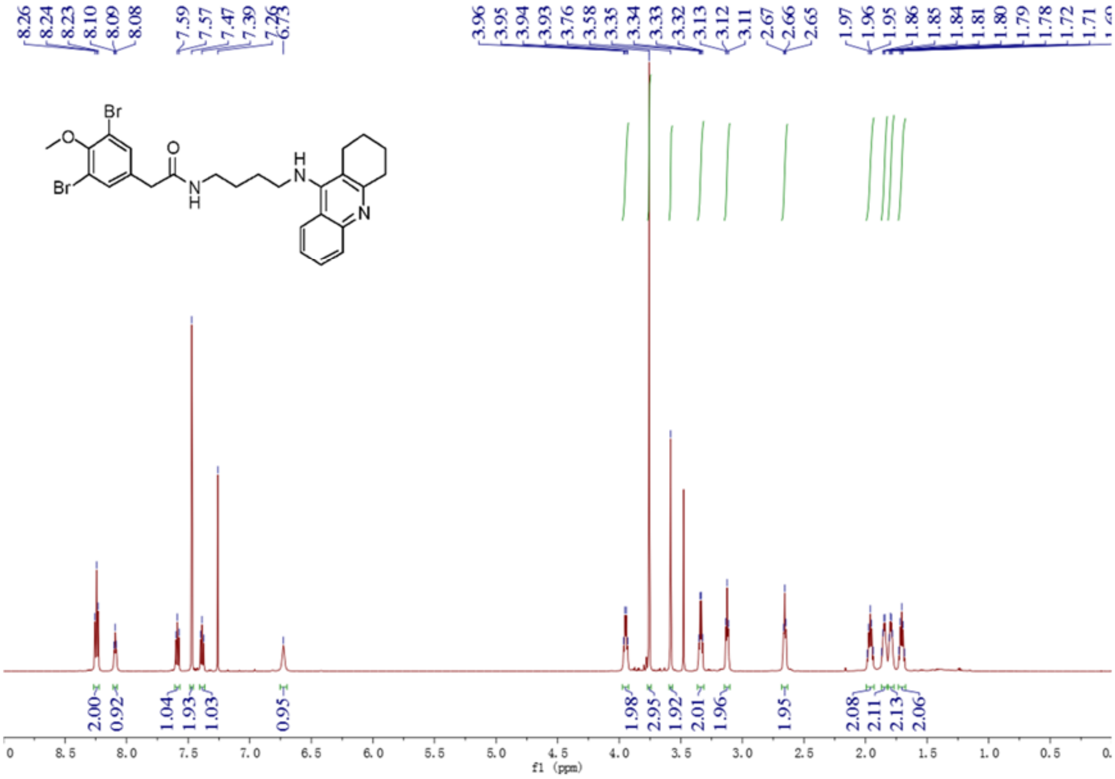

^{13}C NMR spectrum of **5c**

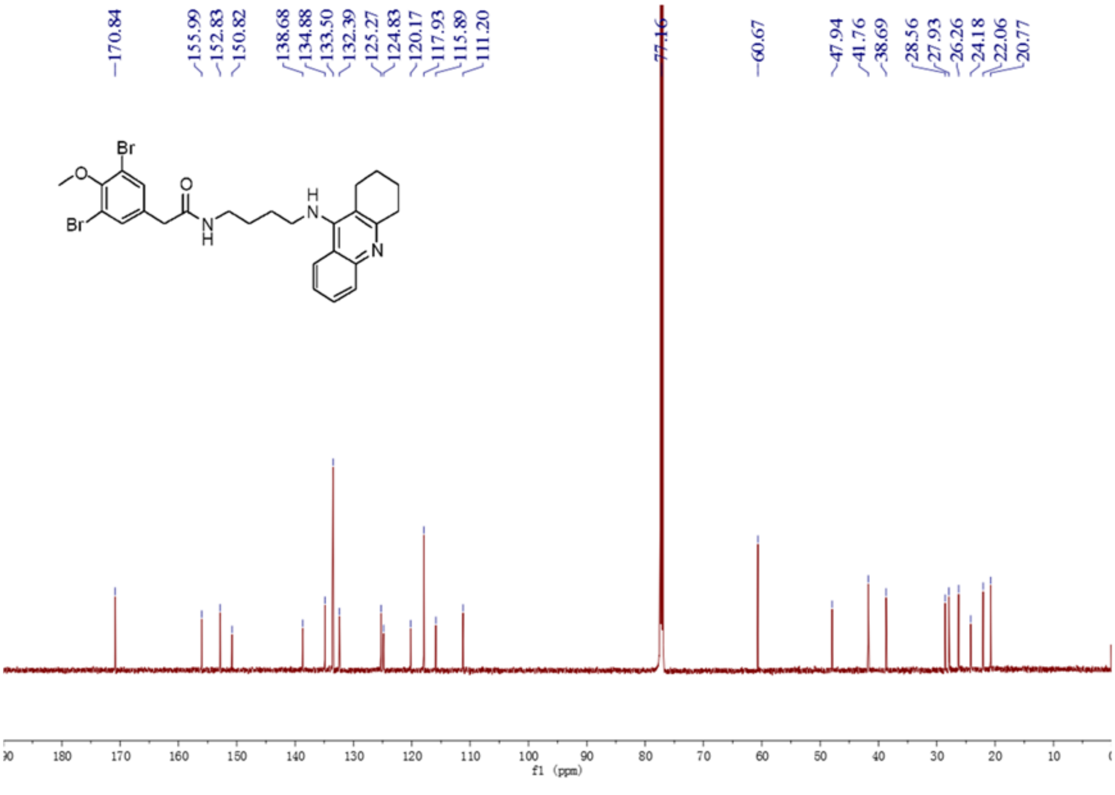

HR-MS (ESI) spectrum of **5c**

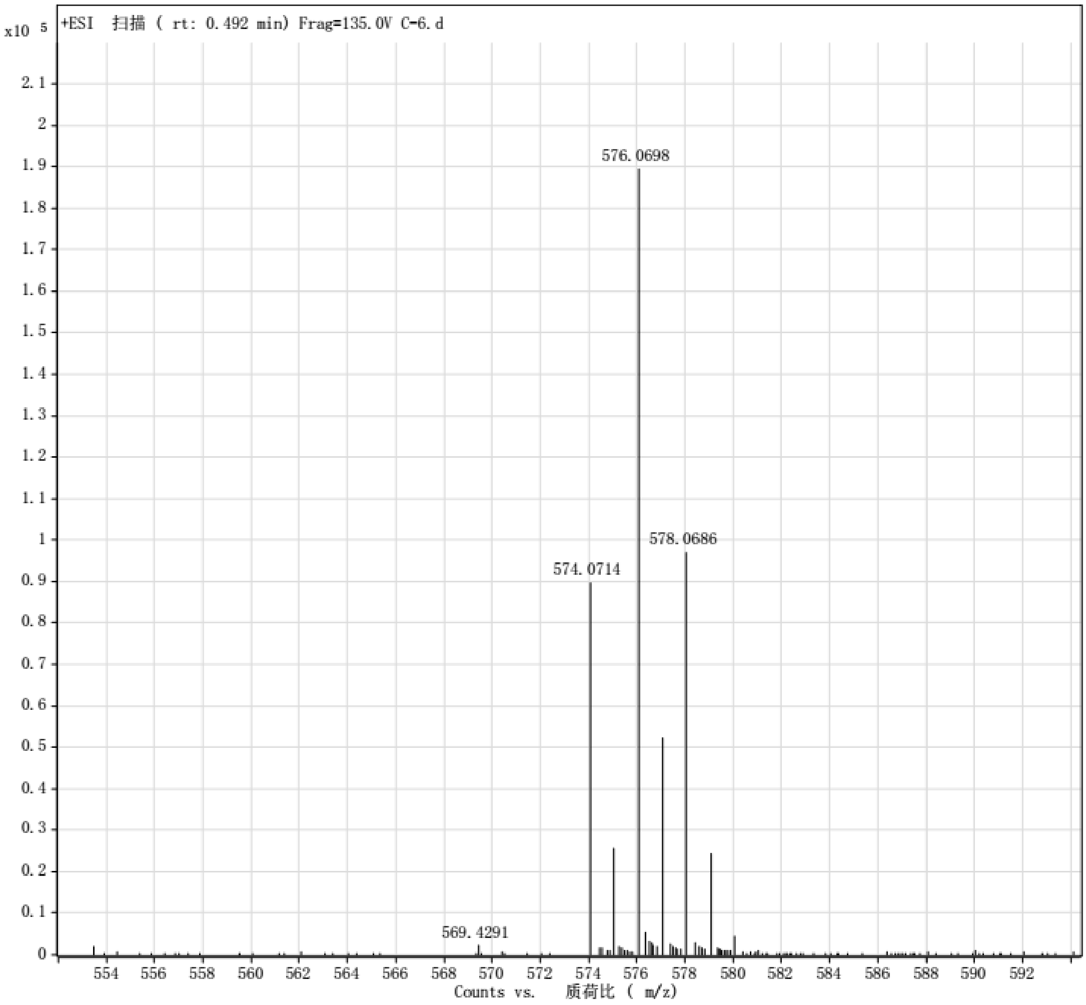

^{1}H NMR spectrum of **5d**

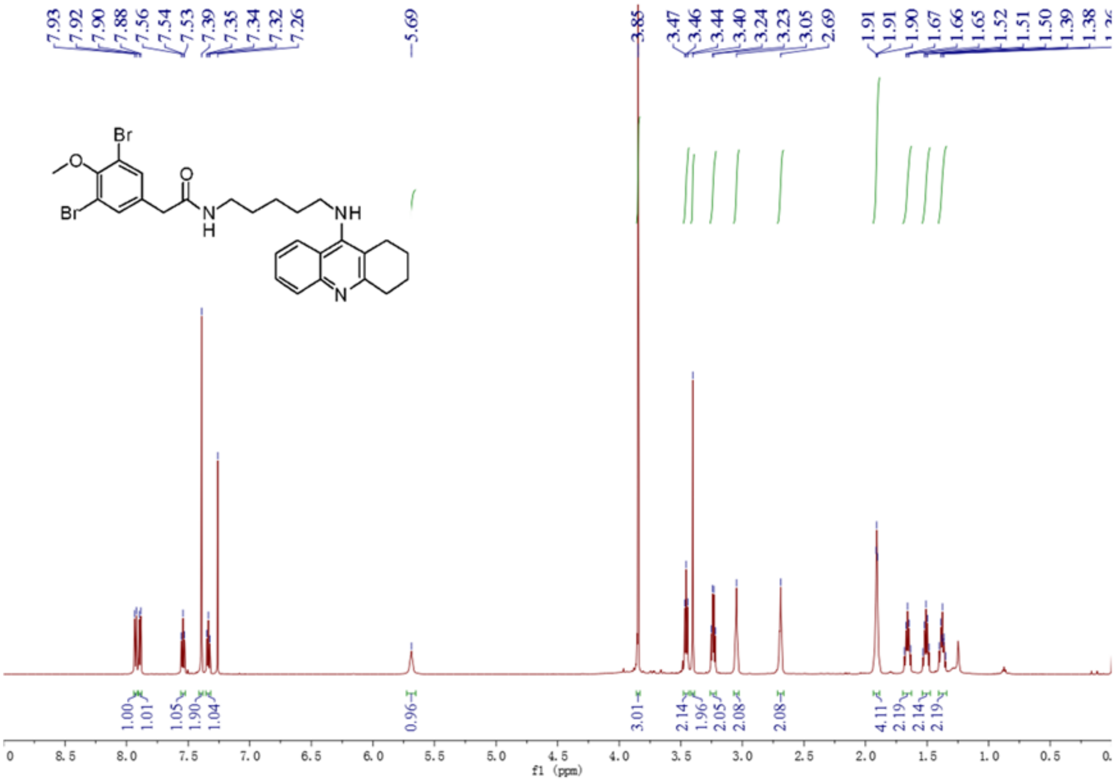

^{13}C NMR spectrum of **5d**

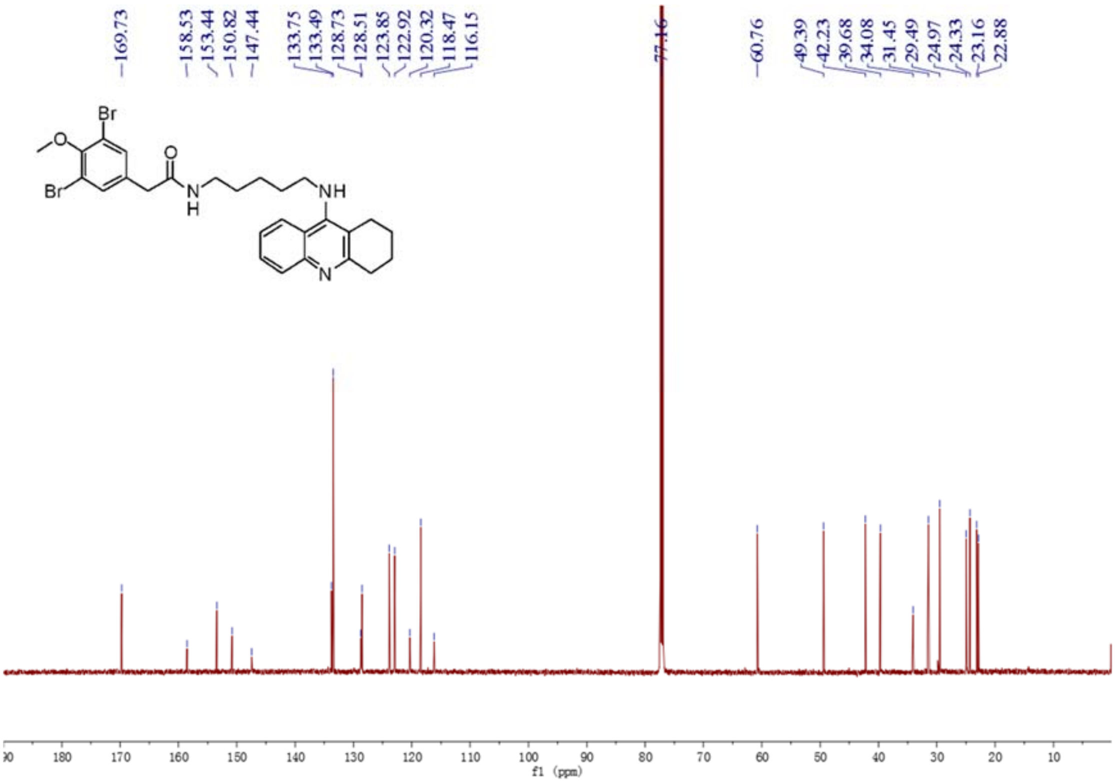

HR-MS (ESI) spectrum of **5d**

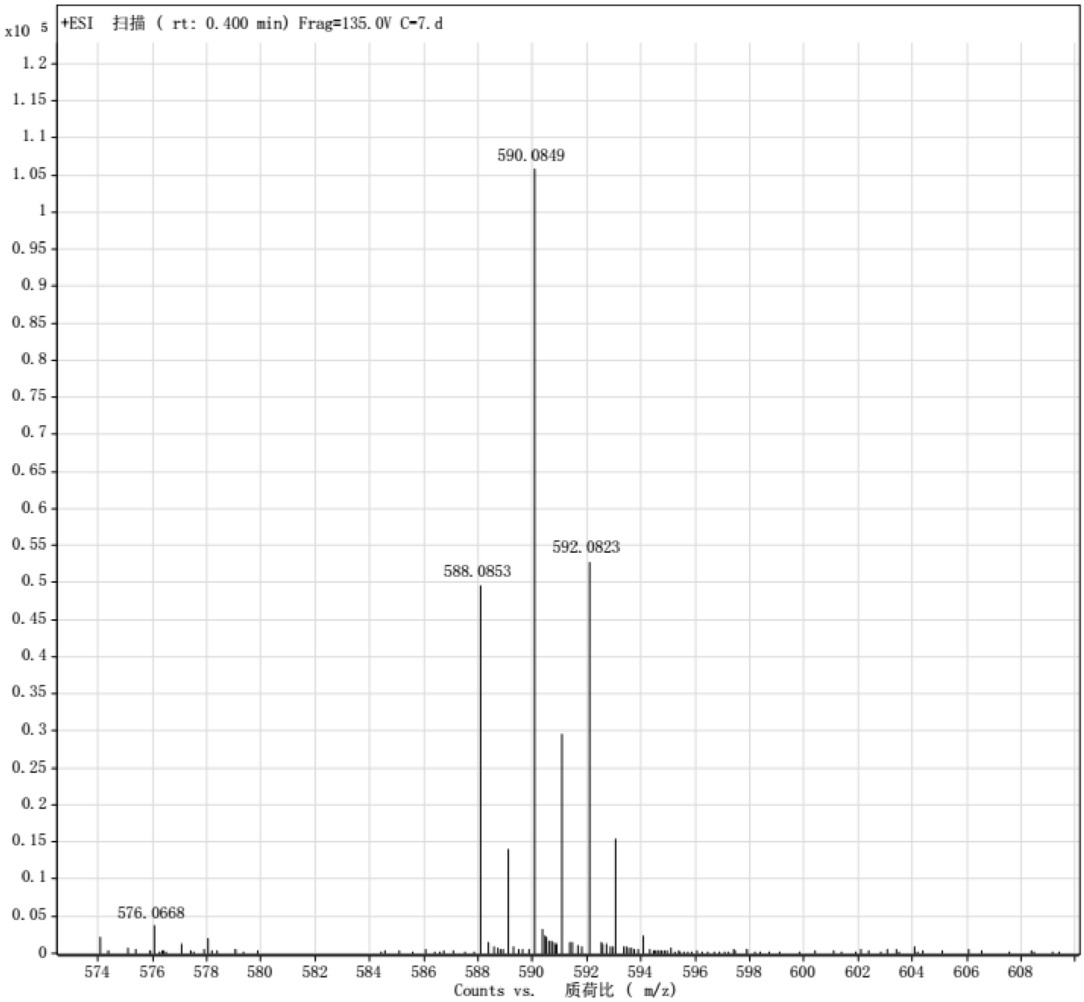

^{1}H NMR spectrum of **5e**

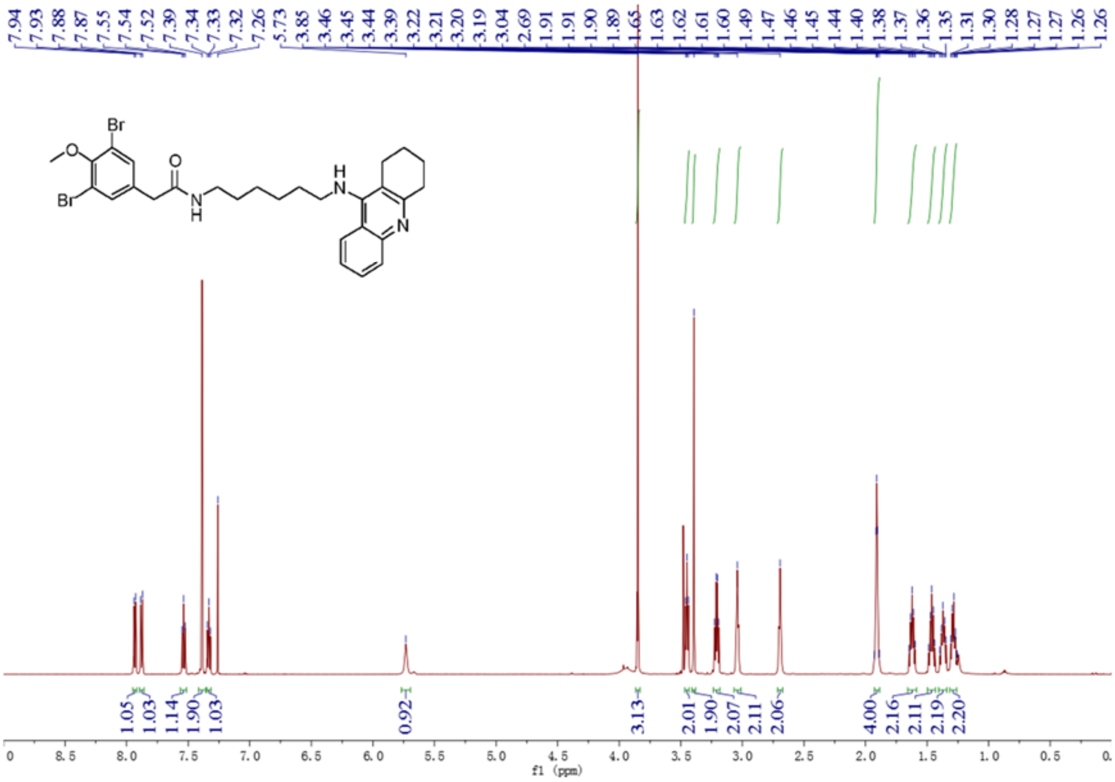

^{13}C NMR spectrum of **5e**

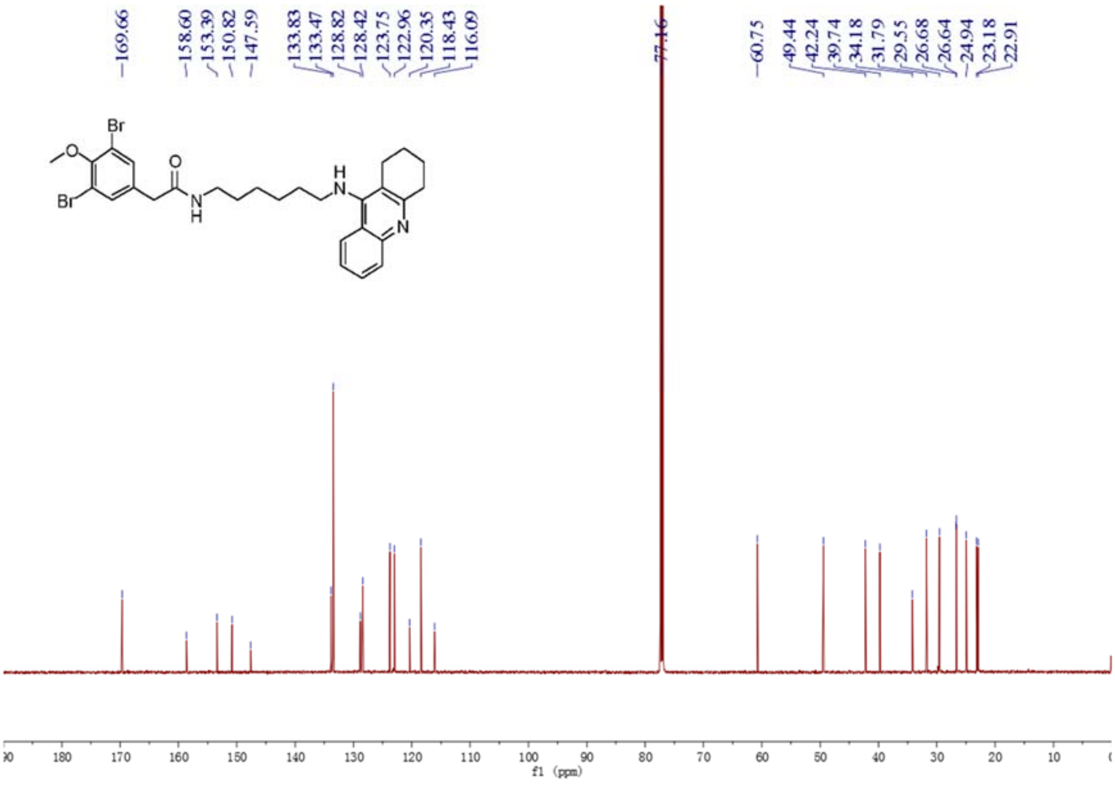

HR-MS (ESI) spectrum of **5e**

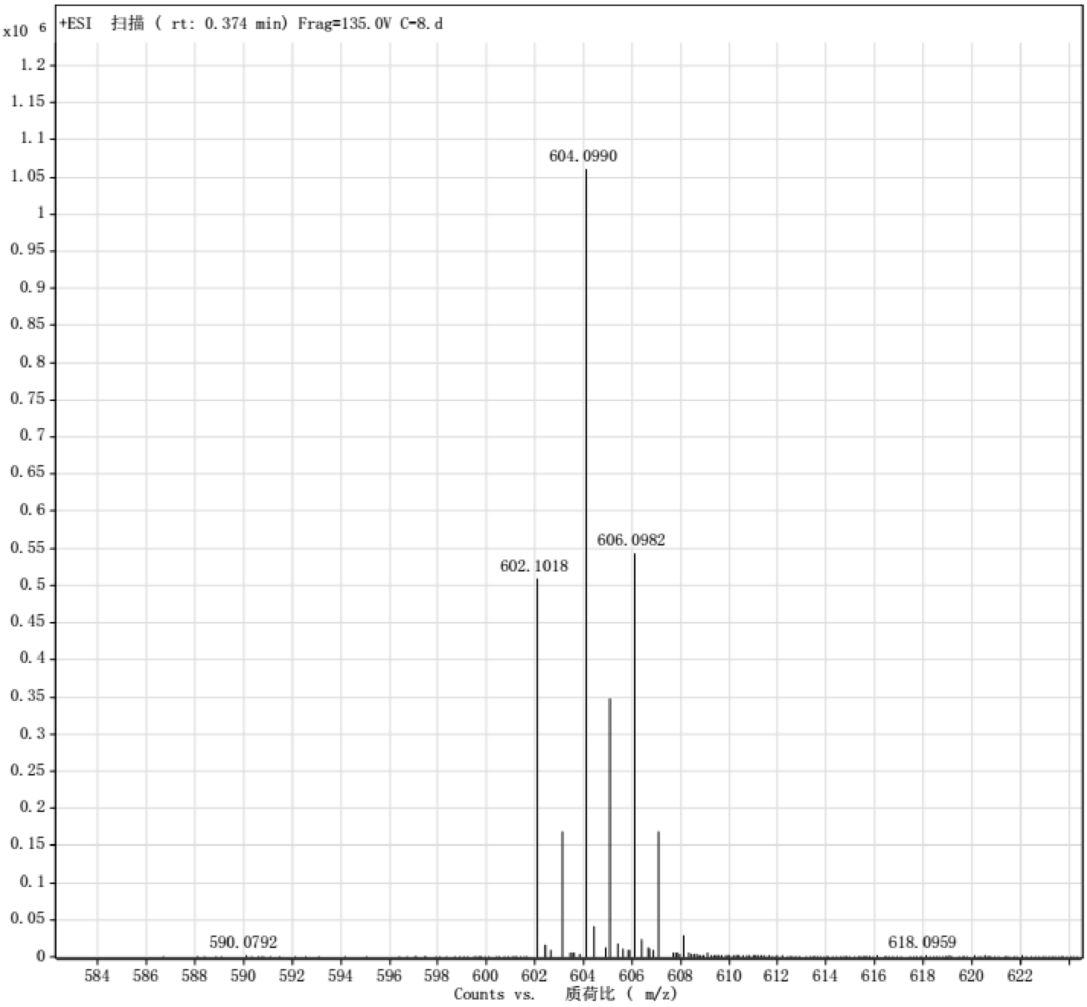

^{1}H NMR spectrum of **5f**

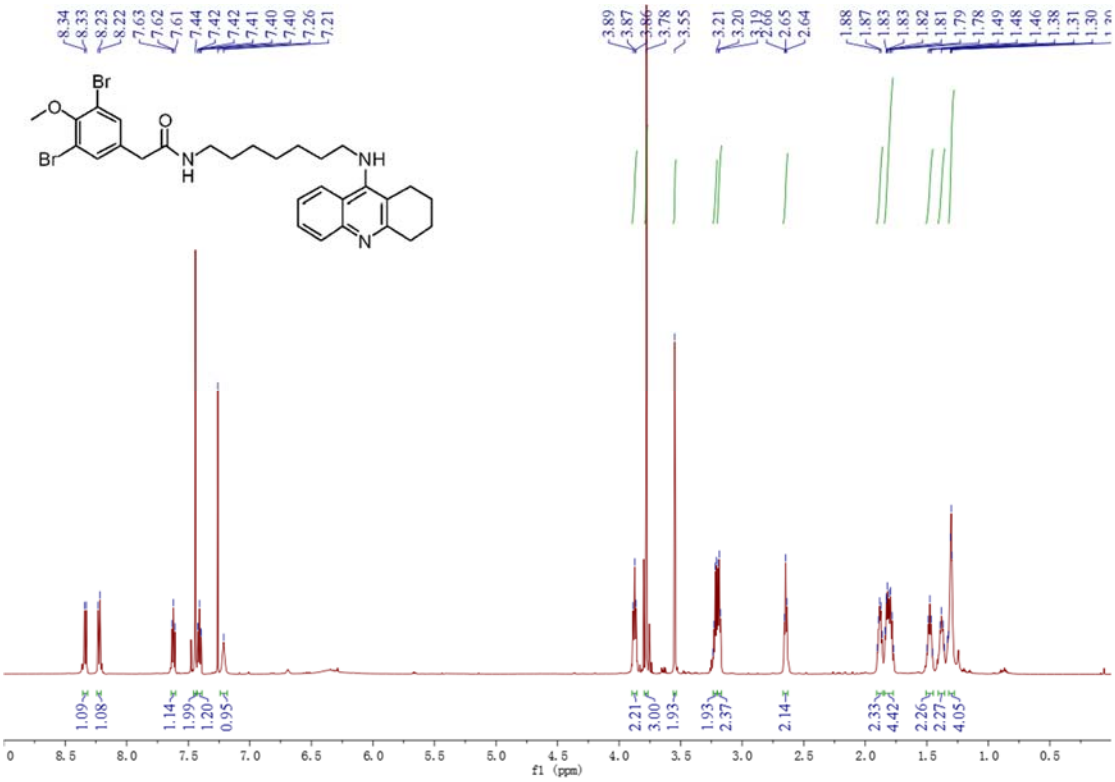

^{13}C NMR spectrum of **5f**

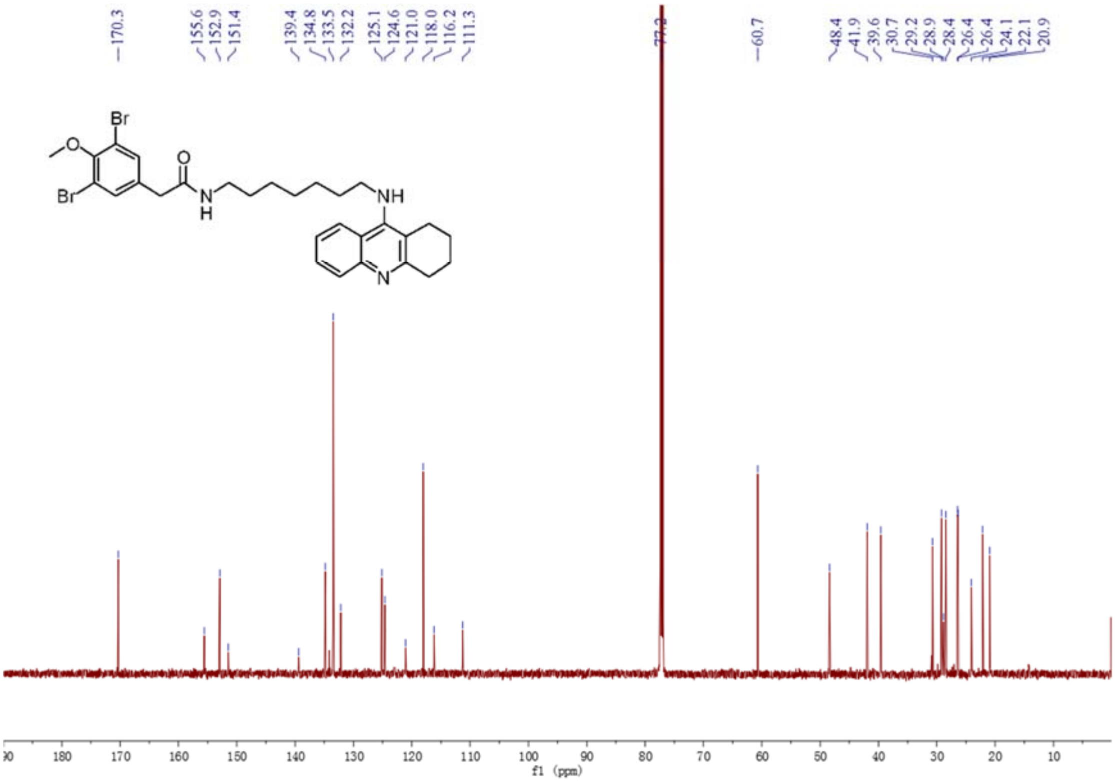

HR-MS (ESI) spectrum of **5f**

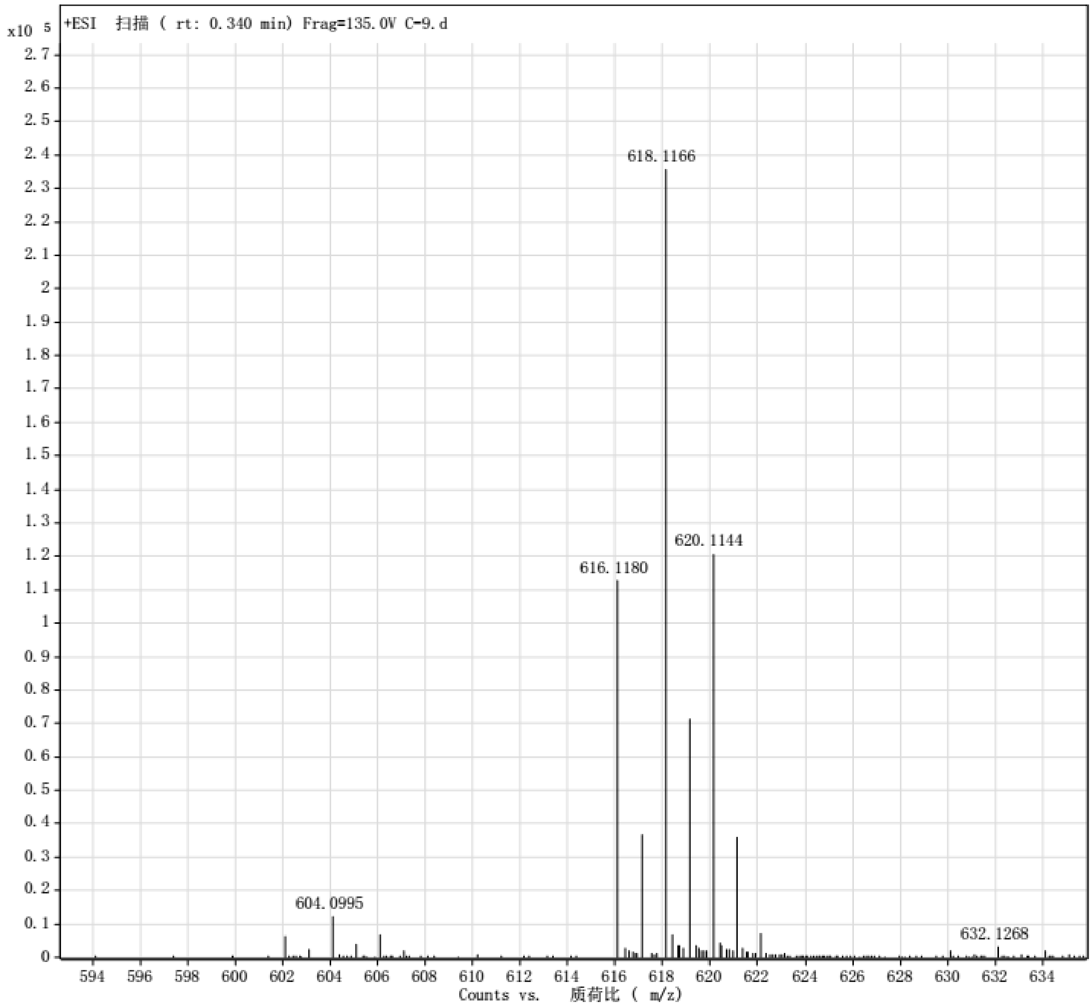

^{1}H NMR spectrum of **5g**

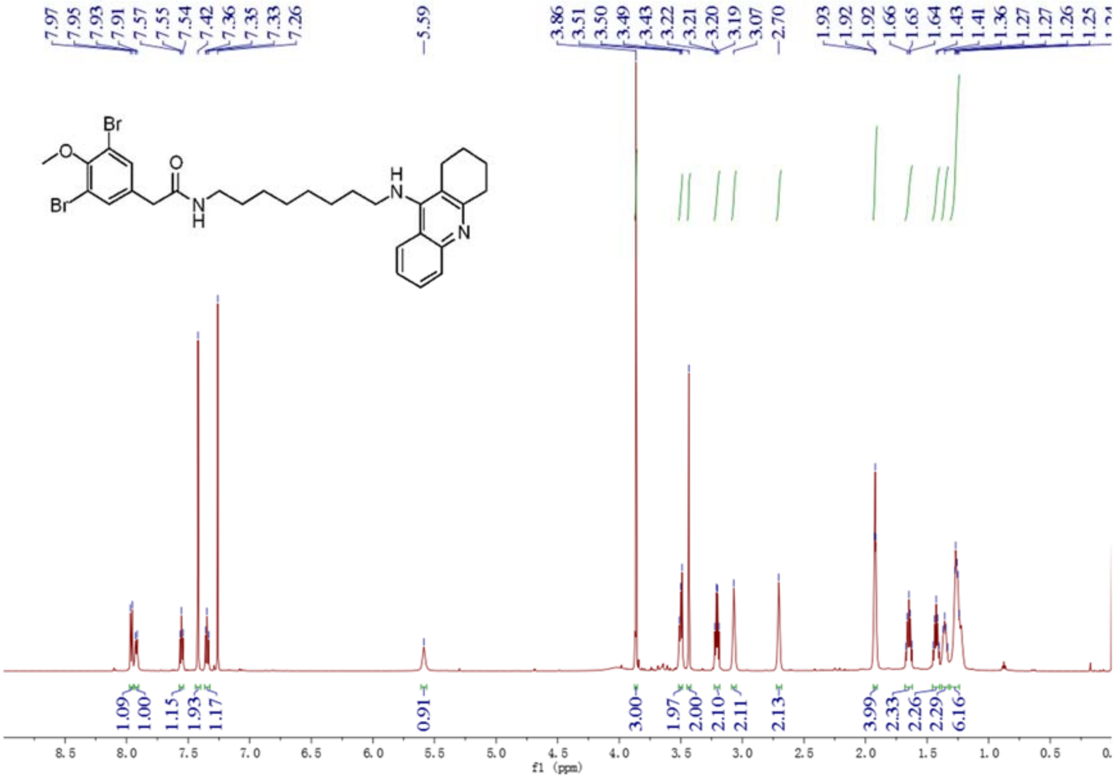

^{13}C NMR spectrum of **5g**

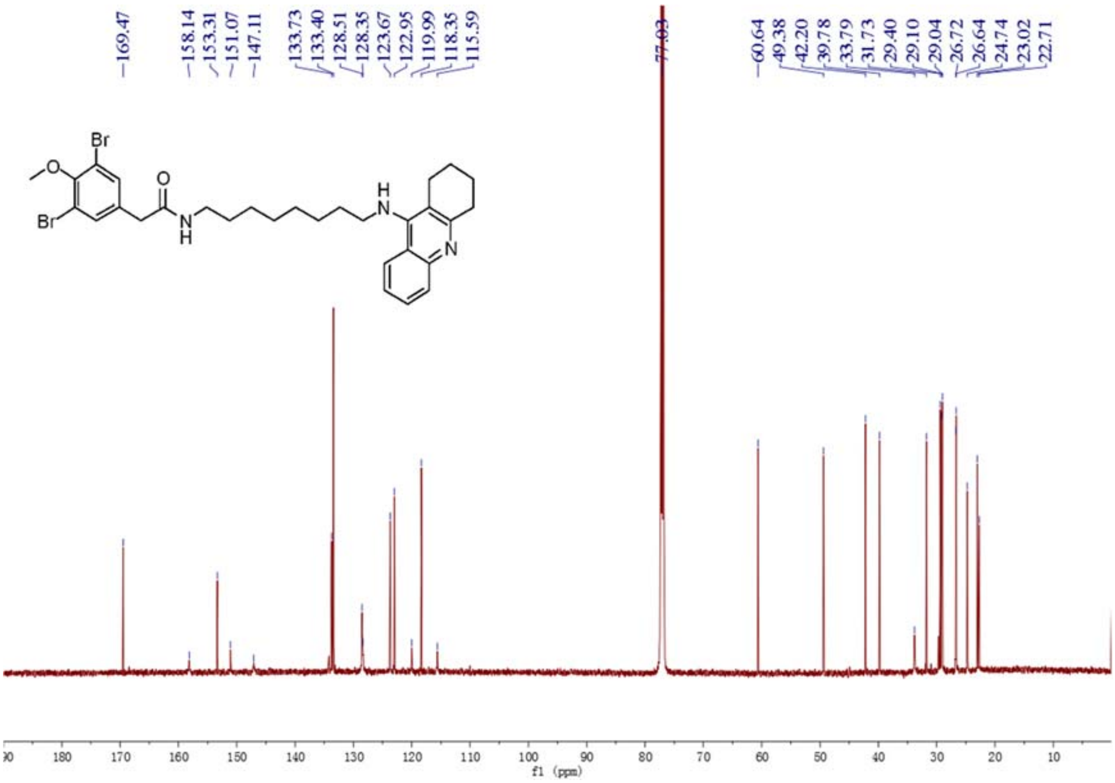

HR-MS (ESI) spectrum of **5g**

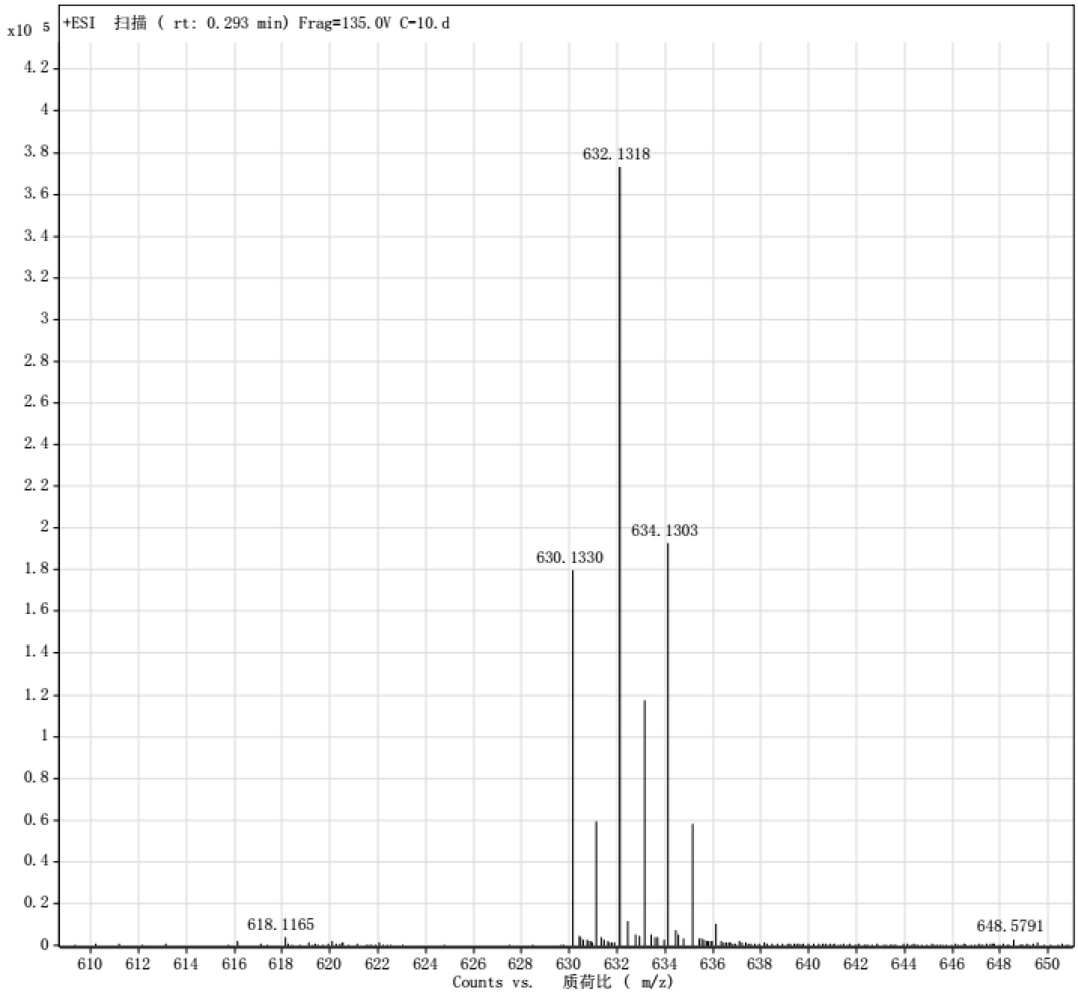

^{1}H NMR spectrum of **5h**

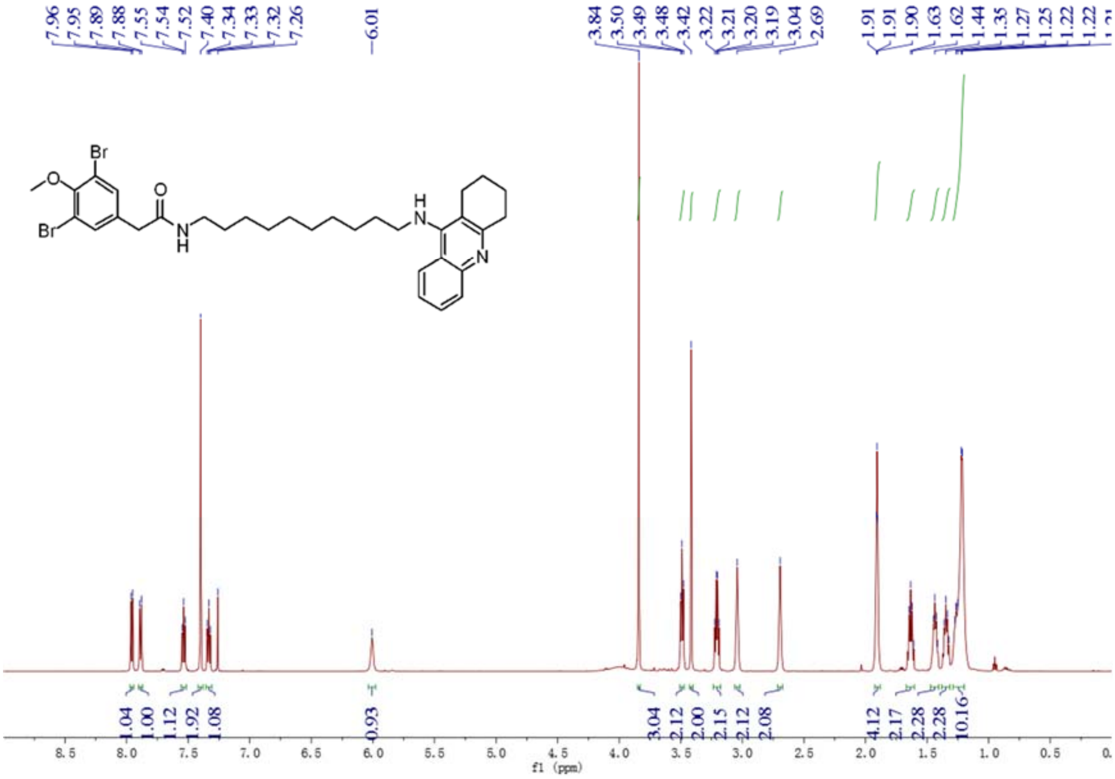

^{13}C NMR spectrum of **5h**

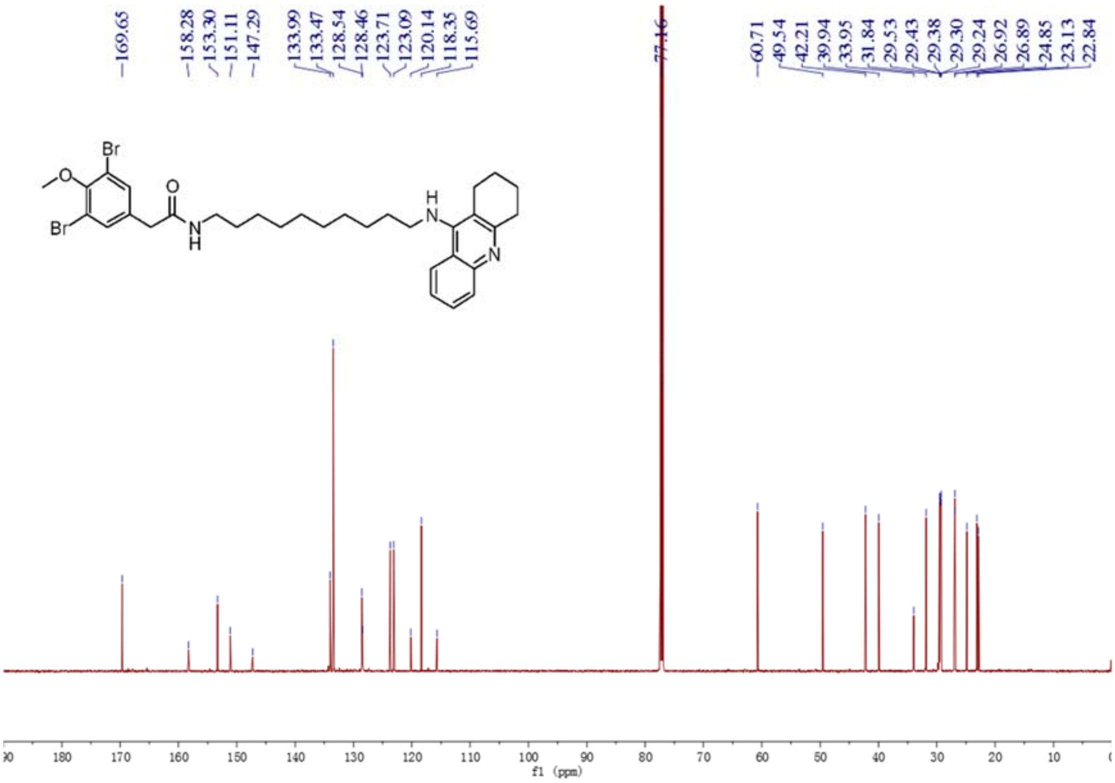

HR-MS (ESI) spectrum of **5h**

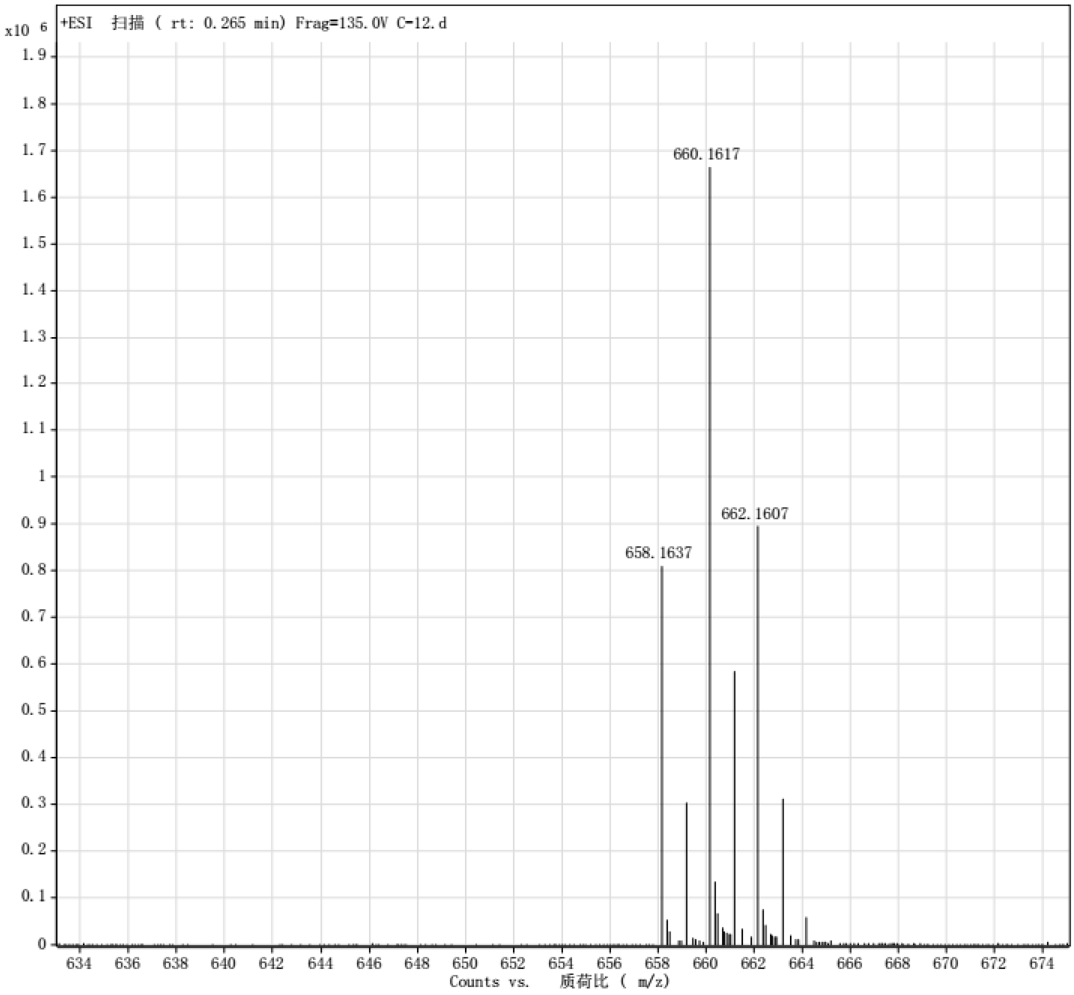

^{1}H NMR spectrum of **12a**

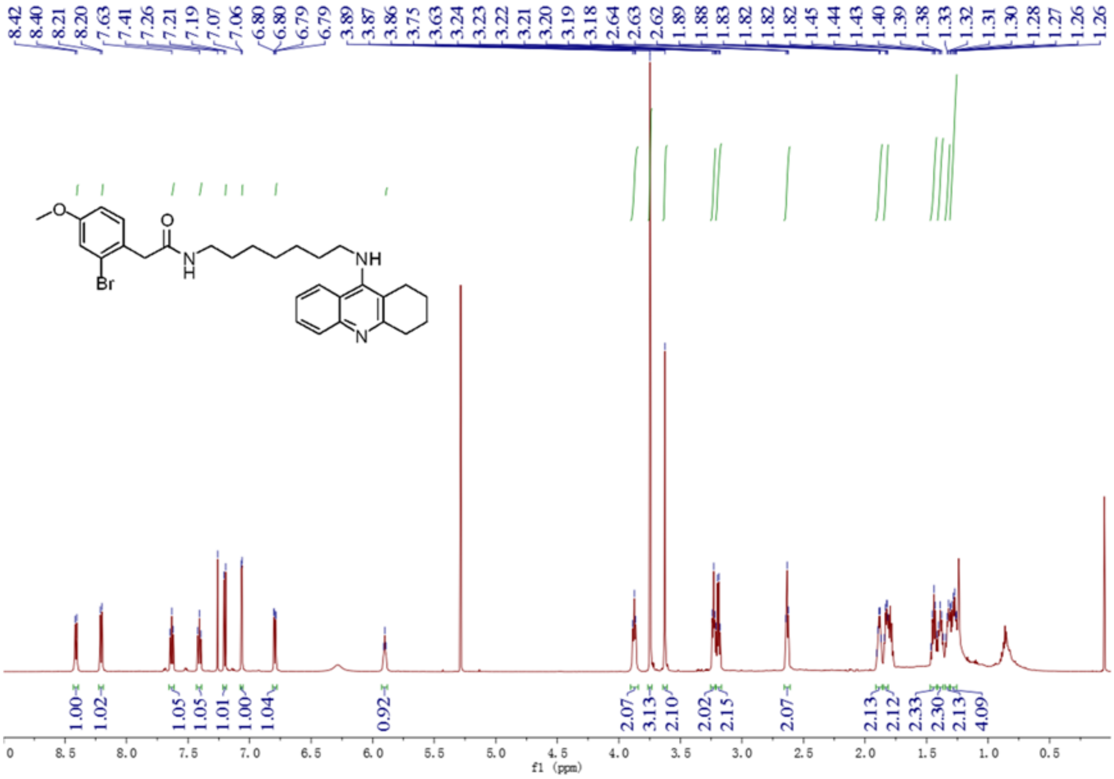

^{13}C NMR spectrum of **12a**

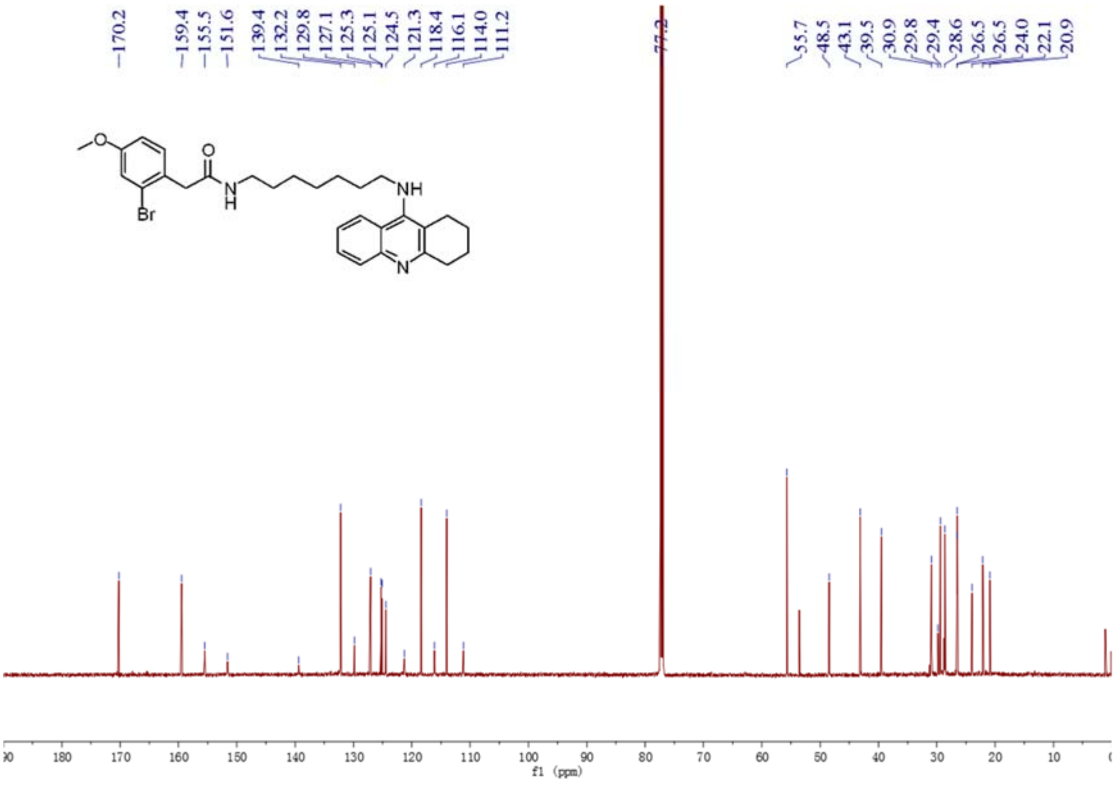

HR-MS (ESI) spectrum of **12a**

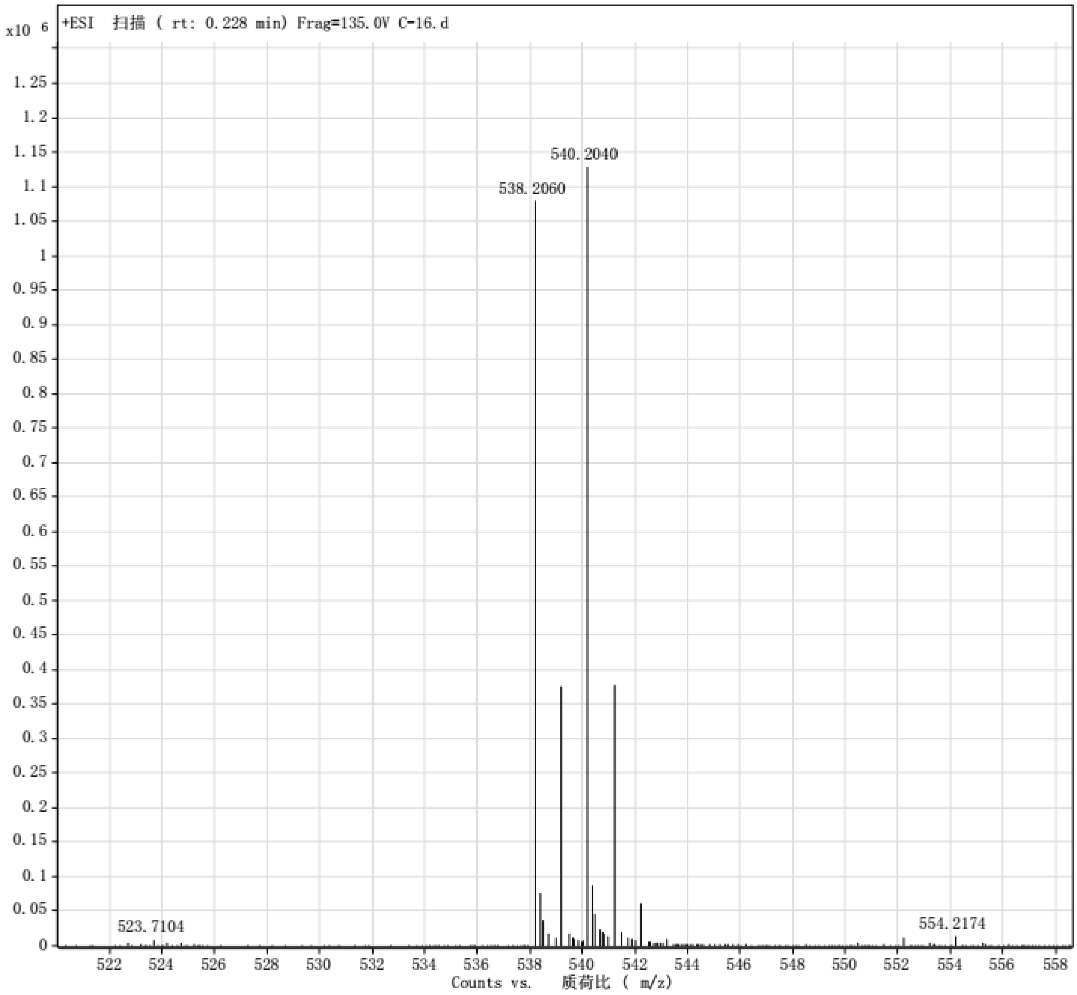

^{1}H NMR spectrum of **12b**

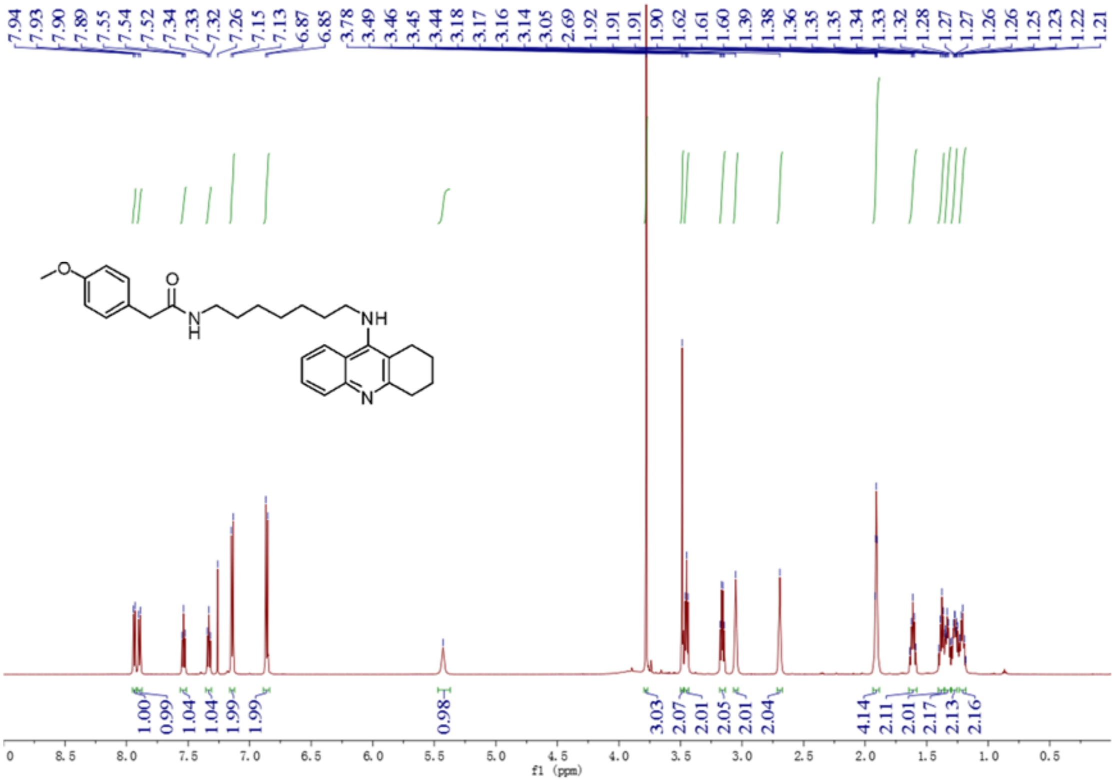

^{13}C NMR spectrum of **12b**

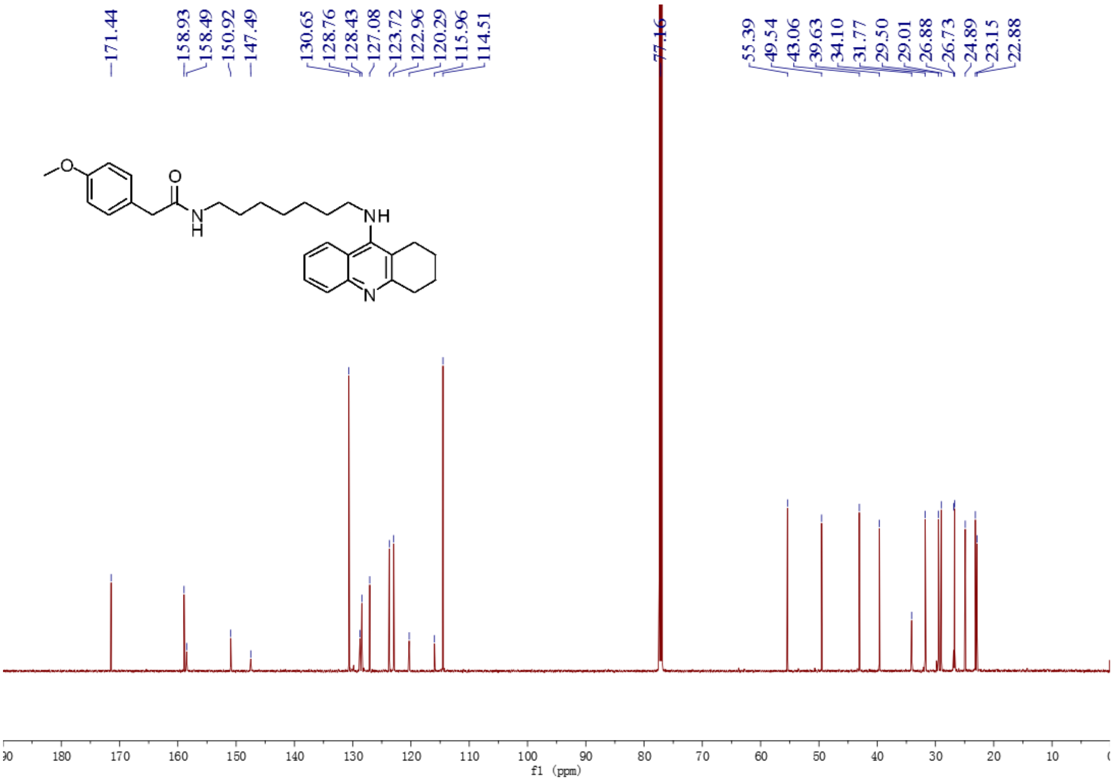

HR-MS (ESI) spectrum of **12b**

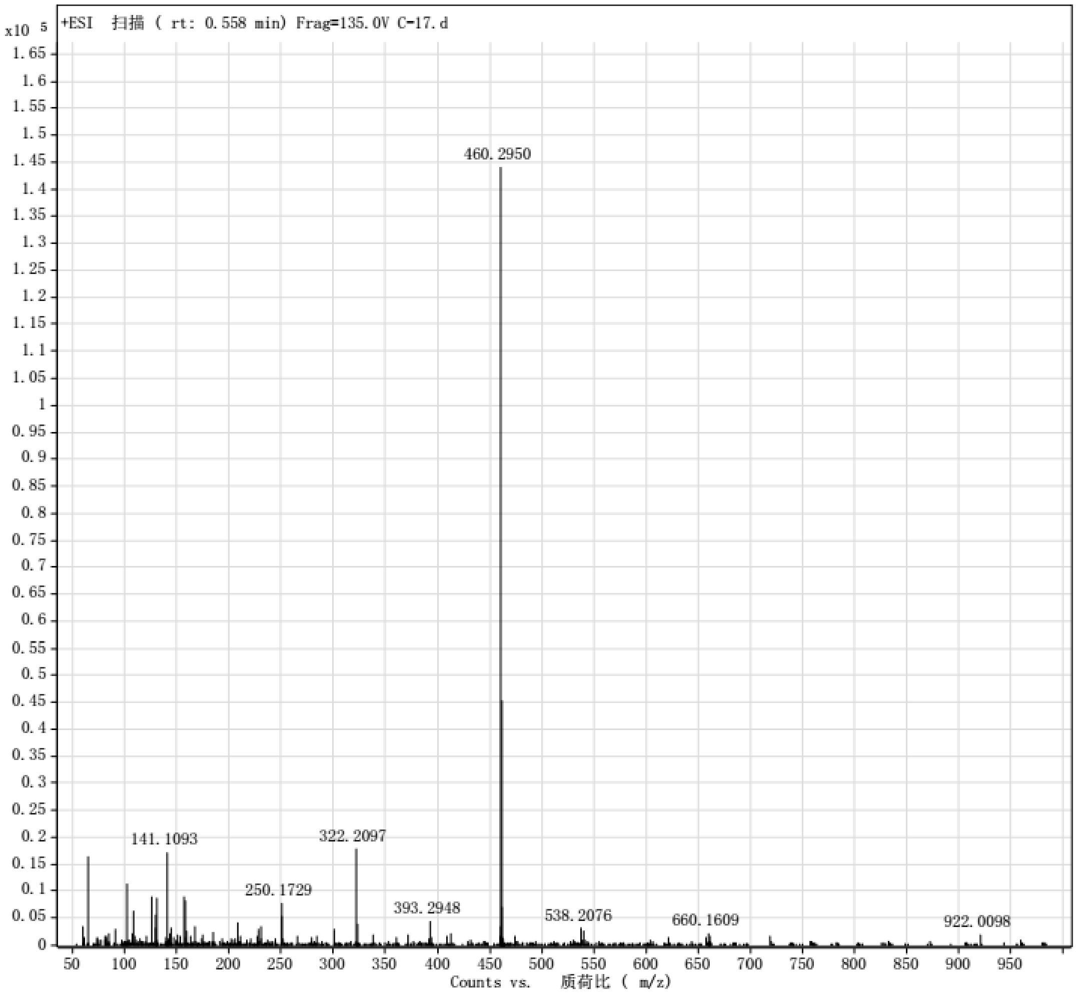

^{1}H NMR spectrum of **12c**

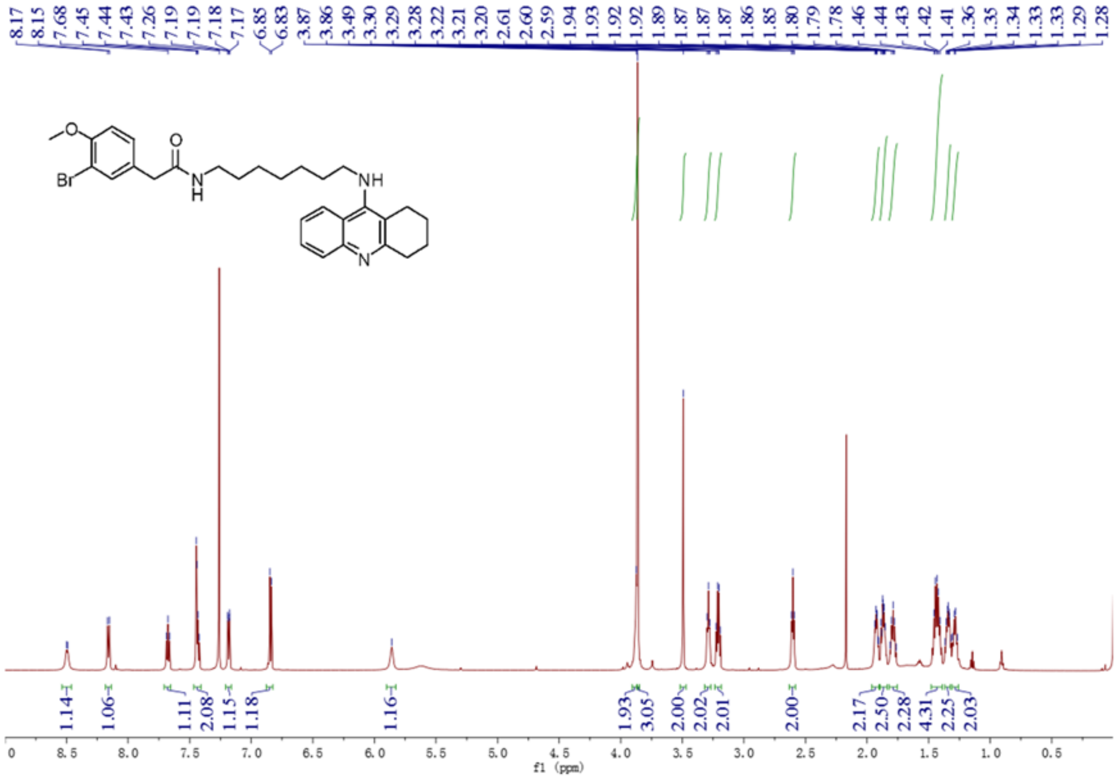

^{13}C NMR spectrum of **12c**

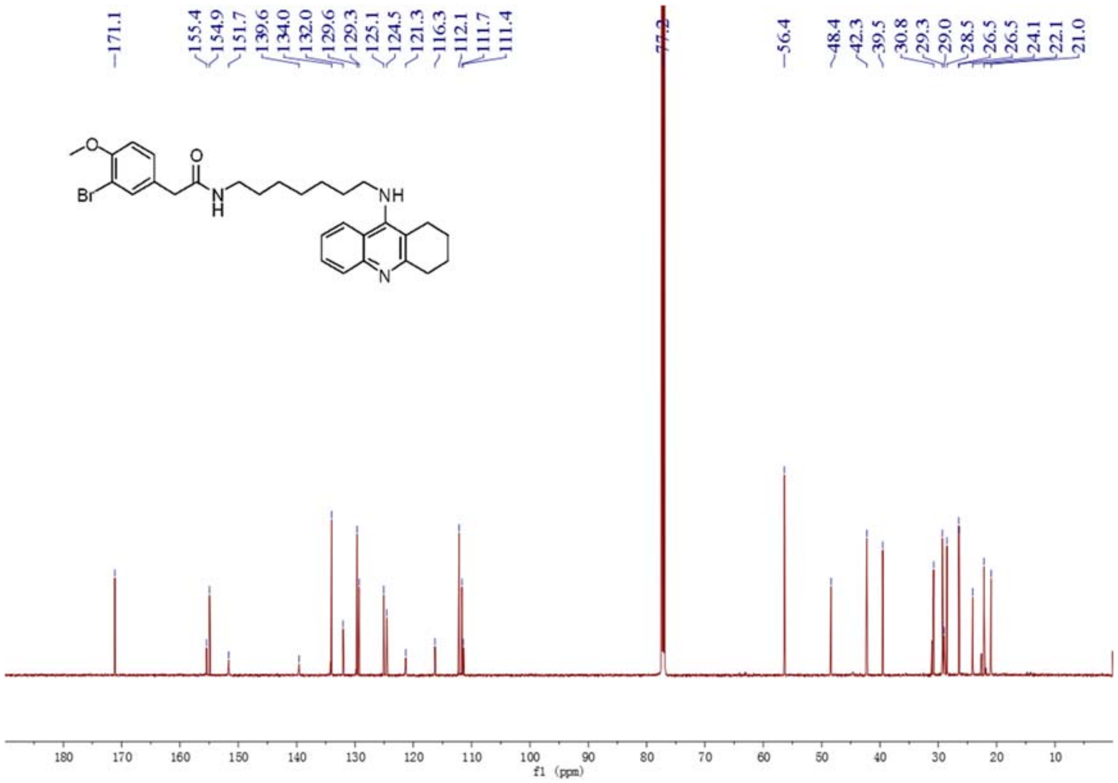

HR-MS (ESI) spectrum of **12c**

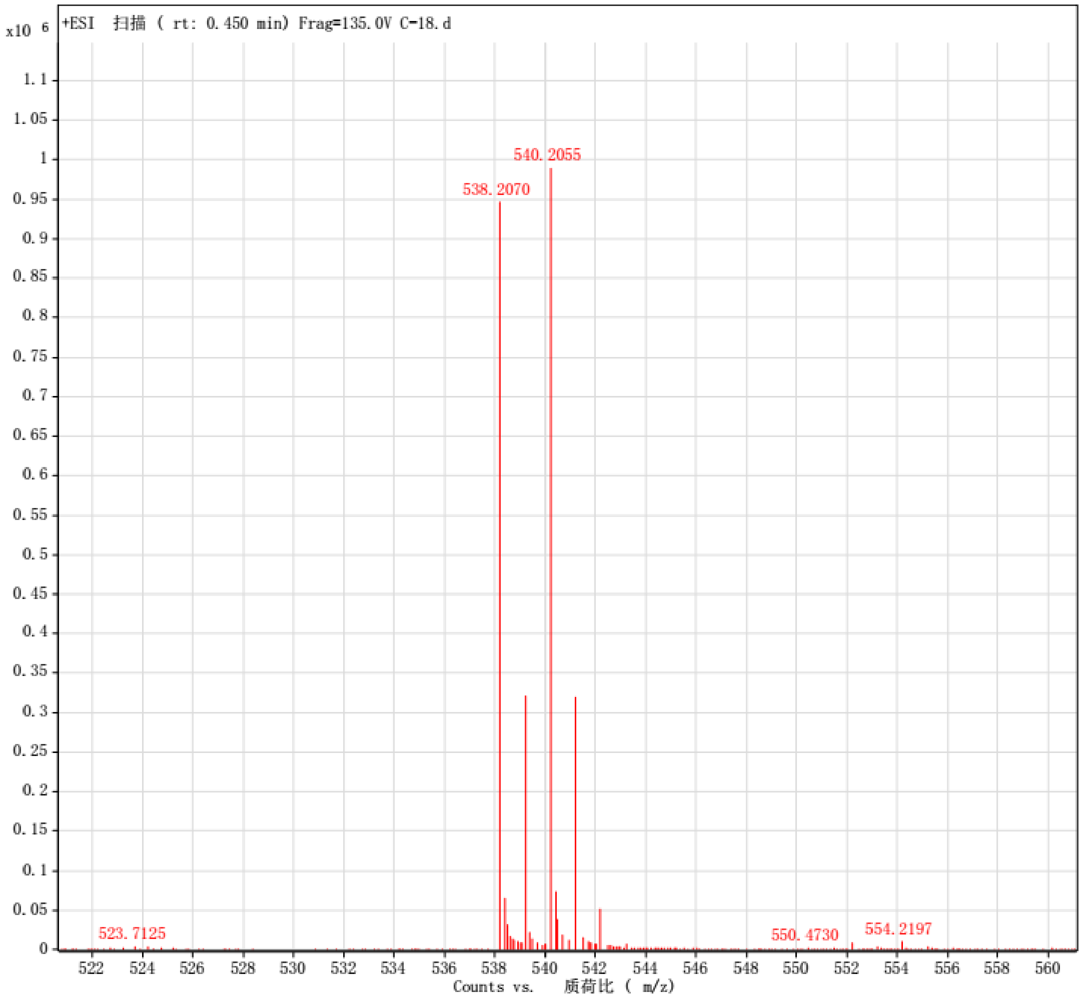

^{1}H NMR spectrum of **12d**

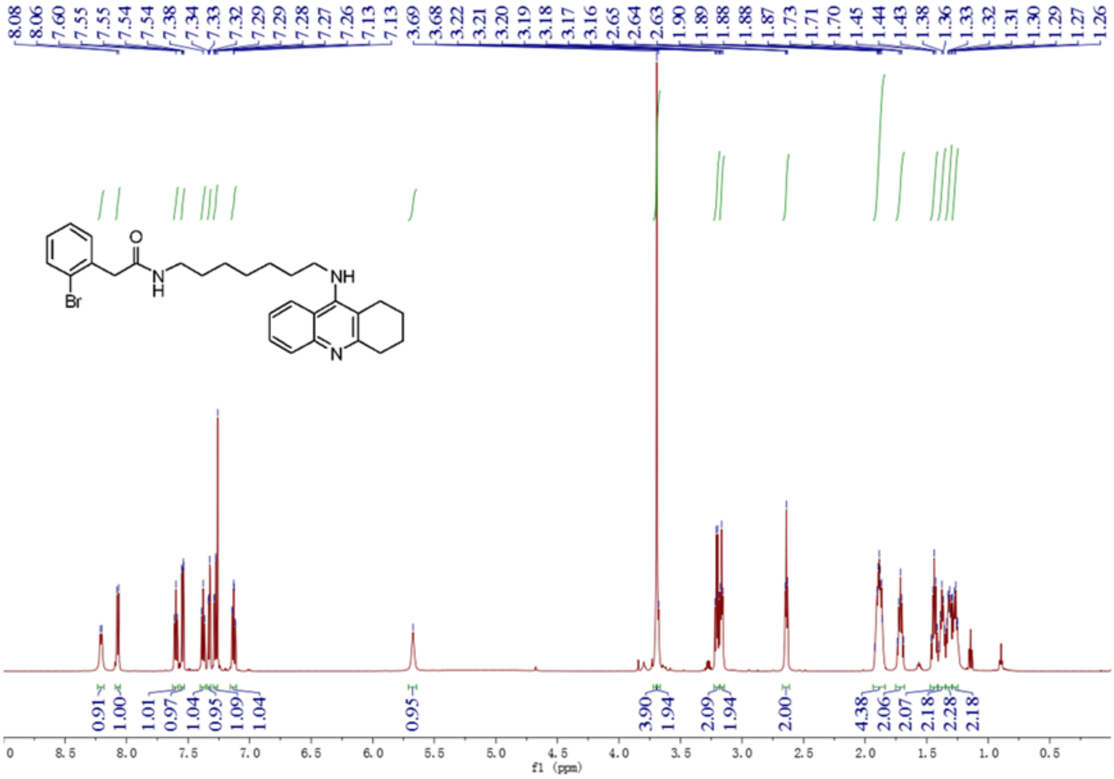

^{13}C NMR spectrum of **12d**

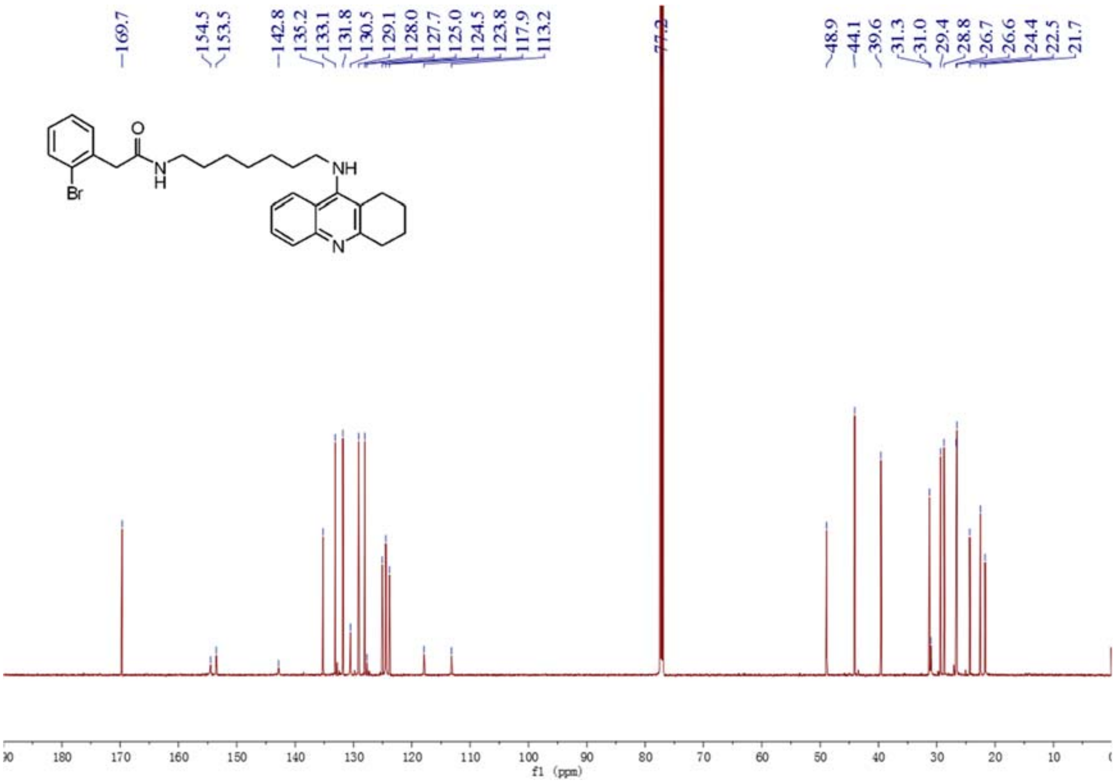

HR-MS (ESI) spectrum of **12d**

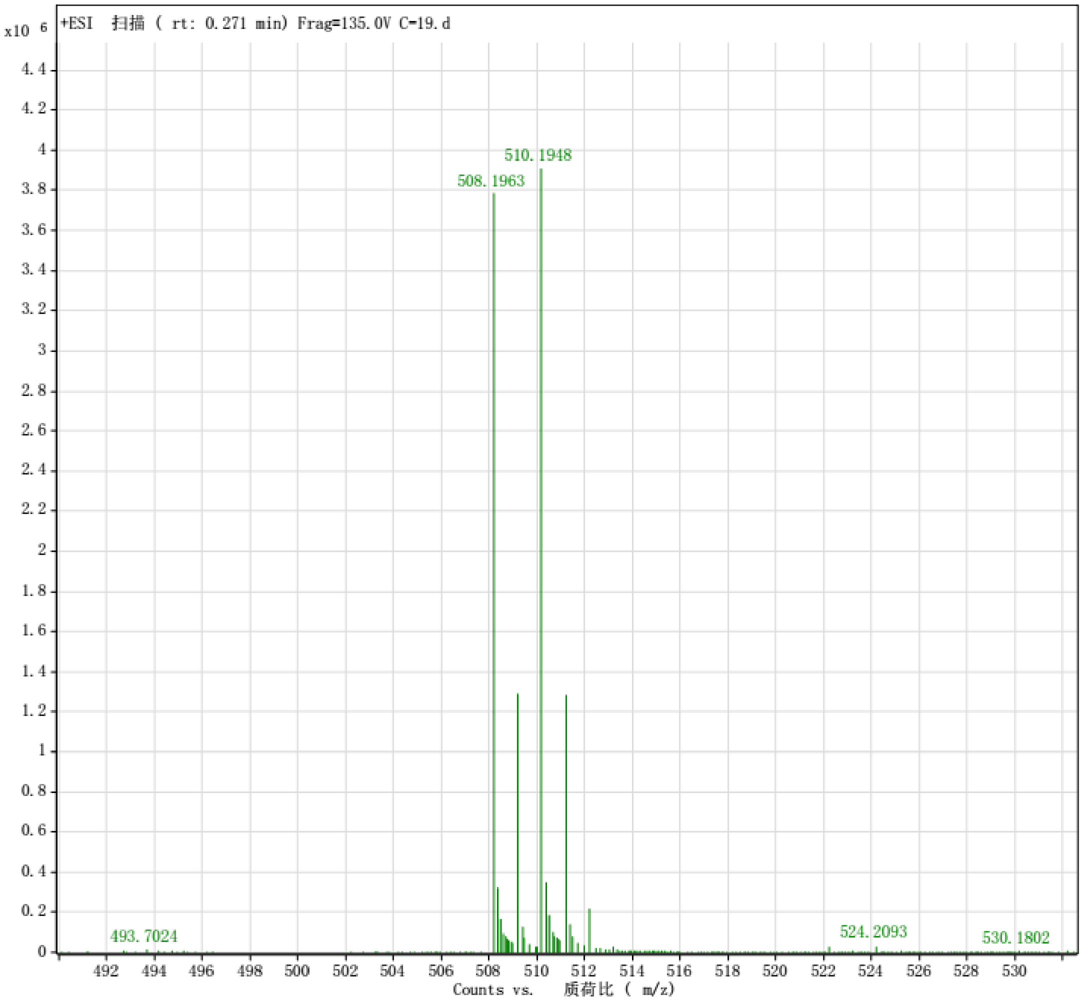

^{1}H NMR spectrum of **12e**

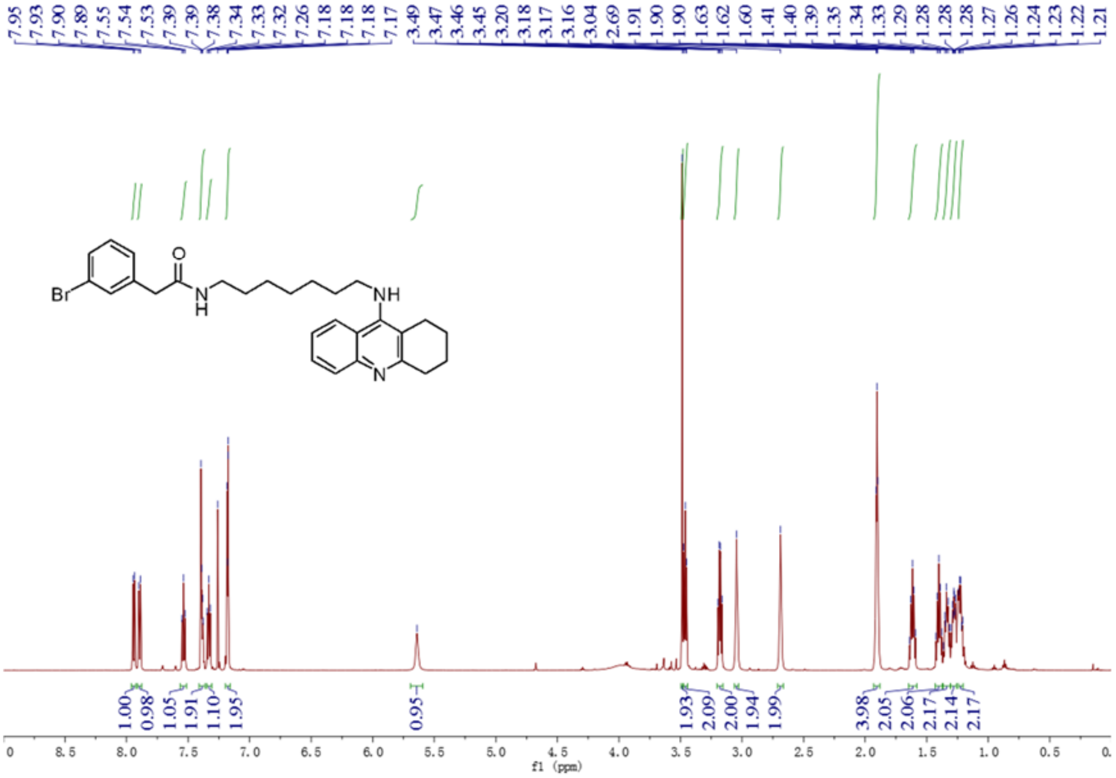

^{13}C NMR spectrum of **12e**

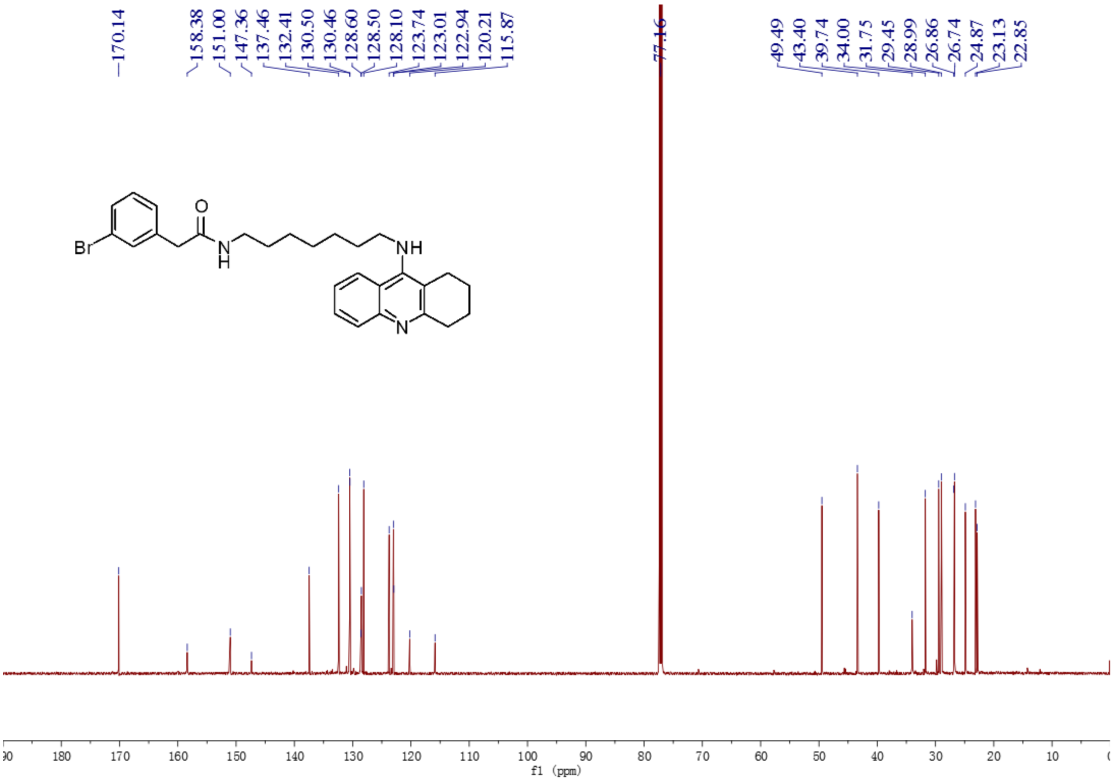

HR-MS (ESI) spectrum of **12e**

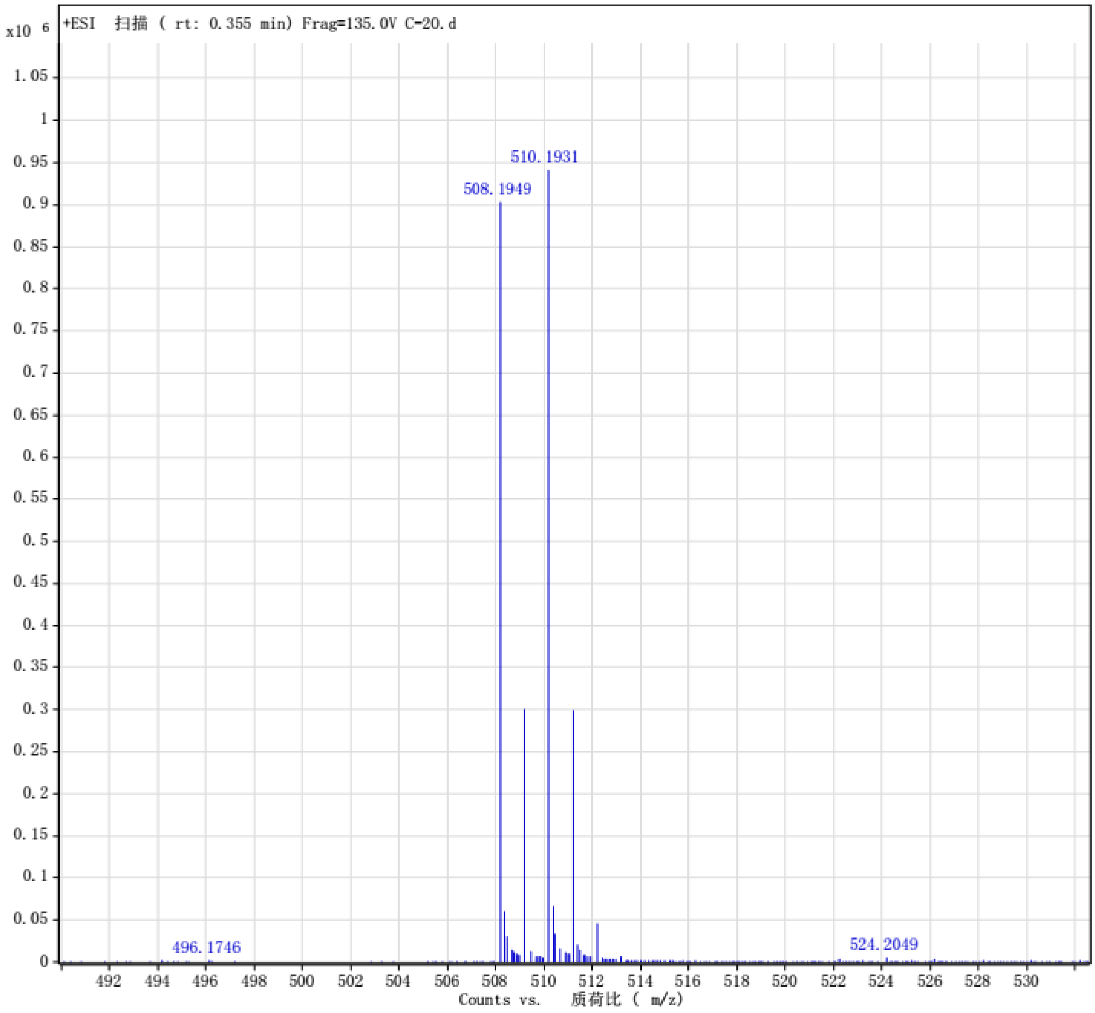

^{1}H NMR spectrum of **12f**

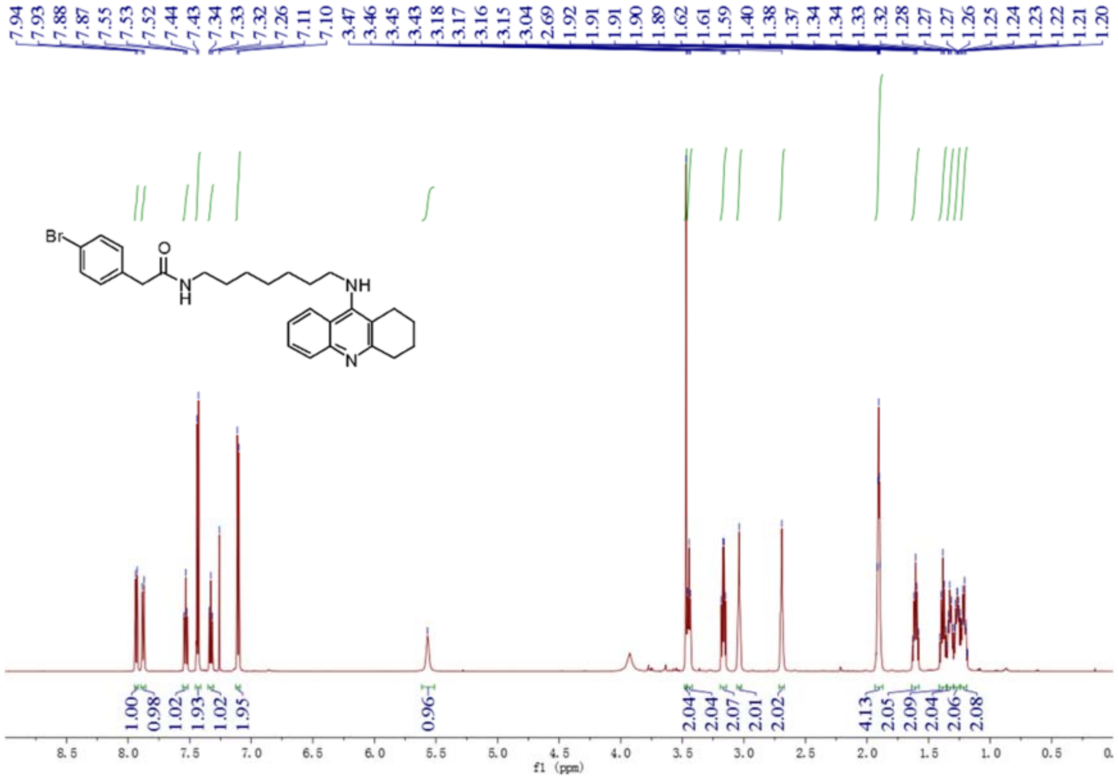

^{13}C NMR spectrum of **12f**

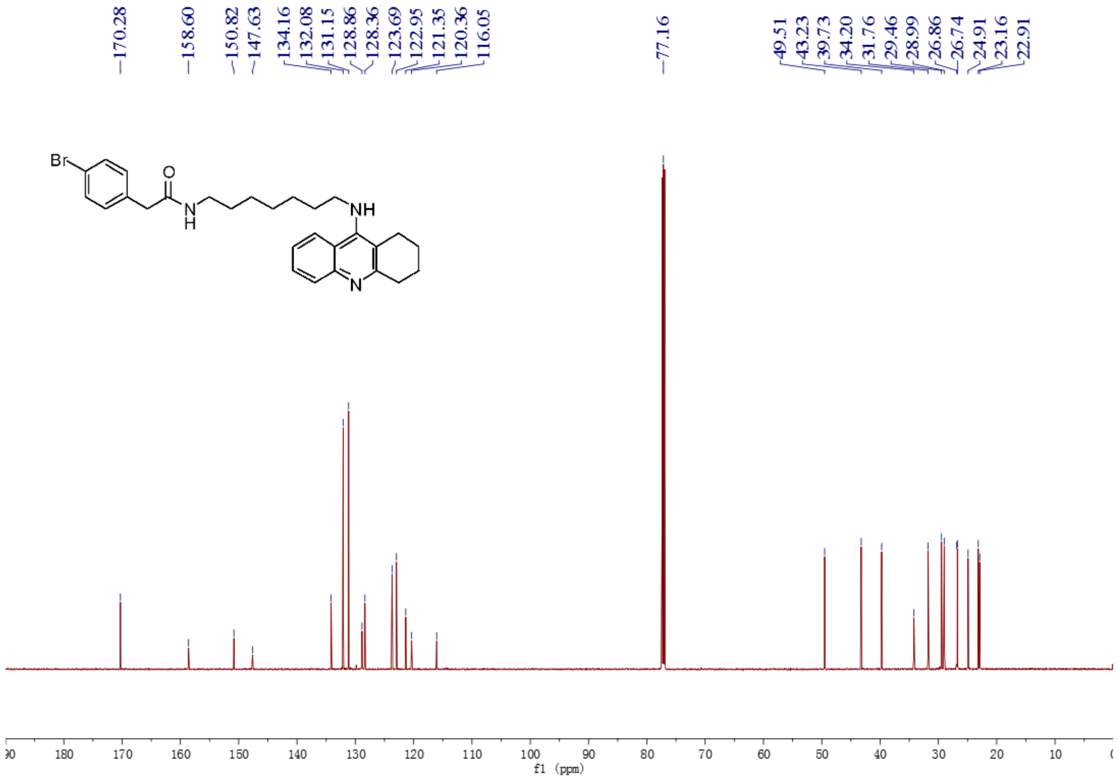

HR-MS (ESI) spectrum of **12f**

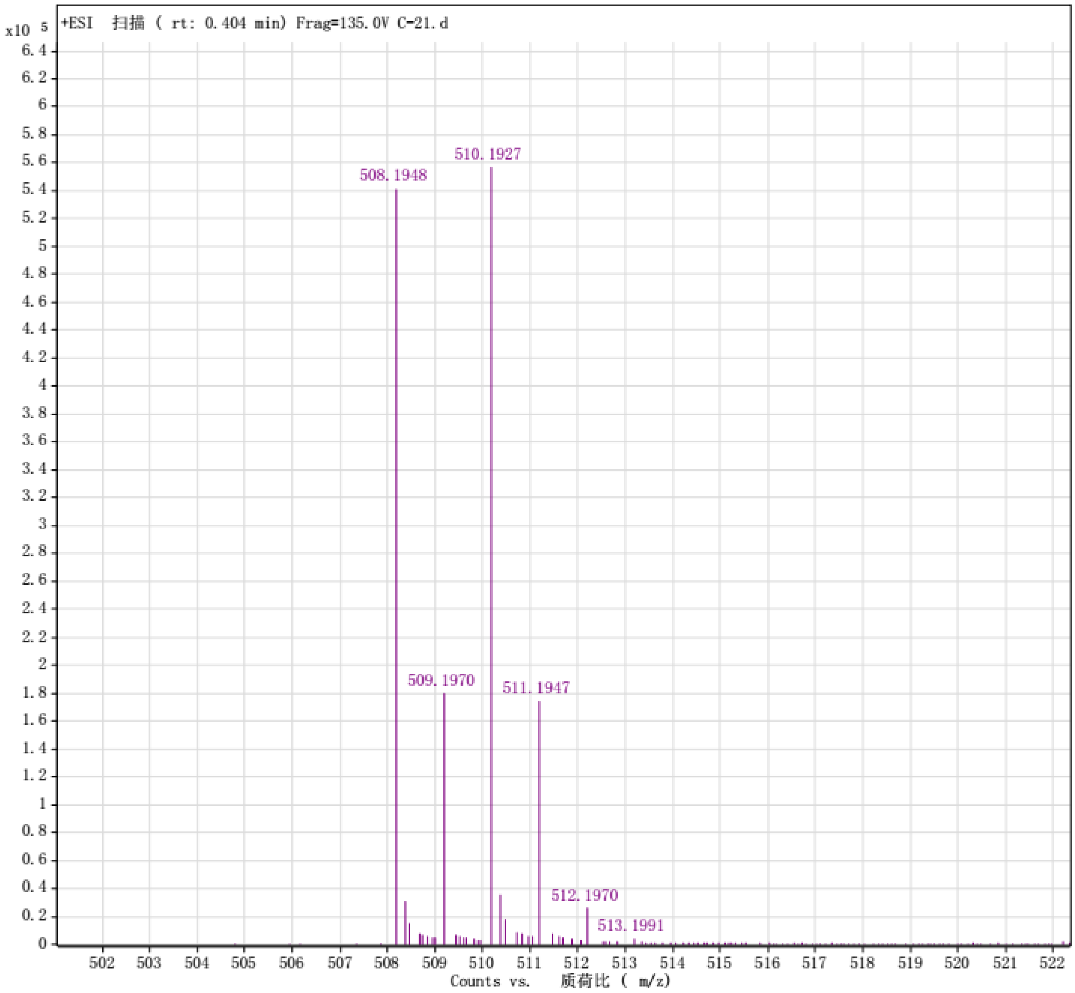

^{1}H NMR spectrum of **12g**

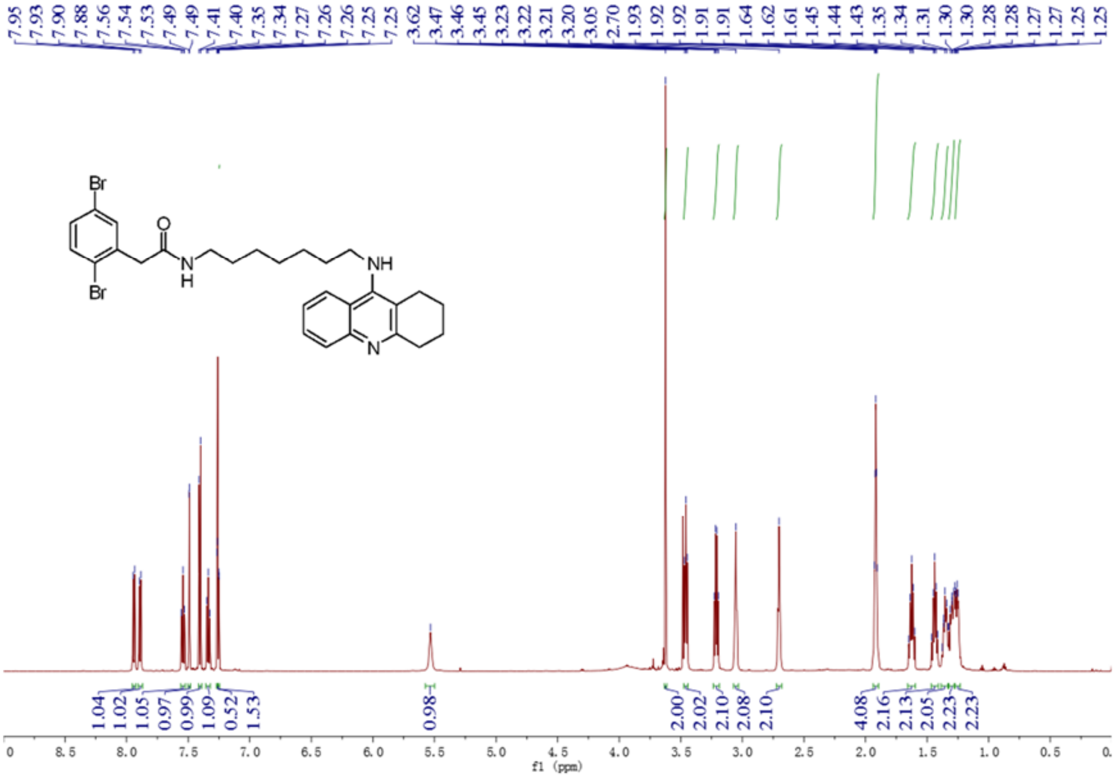

^{13}C NMR spectrum of **12g**

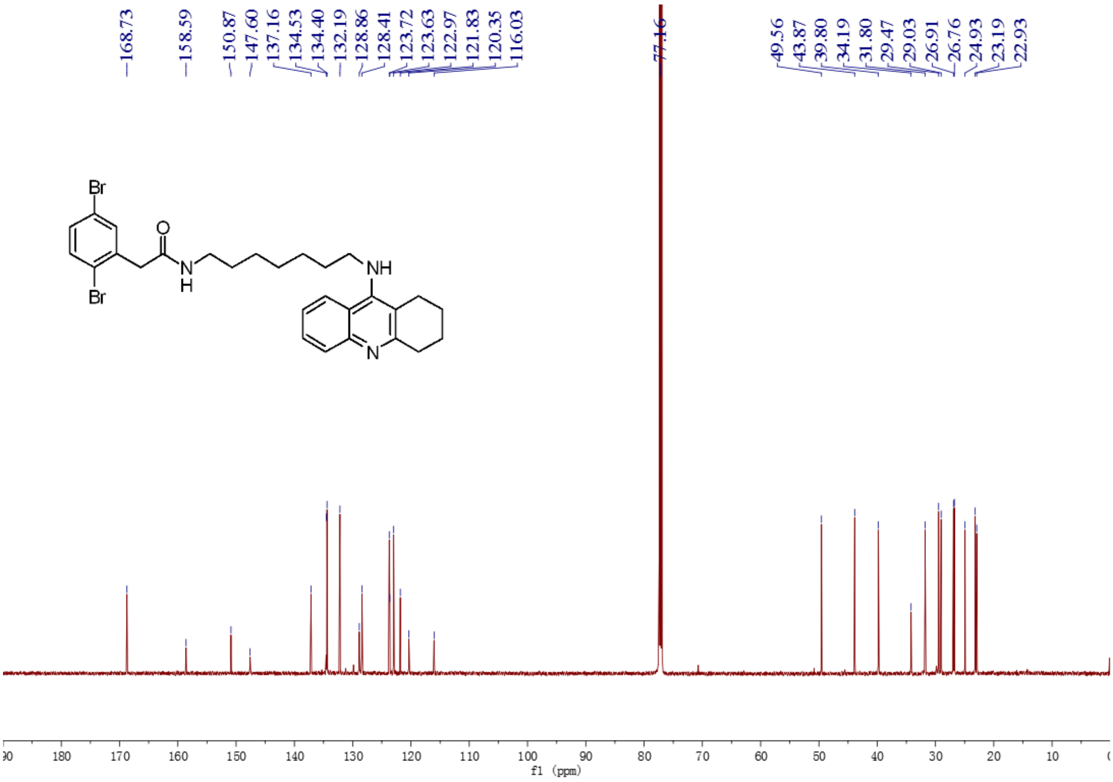

HR-MS (ESI) spectrum of **12g**

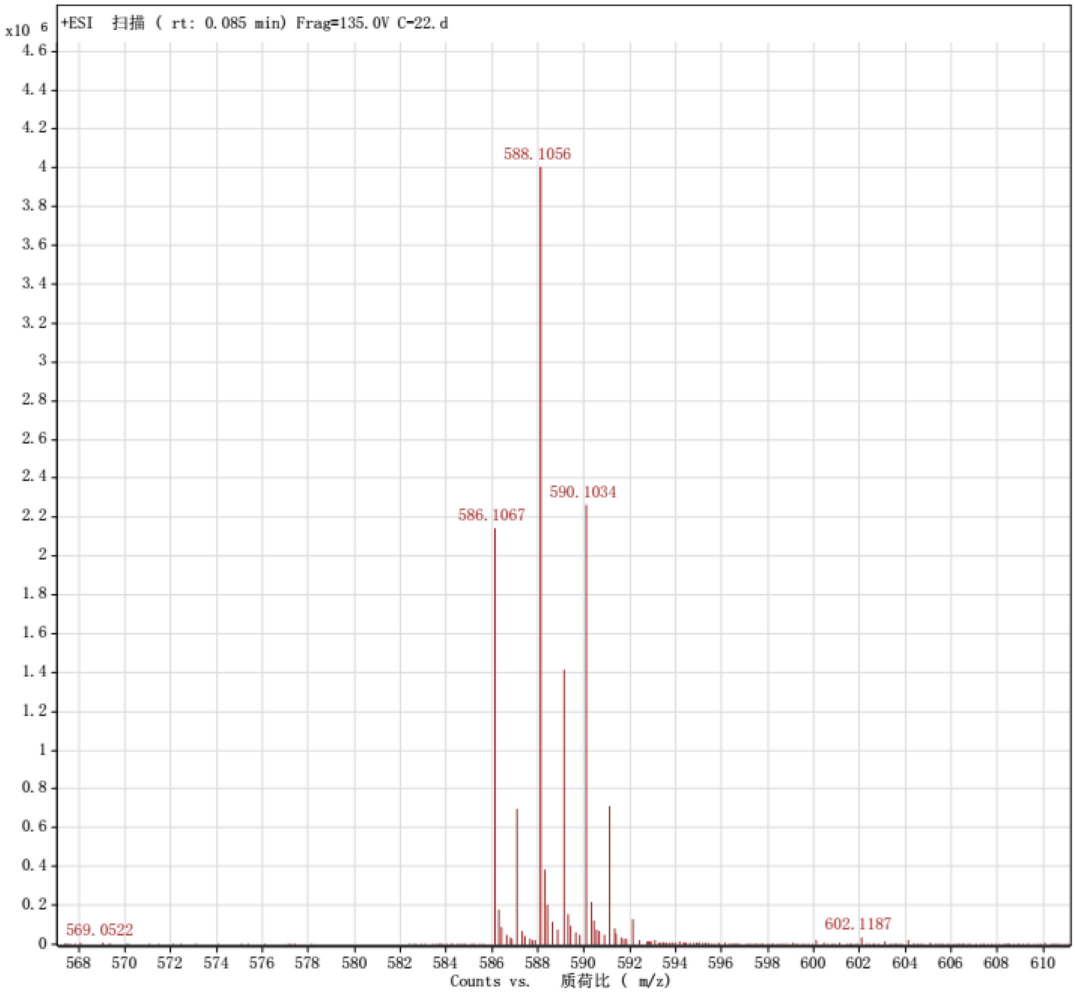

^{1}H NMR spectrum of **12h**

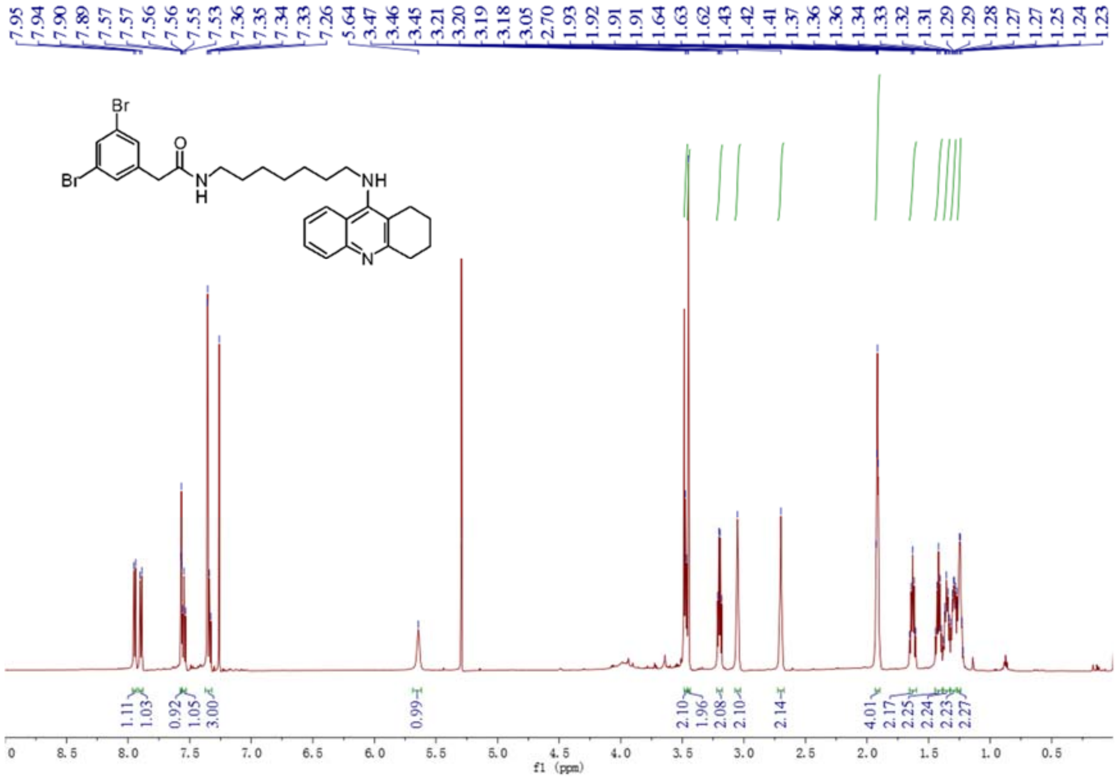

^{13}C NMR spectrum of **12h**

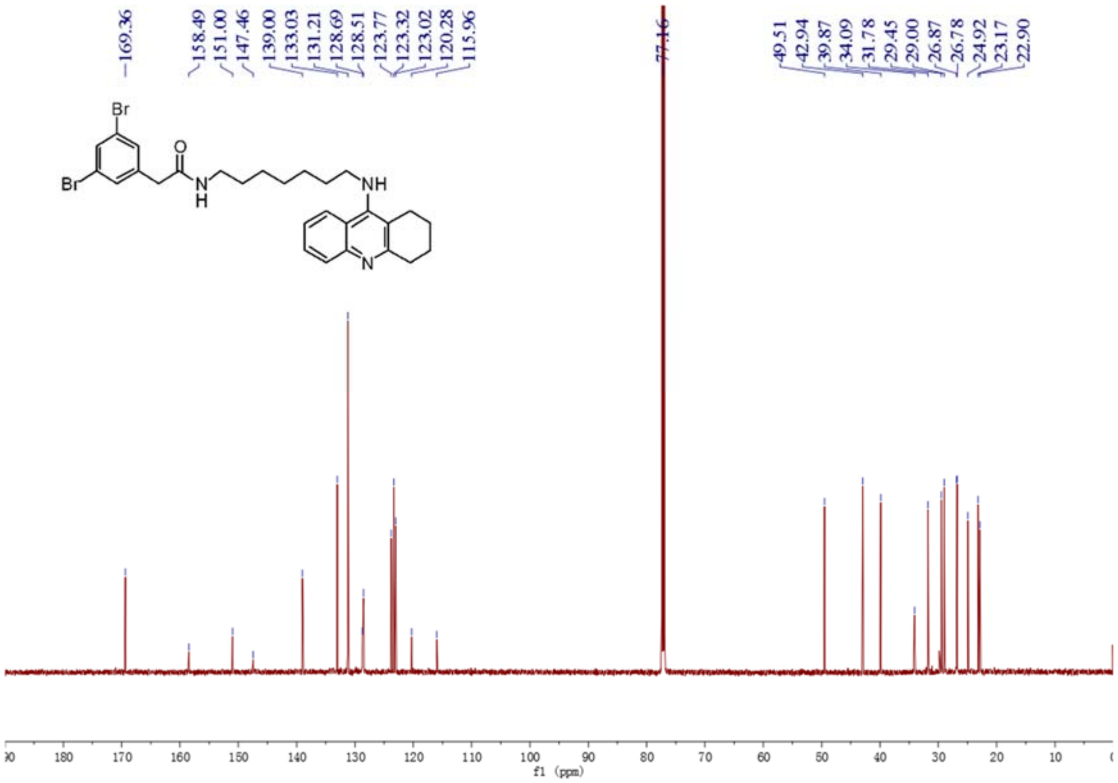

HR-MS (ESI) spectrum of **12h**

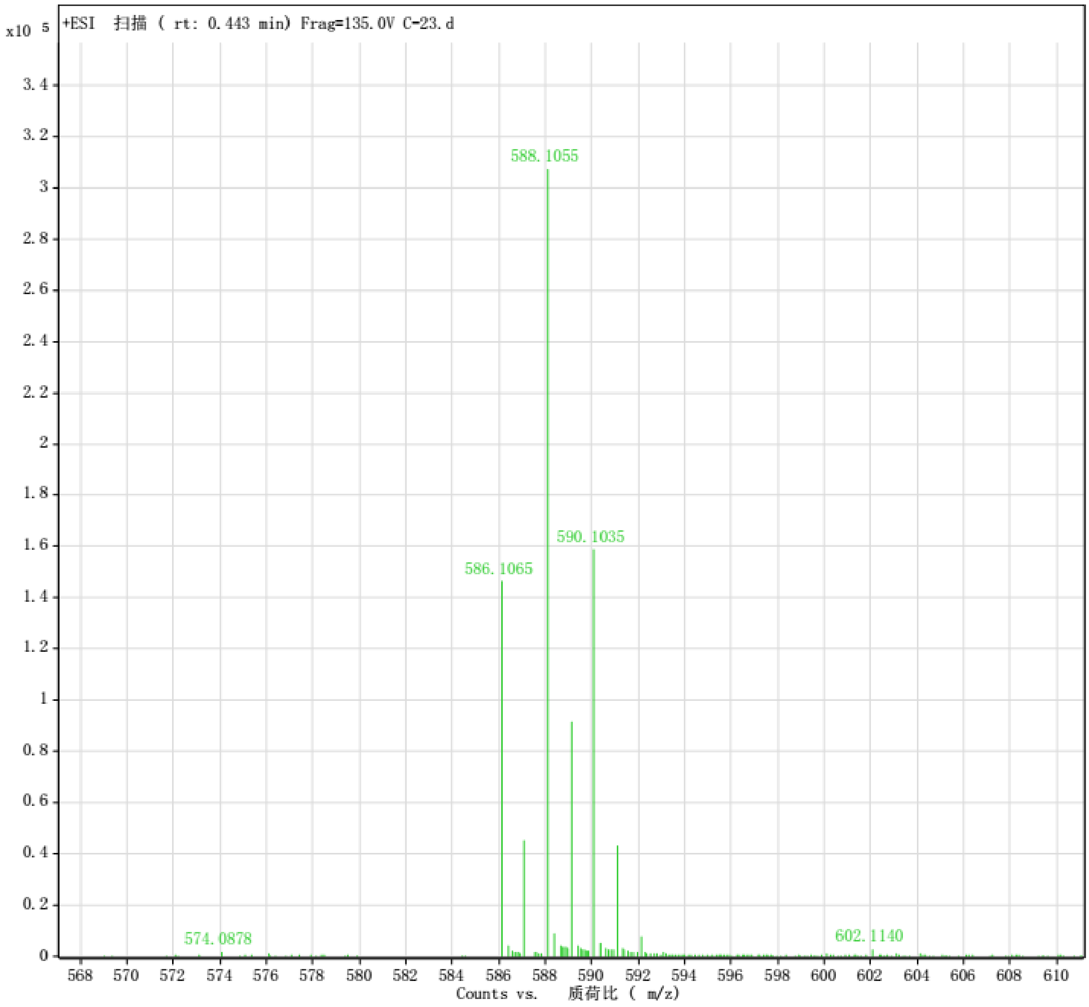

^{1}H NMR spectrum of **12i**

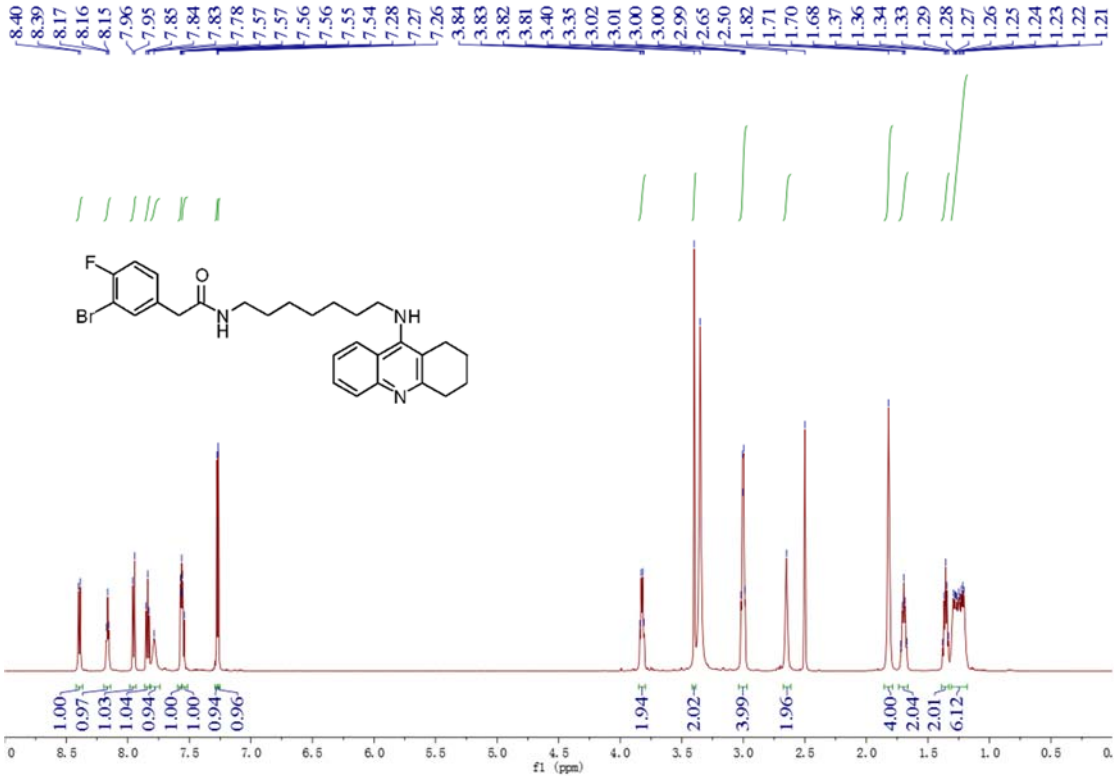

^{13}C NMR spectrum of **12i**

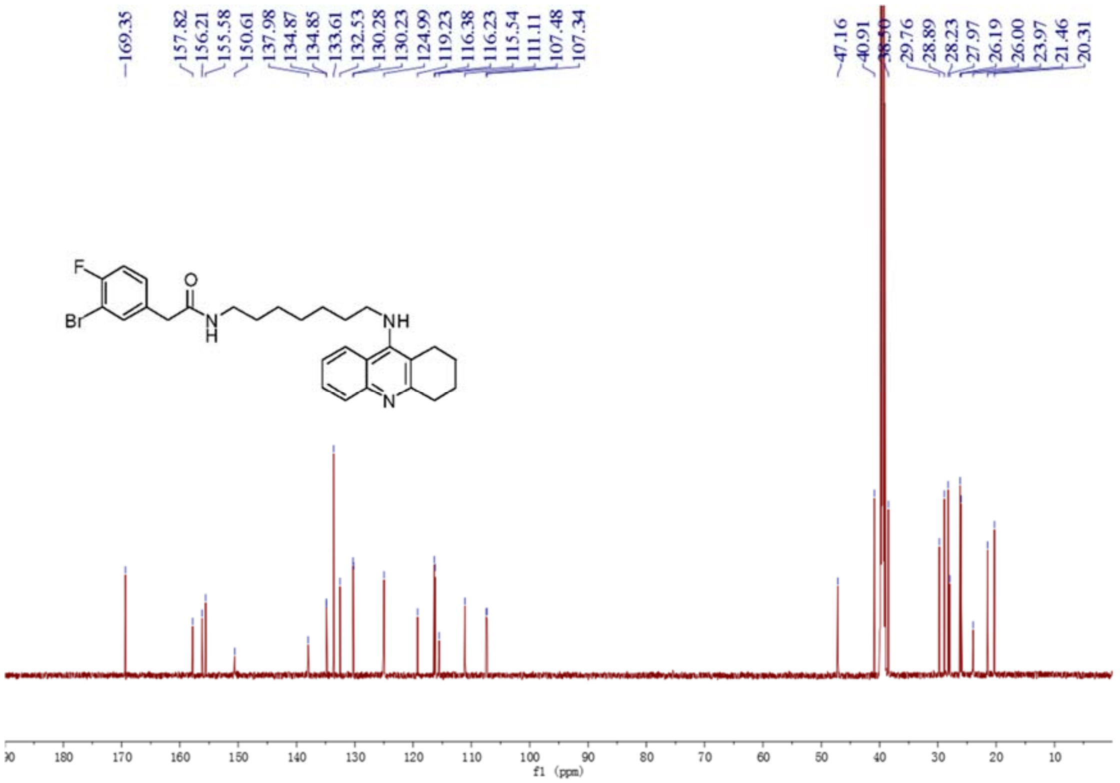

HR-MS (ESI) spectrum of **12i**

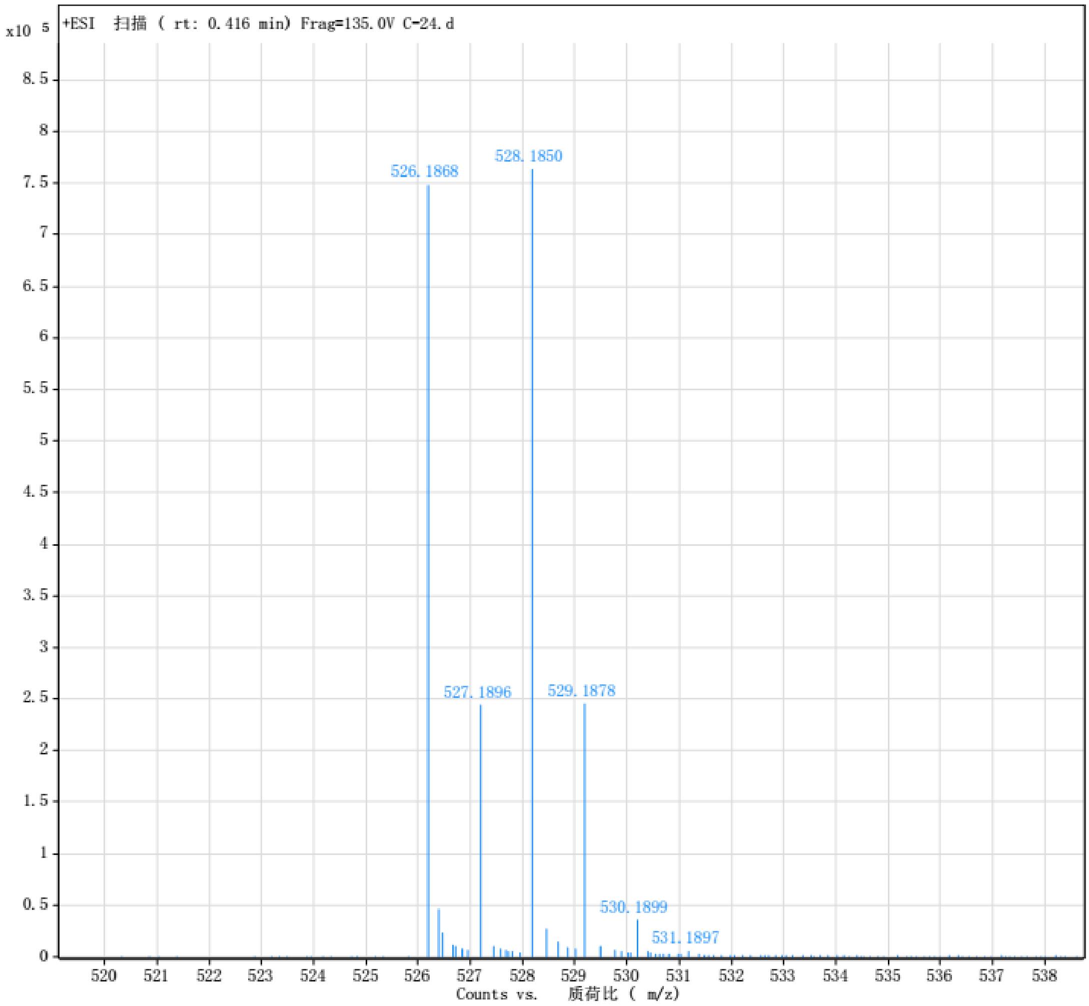

^{1}H NMR spectrum of **12j**

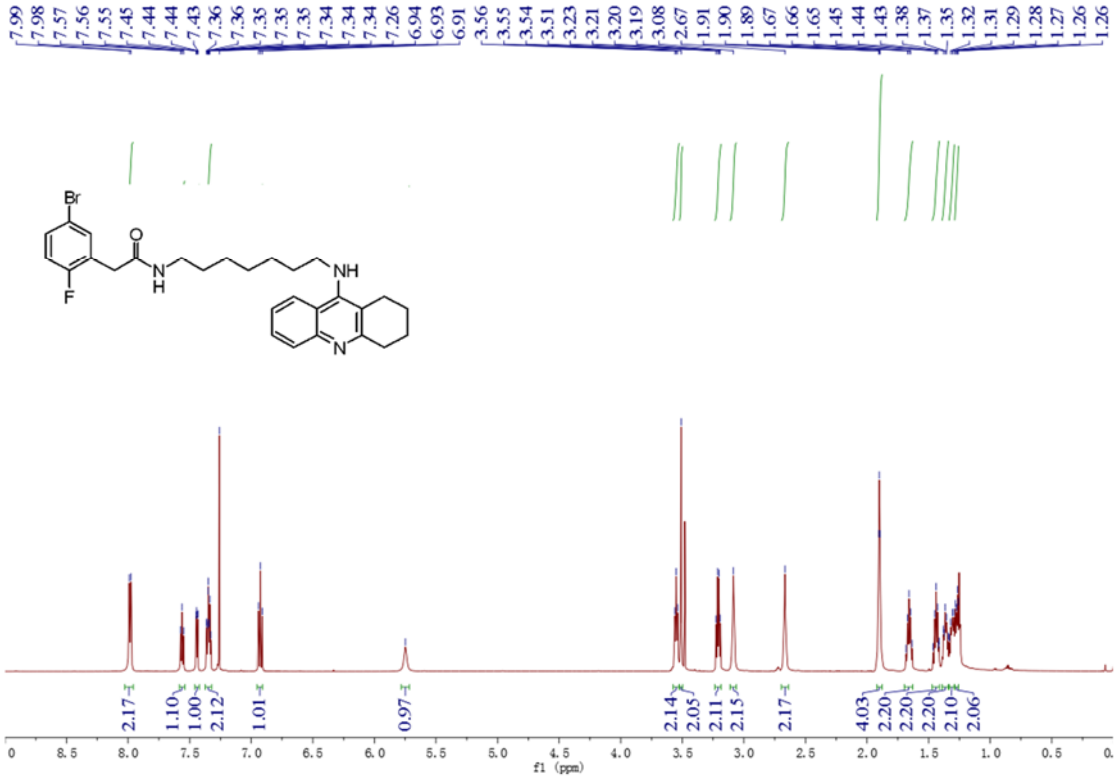

^{13}C NMR spectrum of **12j**

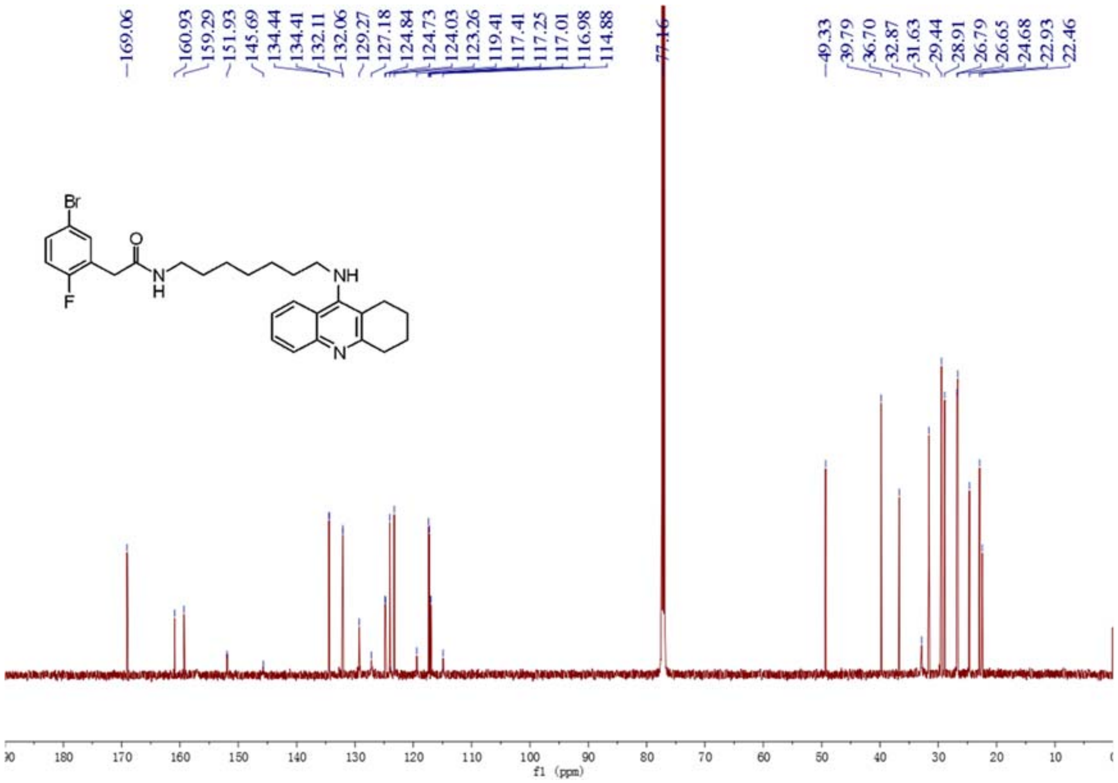

HR-MS (ESI) spectrum of **12j**

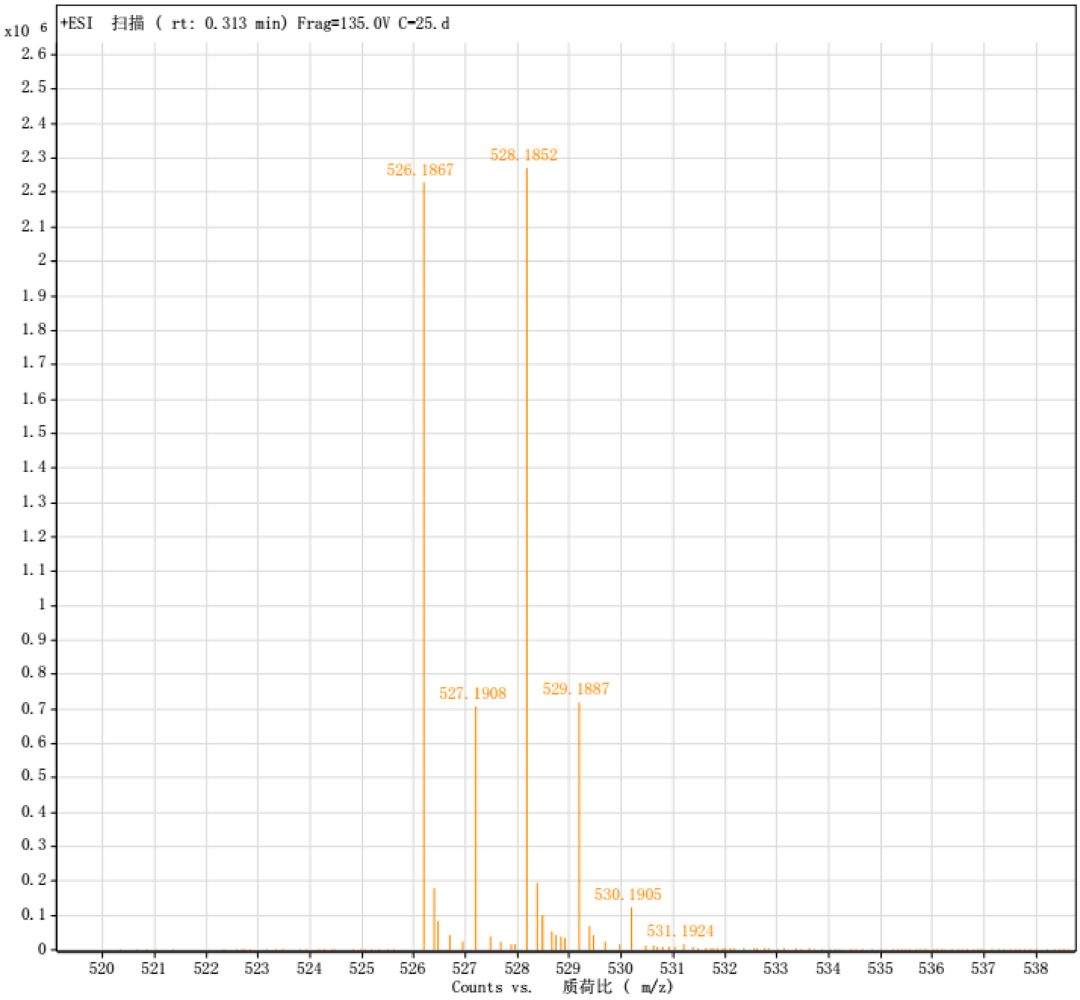

^{1}H NMR spectrum of **12k**

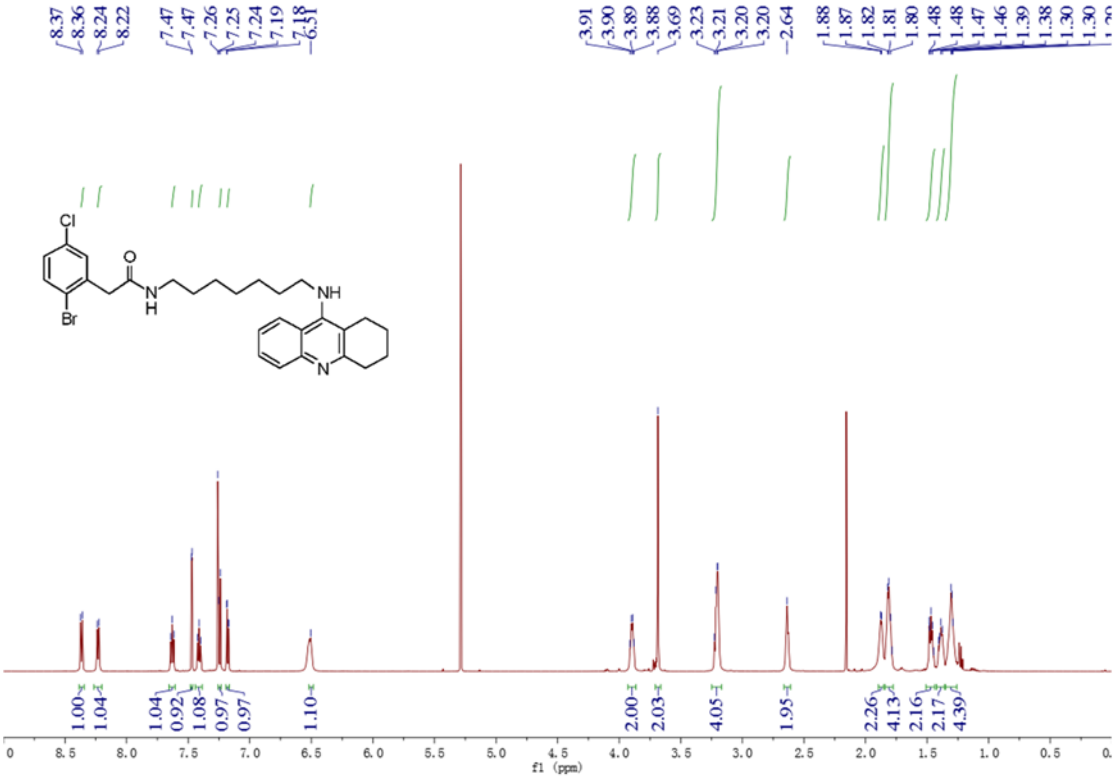

^{13}C NMR spectrum of **12k**

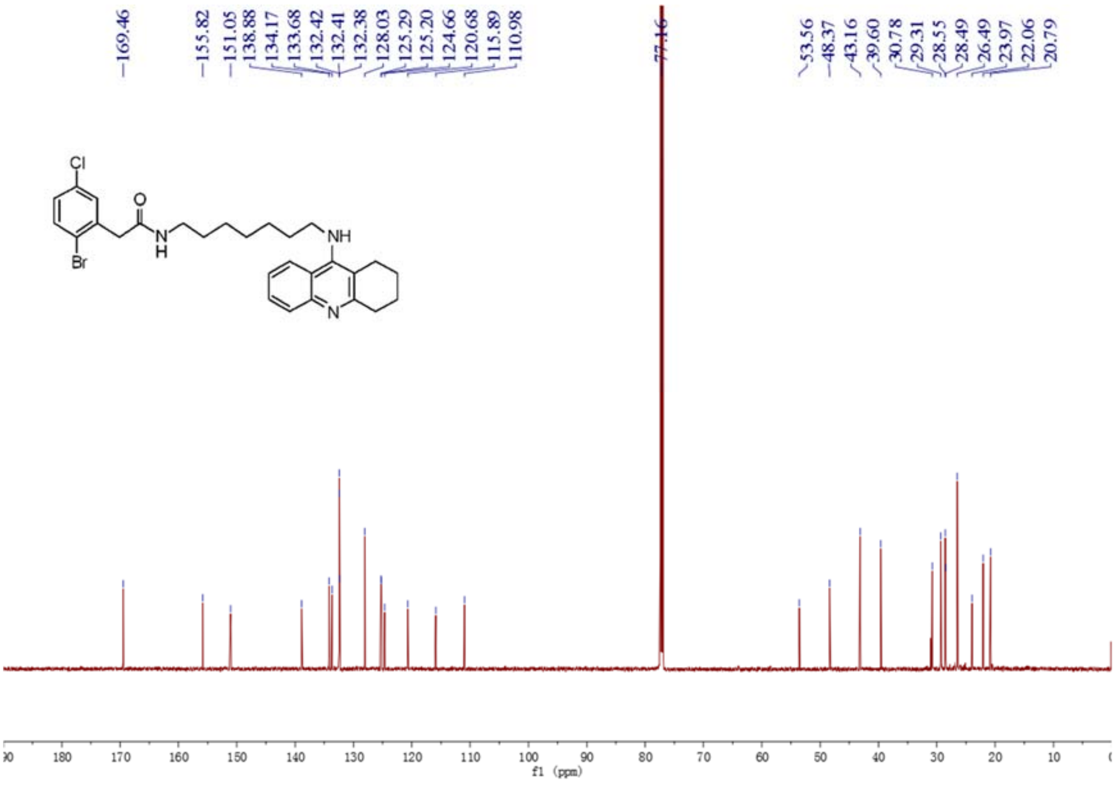

HR-MS (ESI) spectrum of **12k**

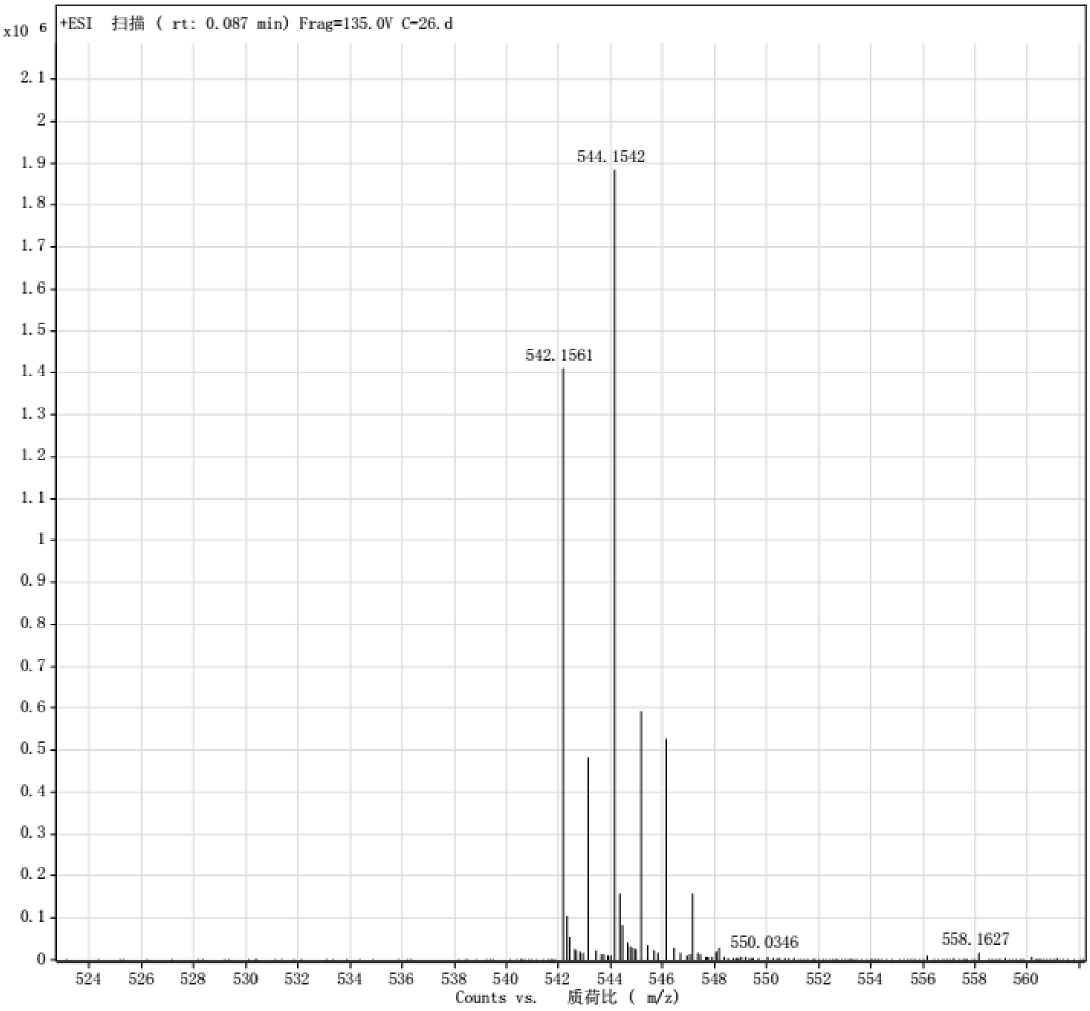

^{1}H NMR spectrum of **12l**

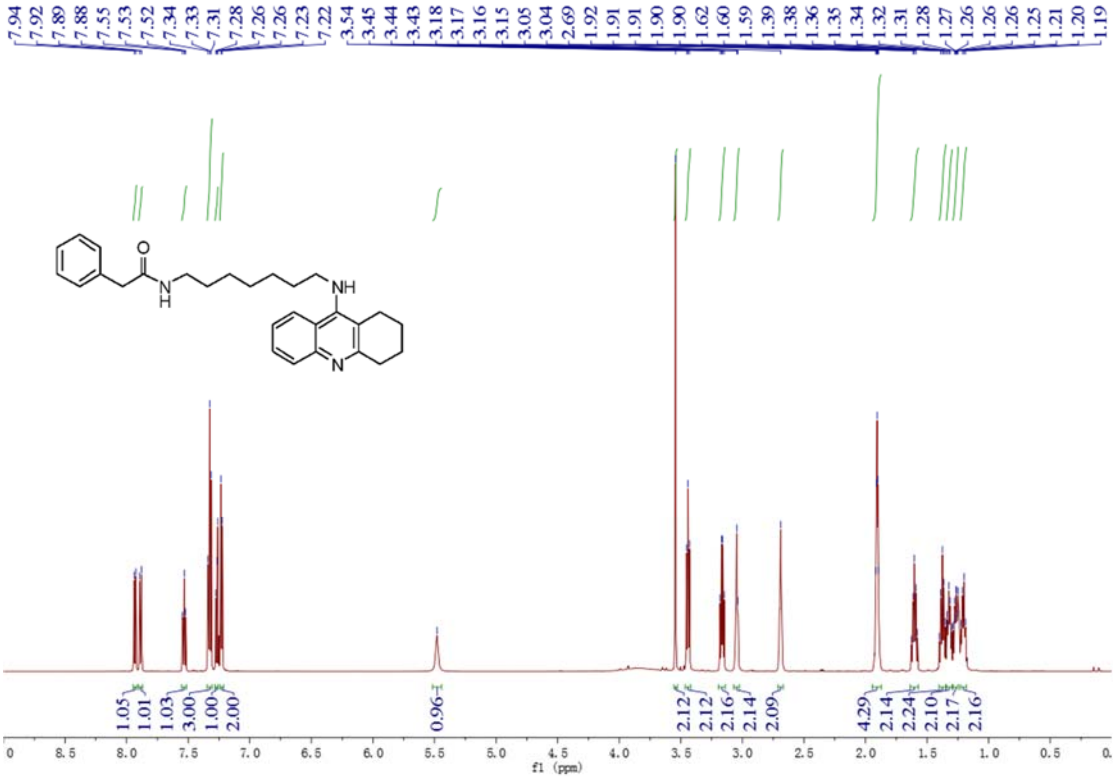

^{13}C NMR spectrum of **12l**

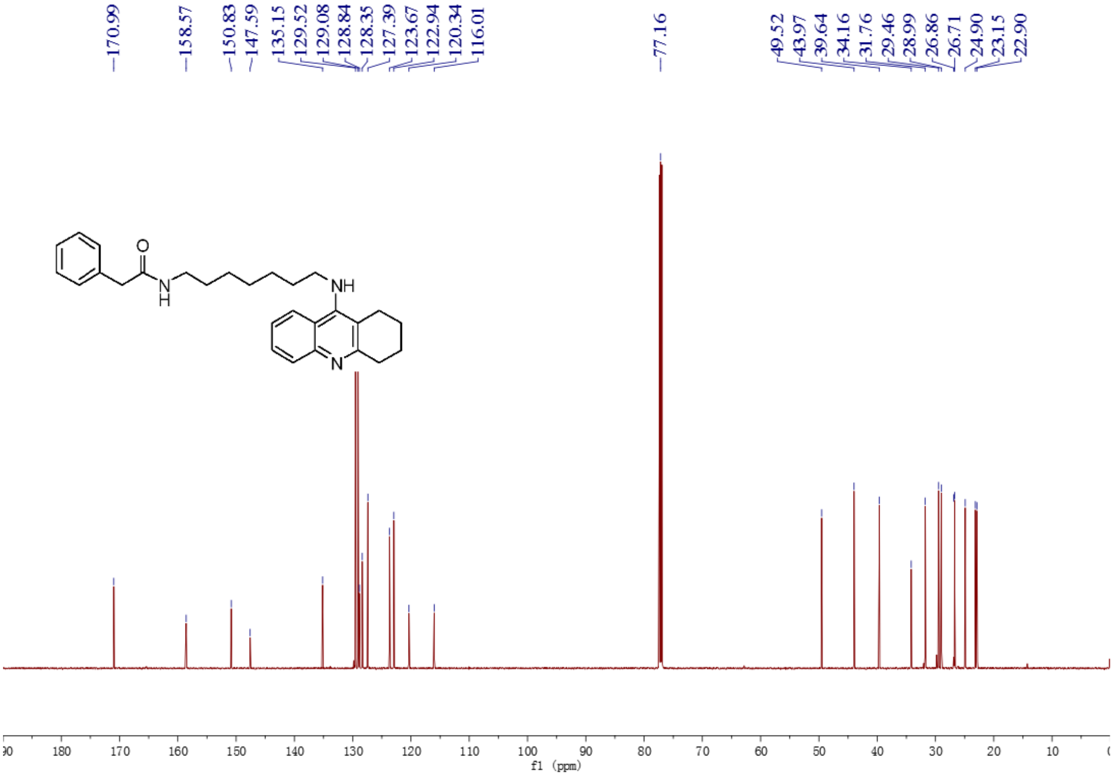

HR-MS (ESI) spectrum of **12l**

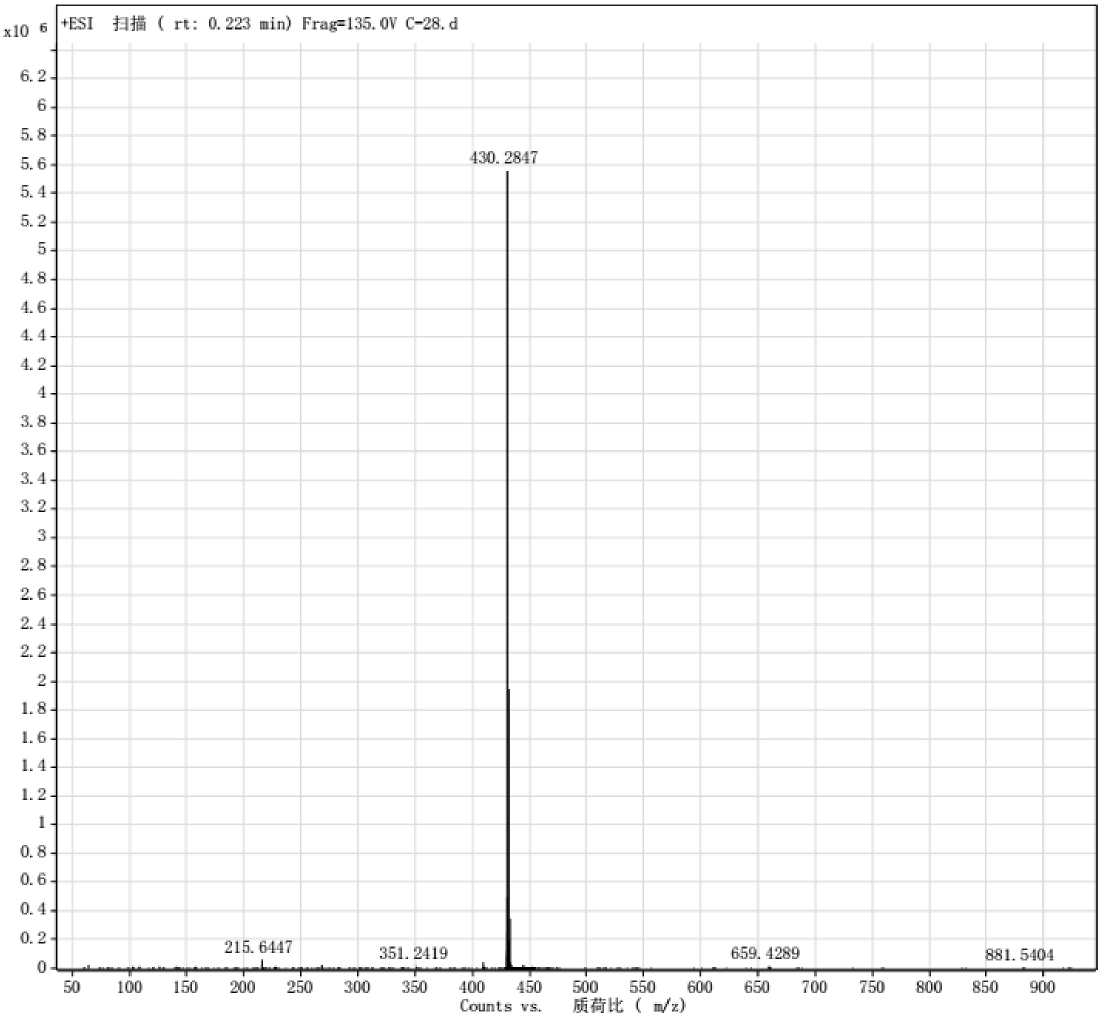

Supplement: Supplementary file 1 [file marinedrugs-16-00293-s001.pdf]
